# Single-dose administration of therapeutic divalent siRNA targeting MECP2 prevents lethality in an MECP2 duplication mouse model

**DOI:** 10.1038/s41467-026-75611-7

**Published:** 2026-07-21

**Authors:** Vignesh N. Hariharan, Ashley Summers, Amy E. Clipperton-Allen, Jillian Caiazzi, Samuel R. Hildebrand, Daniel O’ Reilly, Qi Tang, Zachary Kennedy, Dimas Echeverria, Nicholas McHugh, David Cooper, Jacquelyn Sousa, Chantal Ferguson, Hassan H. Fakih, Laurent Bogdanik, Monica Coenraads, Anastasia Khvorova

**Affiliations:** 1 RNA Therapeutic Institute, University of Massachusetts Chan Medical School, Worcester, United States of America; 2 The Jackson Laboratory, Bar Harbor, United States of America; 3 The Rett Syndrome Research Trust, Trumbull, United States of America

**Keywords:** RNAi therapy, Developmental disorders

## Abstract

MECP2 duplication syndrome (MDS) is a rare X-linked neurodevelopmental disorder caused by duplications of the dosage-sensitive methyl-CpG-binding protein 2 (MECP2) gene. Developing therapies for MDS is challenging due to the variability in MECP2 expression among patients and the risk of inducing Rett syndrome through excessive pharmacological intervention. Reducing dosage to optimize silencing often compromises durability and necessitates increased dosing frequency. We present here a series of fully chemically modified small interfering RNAs (siRNAs) designed for isoform-selective and total *Mecp2* silencing. Among these, we identify six lead siRNA candidates across two chemical scaffolds, achieving targeted total *Mecp2* expression reductions ranging from 25% to 75%, sustained for at least four months following a single administration. The efficacy and safety of human ortholog silencing are evaluated using a mouse model with ~8-fold human *Mecp2* transgene expression. In this severe duplication model, a single dose of the total isoform-silencing siRNA rescues early mortality and select behavioral impairments. Overall, this study introduces preclinical candidates for the treatment of MDS. Furthermore, it establishes a target selection strategy applicable to other dosage-sensitive gene imbalances.

## Abstract (web summary)

Here authors designed a durable, fully chemically modified therapeutic siRNA targeting MECP2 for MECP2 Duplication Syndrome. In a severe mouse model, a single dose of lead siRNA modified behavioral signs and rescued lethality for up to a year.

## Introduction

Methyl-CpG-binding protein 2 (MECP2) duplication syndrome (MDS) is a rare X-linked neurodevelopmental disorder resulting from the duplication of the Xq28 chromosomal region, which includes the dosage-sensitive *MECP2* gene. MDS presents with a broad spectrum of severe clinical manifestations^1^ with no cure for the disease. Mosaic loss-of-function mutations (due to X-chromosome inactivation in females) in MECP2 lead to Rett syndrome (RTT), highlighting the necessity of precise MECP2 dosage regulation for maintaining normal neurological function^2^. While both MDS and RTT are X-linked disorders, RTT is caused by heterozygous loss-of-function variants in MECP2 and predominantly affects females. In males, MECP2 loss-of-function is typically lethal due to the absence of a second X chromosome to provide functional MECP2, although rare cases of male RTT have been documented^3,4^. In contrast, MDS results from duplications of large regions of Xq28, including the MECP2 gene. MDS primarily affects males, as most females usually show preferential inactivation of the X chromosome carrying the duplication. Given the genetic basis of MDS, nucleic acid-based therapeutics (NATs), such as antisense oligonucleotides (ASOs) and small interfering RNAs (siRNAs), represent promising therapeutic avenues. These modalities enable precise modulation of gene expression by inducing transcriptional silencing through RNA interference (RNAi). Among these, an ASO named ION440 is the most advanced candidate currently under development for MDS, with a single dose providing up to twelve weeks of MECP2 modulation, with transcriptional correction for up to 16 weeks post administration. Clinical trials for this therapeutic candidate began in 2024 under the sponsorship of Ionis Pharmaceuticals (NCT06430385)^5,6^.

A key challenge in MECP2 therapeutics is precise dose titration to avoid excessive silencing, which may induce RTT. Female RTT patients, heterozygous for loss-of-function MECP2 mutations, exhibit a mosaic of wildtype and mutant *MECP2*-expressing cells^7^, raising questions about whether uniform over-silencing using NATs could replicate RTT’s mosaic pathology. The pharmacodynamics of ASOs and siRNAs are inherently dose-dependent, with higher doses leading to greater tissue accumulation and sustained gene silencing^8–10^. However, reducing the dosage to avoid over-silencing necessitates more frequent intrathecal administration, which in turn increases the risk of adverse events such as infection, cerebrospinal fluid leakage, neurovascular injury, and nerve or spinal cord damage^11–14^. The variability in MECP2 expression levels among patients further complicates dosing strategies, necessitating individualized treatment regimens^15^.

The *Mecp2* gene comprises four exons and three introns, with alternative splicing giving rise to two primary isoforms, *Mecp2 E1* and *Mecp2 E2*. Despite differences in their N-terminal regions due to alternative splicing, both isoforms share identical DNA-binding and functional domains. MECP2 E1 comprises the majority of MECP2 in neurons and has a lower DNA-binding affinity compared to MECP2 E2^16^. The two isoforms are also expressed differentially depending on the stage of development, region and cell type of the brain^17^. Isoform-specific deletion studies have shown that MECP2 E1 ablation is sufficient to cause RTT-like symptoms in mice, while MECP2 E2 ablation was well tolerated in the context of RTT^18,19^. These observations, together with the fact that MECP2 E1 mutations have been found in RTT patients while MECP2 E2 mutations have not been seen in the clinic^20–22^ provide further evidence that MEPC2 E1 loss-of-function is the main driver of the RTT phenotype. However, transgenic expression studies have demonstrated that either MECP2 E1 or MECP2 E2 can rescue the lethal phenotype and behavioral abnormalities observed in MECP2-null mice, with the extent of behavioral and lifespan rescue related to expression levels of MECP2 regardless of isoform identity^17,23–26^. Similarly detailed information regarding the role of MECP2 isoforms is lacking for MDS. Due to the large regions of duplication, spanning several genes, MDS patients express all MECP2 isoforms in excess. The functional redundancy between E1 and E2 isoforms suggests that in the context of MDS, selective silencing of one isoform could reduce the overall MECP2 expression level while preserving therapeutic efficacy and avoiding the need for dose titration.

A potential explanation for the two seemingly contradictory observations, i.e., that i) MECP2 E1 deletion is sufficient to cause RTT and ii) RTT can be rescued by transgenic expression of MECP2 E2, is that natural levels of MECP2 E1 and the translation efficiency of E1 mRNA are greater than that of MECP2 E2, resulting in 90% of MECP2 expression in the brain being the E1 isoform^27^. Therefore, studies showing that E1 ablation alone is sufficient to cause RTT may be confounded by differences in natural expression levels between the two isoforms, leading to the potentially erroneous conclusion that the two isoforms are not functionally redundant. In the context of MDS, the roles and contributions of each isoform have not been explored; it is not known whether MECP2 E1 or MECP2 E2 alone can result in MDS when overexpressed.

An emerging class of NATs particularly well-suited to addressing the challenge of tunable yet durable silencing of *Mecp2* involves fully chemically modified siRNAs. These duplex RNA-like molecules are engineered to harness the endogenous microRNA-mediated gene silencing machinery, known as the RNA-induced silencing complex (RISC), to selectively bind to and degrade the target mRNA of disease-causing genes. These siRNAs are chemically modified to enhance plasma and tissue nuclease resistance by substituting every 2’ ribose position with 2’-fluoro or 2’-O-methyl groups, replacing the guide strand 5’-phosphate with 5’-vinylphosphonate, and protecting the 5’ and 3’ termini of both strands with phosphorothioate linkages instead of the naturally occurring phosphodiester backbone^28–30^. siRNA-mediated gene silencing is catalytic, allowing a single siRNA-loaded RISC to bind and cleave multiple target mRNA molecules in the cytoplasm sequentially^31^. We showed previously that divalent, fully chemically modified asymmetric siRNAs achieve broad central nervous system (CNS) distribution in mice and non-human primates (NHPs), enabling sustained gene silencing with a wide therapeutic index^8^. Increased CNS retention and distribution likely stem from reduced CSF clearance, while phosphorothioate single-stranded regions may enhance cellular uptake efficiency^30^.

The chemical modification of the siRNA scaffold provides a second approach to optimizing gene silencing. Due to extensive protein-RNA contacts and structural constraints within the siRNA-loaded RISC complex, 2’ ribose modifications must be strategically placed across the siRNA backbone to ensure that the local and global structure of the modified siRNA remains compatible with functional RISC loading. The specific positions of these chemical modifications—such as 2’-fluoro, 2’-O-methyl, and phosphorothioates—collectively constitute the modification pattern of the siRNA scaffold^29^. While certain 2’-ribose modification positions have traditionally been viewed as incompatible due to their negative impact on siRNA efficacy^32–34^, these positions can be reinterpreted as tunable sites. By strategically placing modifications at these sites, it is possible to fine-tune the efficacy of RISC loading and target mRNA cleavage, thus achieving a more controlled and durable gene silencing effect.

In this study, we developed fully chemically modified divalent siRNA therapeutic candidates capable of silencing total *Mecp2* or *Mecp2 E1* specifically. Testing both E1 isoform selective and total isoform silencing will provide preliminary insights into the contribution of the E1 isoform to the disease in this model.

We evaluated these siRNA sequences in wild-type mice using two previously established 2’-ribose modification patterns, which have been shown to modulate siRNA efficacy across multiple sequences^34–36^. In a humanized transgenic mouse model of MECP2 duplication syndrome (MDS)^37^, which expresses 3–5-fold the endogenous MECP2 levels, isoform-selective silencing produced initial improvements in phenotypes but failed to achieve long-term disease modification with a single administration, while total *Mecp2* silencing modified disease for at least forty weeks following a single administration. The identified pre-clinical candidates are cross-reactive to seven potential preclinical species, paving the way for formal pre-clinical development.

## Results

### In vitro screen identifies potent multi-species targeting lead siRNAs

A comprehensive bioinformatic analysis of *Mecp2* transcripts, including the 5’ and 3’ untranslated regions (UTRs) and the open reading frame (ORF), was conducted to identify potential active siRNA sequences, following established guidelines^38,39^. Twenty-four top-scoring sequences, including those human-mouse cross-reactive sites, were synthesized for in vitro screening. Given that chemical modifications of siRNAs can significantly restrict the RISC-compatible sequence space, all siRNAs were synthesized using fully chemically modified, asymmetric siRNA scaffolds with 2’-fluoro, 2’-O-methyl, and phosphorothioate modifications, as previously described^40^. To facilitate gymnotic uptake into cells without transfection reagents, the 3’ terminus of the passenger strand was covalently linked to cholesterol. Details of all sequences and chemical modification patterns used in this study are provided in the supplementary material (Table S1).

**Fig. 1:**
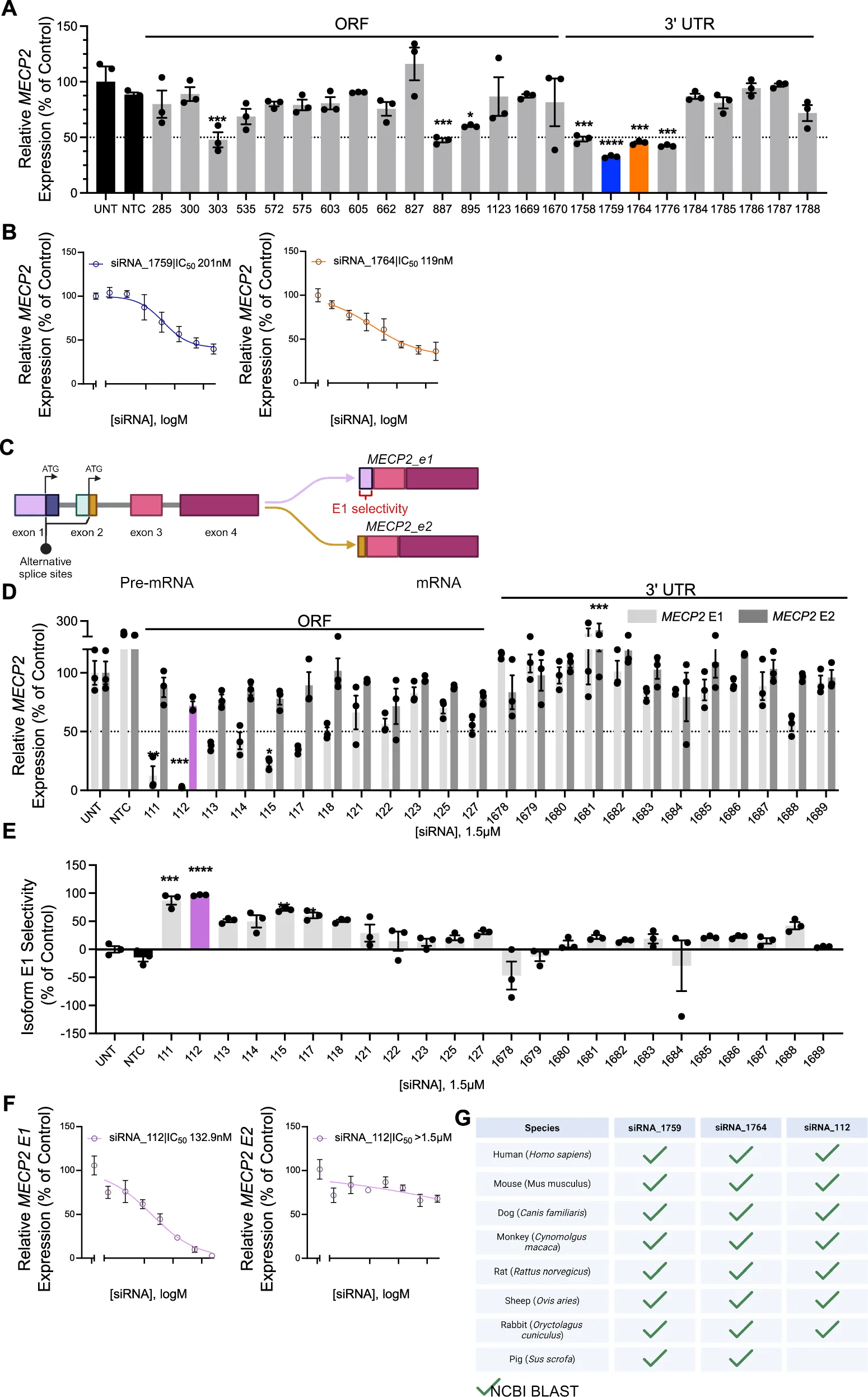
Lead total and isoform selective MECP2 silencing siRNAs identified through in vitro screens. **A** Relative expression of total human *MECP2* mRNA in HeLa cells 72 h after treatment with non-isoform selective *MECP2* targeting siRNA at a single concentration. Lead compounds selected for further analysis are shown as blue (1759) and orange (1764) bars. **B** Relative expression of total human *MECP2* mRNA in HeLa cells 72 h after treatment with total isoform silencing lead siRNAs 1759 (blue) and 1764 (orange) across a range of concentrations. **C** Schematic of alternative splicing of the MECP2 pre-mRNA. **D** Relative expression of *MECP2-E1* (light gray bars) and *MECP2-E2* (dark gray bars) mRNA in HeLa cells 72 h after treatment with E1 isoform selective siRNA at a single concentration. Lead compound (112) selected for further analysis is shown in purple bars. **E** Isoform E1 selectivity of siRNA compounds screened in (**D**). **F** Relative expression of *MECP2-E1* mRNA in HeLa cells 72 h after treatment with E1 isoform selective siRNA 112 across a range of concentrations. **G** Results of cross-reactivity analysis of the targeting regions of lead siRNAs across the genomes of humans and pre-clinical species using Basic Local Alignment Search Tool (BLAST) from the National Center for Biotechnology Information (NCBI) (green checkmarks) database. Gene expression was measured using the QuantiGene™ Singleplex Assay Kit. Data represented as mean ± s.e.m. of three independent replicates. Statistical analysis performed using Ordinary one-way ANOVA (one-tailed) with Dunnett’s adjustment for multiple comparisons for (**A**, **D**, E) (*-*p* < 0.05, **-*p* < 0.01, ***-*p* < 0.001, ****-*p* < 0.0001). Exact *p*-values have been provided in the statistical analysis tables in the supplementary table S3. **C**, **G** were created in BioRender. Fakih, H. (2026) https://BioRender.com/srdyn7n.

siRNA candidates targeting all *Mecp2* mRNA isoforms were screened in vitro at a single concentration of 1.5 μM in human HeLa cells (Fig. 1A) and mouse N2A cells (Supplemental Fig. S1A), with gene expression assessed using the Quantigene 2.0 branched DNA assay. Quantigene probesets were designed to detect all MECP2 mRNA isoforms. A non-targeting control siRNA (NTC) served as a negative control in all in vitro screens. siRNAs 1759 and 1764 exhibited significant total *Mecp2* silencing and were selected for further potency evaluation using a seven-point dose-response curve in both HeLa cells (Fig. 1B) and N2A cells (Supplemental Fig. S1B). The IC_50_ values for siRNA_1759 and siRNA_1764 were 201 nM and 119 nM, respectively, in passive uptake in HeLa cells, with comparable potency observed in N2A cells. These results indicate that these siRNAs are suitable candidates for further in vivo evaluation.

While studies have clearly shown that loss-of-function of the E1 isoform is necessary and sufficient to cause RTT in animals^18,19^ and in humans^20–22^, a similar understanding of the role of the E1 isoform in MDS is lacking. The two major MECP2 isoforms, E1 and E2, may have sufficient functional overlap to provide redundancy^23^. In healthy neurons, both isoforms are expressed at levels that maintain optimal total MECP2. In MDS, where duplications typically span multiple genes, both E1 and E2 are overexpressed, resulting in elevated MECP2 levels. In order to evaluate whether MDS is driven by the E1 isoform alone, we also developed siRNA sequences that target isoform E1 but not E2.

To achieve this, we designed a panel of human and human-mouse cross-reactive siRNAs targeting exon 1, which is present in isoform E1 but absent in E2 (Fig. 1C). HeLa cells were treated with 1.5 μM of cholesterol-conjugated siRNAs designed to target MECP2 E1 without affecting E2. Expression levels of isoforms E1 and E2 were then measured using custom isoform-specific probes with the Quantigene 2.0 branched DNA assay. siRNA_112 provided maximal silencing of E1 (Fig. 1D), with a ~ 95% selectivity for E1 over E2 (Fig. 1E). We were unable to find any siRNAs that specifically silenced the E2 isoform without affecting E1 (Supplementary Fig. S1C). This result was corroborated in seven-point dose-response assays, where siRNA_112 silenced *Mecp2 E1* mRNA with an IC_50_ of 133 nM in HeLa cells (Fig. 1F) and 263 nM in N2A cells (Supplemental Fig. S1D), with minimal impact on *Mecp2 E2* mRNA expression.

The lead siRNAs—1759, 1764, and 112—identified from the in vitro screens targeted regions of *Mecp2* mRNA that were 100% conserved across multiple species, including mouse, dog, cynomolgus monkey, rat, sheep, rabbit, and pig, as determined by the Basic Local Alignment Search Tool (BLAST) from the National Center for Biotechnology Information (NCBI). (Fig. 1G). The cross-species homology of these lead compounds ensures that formal IND-enabling preclinical studies will not be limited by the availability of on-target pharmacology models.

### Lead MECP2-targeting siRNAs tune MECP2 levels in wild-type mice for up to four months

**Fig. 2:**
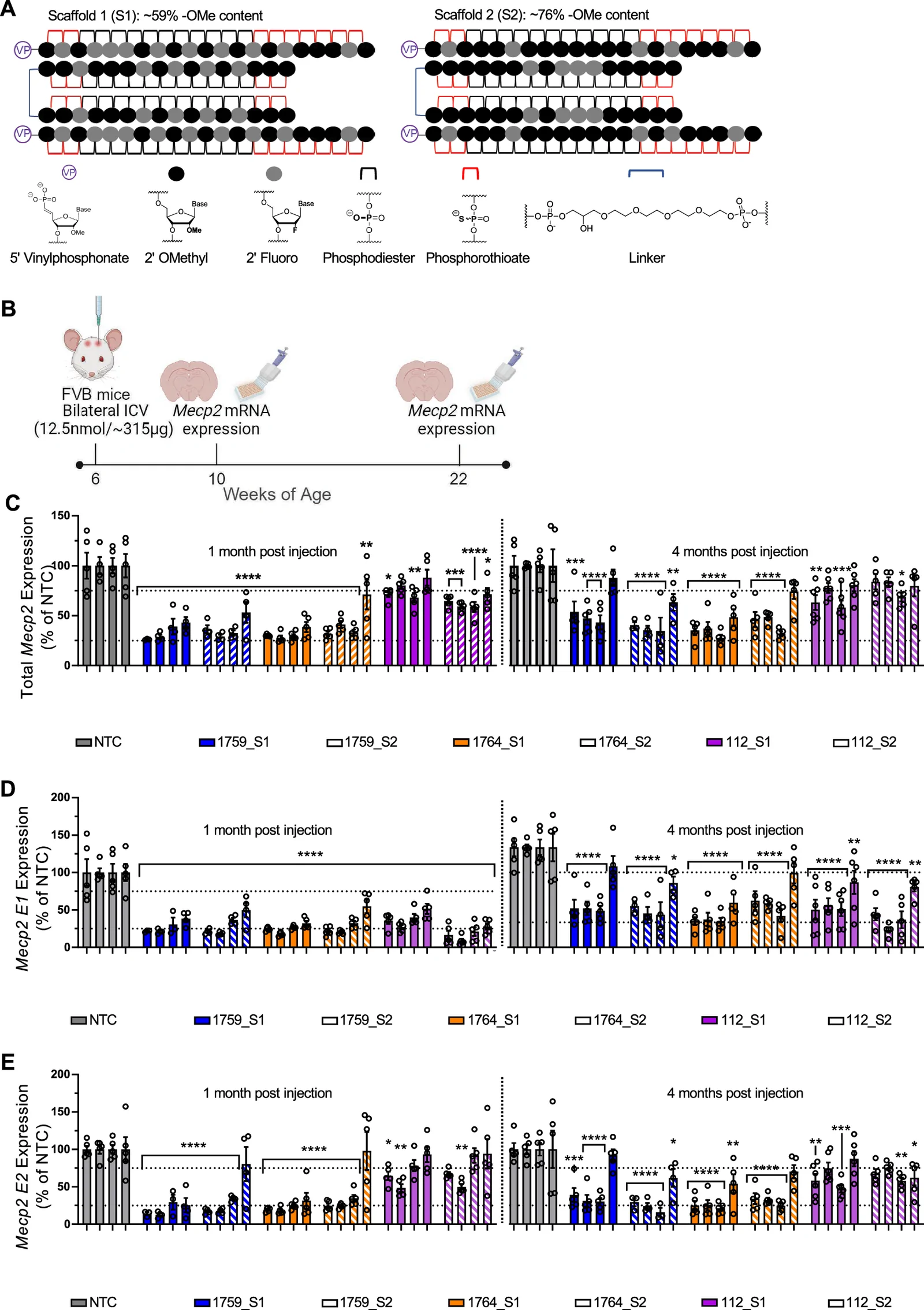
Lead compounds show potent and sustained modulation of *Mecp2* mRNA expression in the wild-type mouse brain. **A** Schematic of divalent scaffolds, modification patterns and chemical structures of relevant modifications used in this experiment. **B** Illustration of study design including dosage, route of administration, timeline and readouts. **C** Total *Mecp2* mRNA expression levels, **D** *Mecp2-E1* mRNA expression levels and **E** *Mecp2-E2* mRNA expression levels in the cortex, striatum, hippocampus and cerebellum of wild-type FVB mice injected with 12.5 nmol (~315 µg) of siRNA variants. Gene expression was measured at one month and four months post-injection using the QuantiGene™ Singleplex Assay Kit. Data in **C**–**E** represented as mean ± s.e.m. of individual animals (*n* = 3 mice for 1759_S1at 1 month in cortex, *n* = 4 mice for 1759_S1 at 1 month in all other brain regions; *n* = 5 mice for 1764_S1, 112_S1, 1764_S2, and 112_S2 at 1 month in all brain regions; *n* = 4 mice for 1759_S2 at 1 month in all brain regions; *n* = 4 mice for NTC at 1 month in striatum, *n* = 5 mice for NTC at 1 month in all other brain regions; *n* = 5 mice for 1759_S1, 1764_S1, 1764_S2, 112_S2 and NTC at 4 months for all brain regions; *n* = 3 mice for 1759_S2 at 4 months for cortex, *n* = 4 mice for 1759_S2 at 4 months in all other brain regions; *n* = 6 mice for 112_S1 at 4 months in all brain regions). Statistical analysis performed using two-way ANOVA with Dunnett’s adjustment for multiple comparisons (*-*p* < 0.05, **-*p* < 0.01, ***-*p* < 0.001, ****-*p* < 0.0001). Exact *p*-values have been provided in the statistical analysis tables in the supplementary table S3. **B** was created in BioRender. Fakih, H. (2026) https://BioRender.com/srdyn7n.

To evaluate the in vivo potency and duration of effect for lead siRNAs, we synthesized these siRNAs using a validated divalent brain delivery scaffold, which has been shown to enable deep brain distribution and multi-month silencing across various animal models^8^. The divalent siRNAs, composed of two siRNA passenger strands linked by a tetraethylene glycol linker and hybridized to the guide strand, were synthesized in two distinct modification patterns: scaffold 1 ( ~ 59% 2’-O-methyl content) and scaffold 2 ( ~ 76% 2’-O-methyl content), both of which have been previously demonstrated to modulate silencing for other siRNA sequences^33,35,36^. Given that most clinically approved siRNAs feature a higher 2’-O-methyl to 2’-fluoro ratio^32,41^, likely due to the increased nuclease stability of 2’-O-methyl^42^, we aimed to determine whether the 2’-O-methyl-rich scaffold 2 would offer greater durability for *Mecp2* silencing in the brain. The guide strand’s 5’-phosphate was stabilized using vinylphosphonate (VP) for enhanced binding affinity to the Mid domain of Argonaute-2 and enhanced phosphatase stability, while the ends of both strands were modified with phosphorothioate linkages for enhanced nuclease stability^32,41,43^ (Fig. 2A).

Female wildtype FVB mice were administered 12.5 nmol (~315 µg) of divalent siRNAs or divalent non-targeting control (NTC) siRNA via intracerebroventricular (ICV) injection at six weeks of age. Mice were sacrificed at one and four months post-injection, and total *mecp2*, *mecp2-E1*, and *mecp2-E2* mRNA expression levels were analyzed in the cortex, striatum, hippocampus, and cerebellum using the Quantigene 2.0 branched DNA assay (Fig. 2B). While the Quantigene probesets for detecting E1 and E2 isoforms were custom-designed for selectivity, the total *mecp2* probeset detects a broader range of *mecp2* mRNAs. Consequently, in this assay, the sum of E1 and E2 isoform expression cannot be used to infer total *mecp2* mRNA levels. Instead, the E1 and E2 probesets were employed to assess siRNA selectivity, while the total *mecp2* probeset served as the primary metric of clinical utility.

At one-month post-injection, total *mecp2* mRNA levels in the cortex, striatum, and hippocampus of mice treated with non-isoform-selective siRNAs (1759_S1, 1759_S2, 1764_S1, and 1764_S2) were reduced to ~25–35% of NTC controls (*p* < 0.0001), with no significant differences observed across these brain regions (Fig. 2C). Silencing in the cerebellum was less pronounced and more variable, due to lower siRNA accumulation in this region of rodent brains^8^. In the cerebellum, mice treated with 1759_S1 and 1764_S1 showed mean total *mecp2* expression levels of 43% (*p* < 0.0001) and 38% (*p* < 0.0001), respectively. In contrast, the 2’-O-methyl-rich scaffold 2 siRNAs (1759_S2 and 1764_S2) resulted in higher *mecp2* levels of 53% (*p* < 0.0001) and 71% (*p* < 0.01), respectively (Fig. 2C).

At four months post-injection, 1764_S1 retained near-maximal silencing in all brain regions tested, with total *mecp2* mRNA levels between 35–48% (*p* < 0.0001) across all brain regions tested, making it the most potent compound for total *mecp2* mRNA silencing in this study. In contrast, while 1759_S1, 1759_S2, and 1764_S2 maintained *mecp2* mRNA levels at ~35–45% of NTC controls in the cortex, striatum, and hippocampus (*p* < 0.001 to *p* < 0.0001), cerebellar *mecp2* mRNA levels were 88% (*p* = 0.7), 63% (*p* < 0.01), and 74% (*p* < 0.07), respectively, indicating reduced durability compared to 1764_S1 (Fig. 2C).

For isoform E1-selective siRNAs (112_S1 and 112_S2), total *mecp2* mRNA levels were reduced to 59–72% across brain regions, with this effect being maintained for at least four months. While 112_S2 showed better performance at one month, 112_S1 demonstrated superior durability for the duration tested (Fig. 2C).

Isoform E1 mRNA levels in siRNA-treated mice at one month post-injection were ~16–50% of NTC across all brain regions for all compounds tested (*p* < 0.0001 for all groups). These results indicate that 112_S2 was the most potent suppressor of *mecp2-E1* transcription in one month. Differences in efficacy between scaffolds S1 and S2 for 1759 and 1764 were minimal. Differences between scaffolds were more pronounced in the cerebellum, confirming that scaffold S2 was less active than S1 for 1759 and 1764 siRNAs, while 112_S2 remained the most effective at silencing E1 mRNA in the cerebellum (Fig. 2D).

At four months post-injection, E1 mRNA levels across brain regions and compounds were 17–44%. Although 112_S2 showed greater silencing at one month, 1764_S1 exhibited slightly, though not statistically significant, better durability in the cortex and hippocampus. This difference was more evident in the cerebellum, indicating that 1764_S1 provided the most durable silencing of *mecp2-E1* (Fig. 2D).

At one month post-injection, suppression of *mecp2* isoform E2 mRNA was notably higher than that of E1 or total *mecp2* for the non-isoform-selective siRNAs ( < 25% of NTC). E1-selective siRNAs 112_S1 and 112_S2 also reduced E2 expression levels in the cortex and striatum but not the hippocampus and cerebellum, indicating that these siRNAs did not achieve full E1 isoform selectivity at the tested doses in certain brain regions. Similar to total and E1 isoform *mecp2* mRNA levels, the lower accumulation of divalent siRNA in the rodent cerebellum allowed for clearer differentiation between scaffolds S1 and S2, with scaffold S2 showing lower efficacy in this region (Fig. 2E).

At four months post-injection, 112_S1 and 112_S2 had some impact on E2 mRNA levels, but this effect did not reach statistical significance except in the hippocampus. The effects of non-selective 1759 and 1764 were similar to those observed at the one-month time point. In the cerebellum, the differences between siRNAs were more pronounced, with no significant silencing observed in mice treated with 1759_S1, 112_S1, or 1764_S2 (Fig. 2E).

**Fig. 3:**
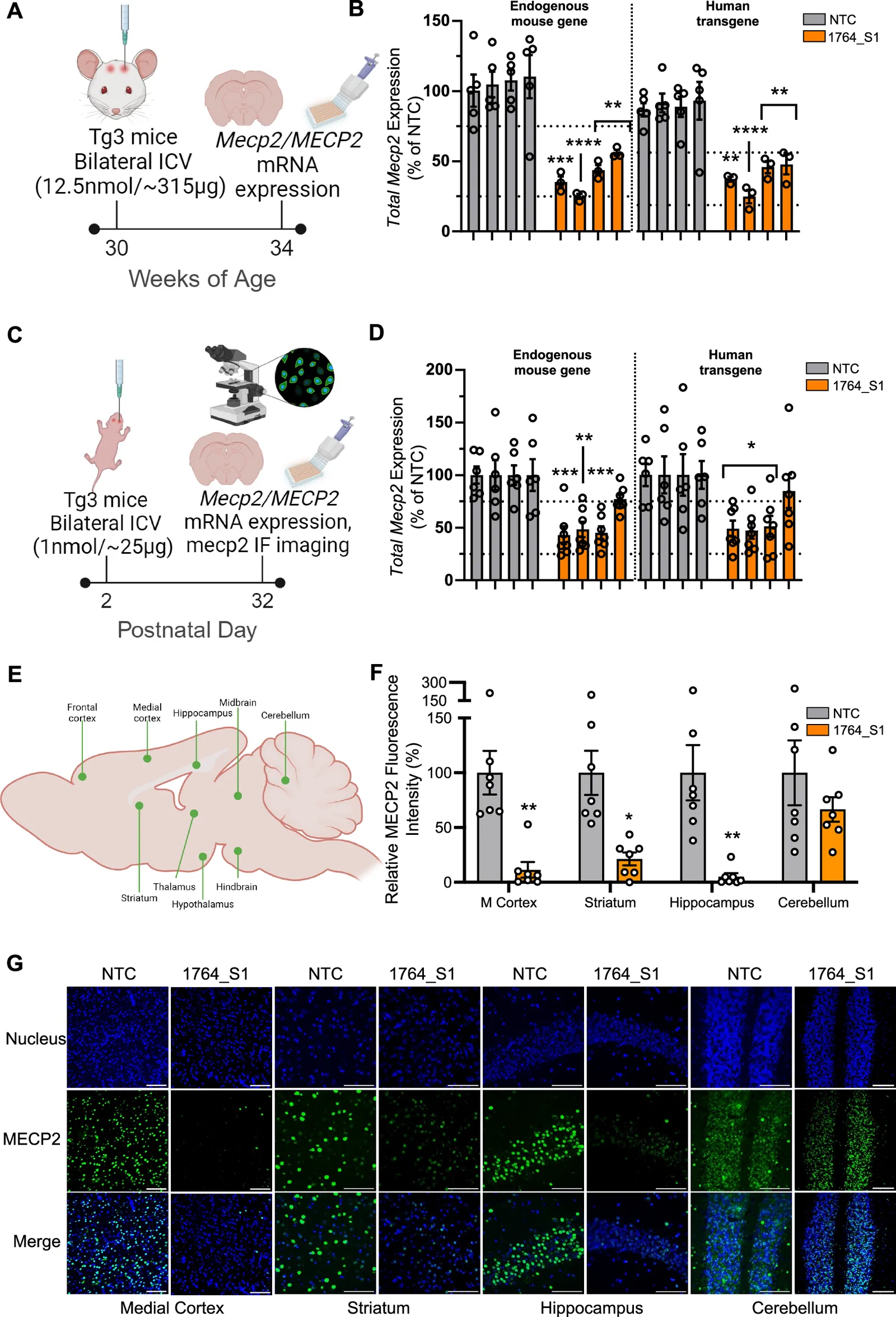
1764_S1 is active against mouse and human MECP2 in the adult and neonatal Tg3 brain. **A** Illustration of study design including dosage, route of administration, timeline and readouts in adult Tg3 mice. **B** Total mouse Mecp2 and human mouse MECP2 mRNA expression levels in the cortex, striatum, hippocampus and cerebellum of adult Tg3 mice injected with 12.5 nmol (~315 µg) of 1764_S1 (*n* = 5 mice for NTC and *n* = 3 mice for 1764_S1). **C** Illustration of study design including dosage, route of administration, timeline and readouts in neonatal Tg3 mice. **D** Total mouse Mecp2 and human mouse MECP2 mRNA expression levels in the cortex, striatum, hippocampus and cerebellum of neonatal Tg3 mice injected with 1 nmol (~25 µg) of 1764_S1 (*n* = 6 for NTC and *n* = 7 for 1764_S1). **E** Illustration of brain regions of interest identified for relative quantification of immunofluorescence from mice shown in (**C**). **F** Percentage of nuclear MECP2 fluorescence intensity in the medial cortex (M. cortex), striatum, hippocampus and cerebellum of neonatal mice shown in (**C**) (*n* = 7 mice per group). **G** Representative immunofluorescence images at 40x magnification of samples quantified in (**F**), scale bar = 100 µm. Gene expression was measured using the QuantiGene™ Singleplex Assay Kit. Data represented as mean±s.e.m. of individual animals. Immunofluorescence imaging was performed on sagittal brain sections stained with DAPI and anti-MECP2 antibody and imaged using a Leica DMi8 widefield microscope. Quantification of MECP2 nuclear intensity was performed using a custom ImageJ script. Statistical analysis performed using two-way ANOVA with Dunnett’s adjustment for multiple comparisons (*-*p* < 0.05, **-*p* < 0.01, ***-*p* < 0.001, ****-*p* < 0.0001). Exact *p*-values have been provided in the statistical analysis tables in the supplementary table S3. **A**, **C**, **E** were created in BioRender. Fakih, H. (2026) https://BioRender.com/srdyn7n.

In general, all non-selective compounds maintain robust silencing of total *mecp2* with less than 30–40% of total mRNA remaining at up to four-months post injection. There were no significant differences between scaffolds except for 1764_S1, showing higher potency in the cerebellum compared to 1764_S2. Based on this data, 1764 S1 was selected for further evaluation in disease models. The isoform-selective compound 112_S1 was selected as a second lead compound to evaluate the effects of E1 silencing with minimal perturbation of E2 levels. We have previously seen differences between protein and mRNA silencing for CNS targets due to the presence of inaccessible nuclear pools of target mRNA^44^. Indeed, we later evaluated the MECP2 silencing in the context of an MDS animal model and confirmed that ~ 60–70% mRNA silencing corresponds to highly potent protein reduction of ~ 80–90% (Fig. 3).

Since studies have shown that even reductions in MECP2 levels of 20-30% in wildtype mice result in anxiety-like phenotypes and other behavioral deficits that mimic some of the phenotypes of MECP2-null mice^45^, behavioral analysis in WT mice should be conducted in future studies to fully understand the effect of the siRNAs developed here.

### Activity of 1764_S1 against human MECP2 confirmed in a severe transgenic mouse model of MDS

In order to evaluate the efficacy of lead siRNAs and study their effect on disease phenotypes, we selected a well-characterized human transgenic MDS model developed by the Zoghbi lab^37^—FVB-Tg(MECP2)3Hzo/J (Tg3) obtained from the Jackson Laboratory and maintained on the FVB background (strain #008680), which expresses *Mecp2* at approximately three- to five-fold the wild-type level. These transgenic lines were created by microinjection of a 99 kb human PAC clone carrying the entire *Mecp2* locus, ensuring expression of both isoforms, including untranslated regulatory regions. Additionally, the transgene is inserted on the X-chromosome, mimicking the X-linked presentation of the disease in humans.

We utilized an existing colony of Adult Tg3 mice (30 weeks of age) to assess the efficacy of the siRNAs. Mice were injected with 12.5 nmol (~315 µg) of 1764_S1 via bilateral intracerebroventricular (ICV) injection. The mice were sacrificed at 34 weeks of age, and their brains were analyzed for total MECP2 mRNA levels (Fig. 3A). Since these transgenic mice are hemizygous, containing both the endogenous mouse gene and the human transgene, we were able to measure the total *MECP2* mRNA of both species using species-specific probesets in the QuantiGene™ Singleplex branched DNA assay. As expected, total mouse *mecp2* mRNA levels in the cortex, striatum, hippocampus, and cerebellum were 35% (*p* < 0.001), 25% (*p* < 0.0001), 44% (*p* < 0.01), and 56% (*p* < 0.01) of non-targeting control (NTC) levels, respectively. Silencing of total human *Mecp2* mRNA was slightly lower, with levels of 50% (*p* < 0.01), 33% (*p* < 0.0001), 61% (*p* < 0.01), and 64% (*p* < 0.01) in the cortex, striatum, hippocampus, and cerebellum, respectively. These levels across all four brain regions were close to ~50% of NTC controls (Fig. 3B).

Given that MDS is a pediatric condition where early intervention is presumably more beneficial, we also assessed whether safe levels of silencing could be achieved in the smaller brains of neonatal mice. Tg3 mice at postnatal day 2 were injected with 1 nmol (~25 µg) of 1764_S1 via bilateral ICV injection, and the mice were sacrificed at postnatal day 32. Subsequently, total *MECP2* human and mouse mRNA levels were measured using the QuantiGene™ Singleplex branched DNA assay, and total MECP2 protein levels were analyzed by immunofluorescence in different brain regions (Fig. 3C). It should be noted that due to the small size of the neonatal mouse brain, the ICV injections were performed manually, leading to potential variability in the speed of injection and distribution of the siRNA throughout the brain. Total mouse *mecp2* mRNA levels in the cortex, striatum, hippocampus, and cerebellum were 43% (*p* < 0.001), 48% (*p* < 0.01), 45% (*p* < 0.001), and 77% (*p* = 0.3) of NTC controls, respectively. Total human *Mecp2* mRNA levels in the cortex, striatum, hippocampus, and cerebellum were 49% (*p* < 0.01), 47% (*p* < 0.01), 51% (*p* < 0.01), and 85% (*p* = 0.9) of NTC controls, respectively (Fig. 3D).

For immunofluorescence quantification, whole-brain tiled scans were collected at 40x magnification. Specific brain regions—namely the frontal cortex, striatum, medial cortex, hippocampus, thalamus, hypothalamus, midbrain, hindbrain, and cerebellum—were identified and cropped for analysis (Fig. 3E). Image thresholding was used to accurately separate the DAPI (nucleus) fluorescence signal from the background, followed by quantification using the ImageJ Measure Function. Individual nuclei were identified and counted as regions of interest (ROIs), and within each ROI, the integrated density of MECP2 immunofluorescence signal was evaluated and normalized to the number of cells in the view, defined by the number of DAPI-stained nuclei. On average, 500–1000 nuclei were identified for each brain region per animal.

MECP2 protein was significantly lower in 1764_S1-treated mice compared to NTC controls across most regions measured. The relative MECP2 abundance for each region, expressed as a percentage of NTC, was as follows: ~10% (*p* < 0.001) in the prefrontal cortex, ~21% (*p* < 0.001) in the striatum, ~11% (*p* < 0.001) in the medial cortex, ~5% (*p* < 0.0001) in the hippocampus, ~17% (*p* < 0.001) in the thalamus, ~24% (*p* < 0.05) in the hypothalamus, ~38% (*p* = 0.06) in the midbrain, ~36% (*p* < 0.05) in the hindbrain, and ~66% (*p* = 0.7) in the cerebellum (Fig. 1F, Supplementary Fig. S2F). Representative immunofluorescence images of each brain region from NTC and 1764_S1-treated mice are shown in Fig. 3G, Supplementary Figs. S2A–E. As observed in adult mice, 1764_S1 effectively reduced total *MECP2* mRNA and protein levels in multiple brain regions of Tg3 neonatal mice. The divalent siRNA efficiently distributes to both neuronal and glia cells and can produce measurable effects, silencing neuron and glia-selectively expressed genes below the lexvel of detection, as shown in previous studies^8,46^. While the MECP2 level of expression was higher in neurons than in glia, we expect the treatment with Divalent siRNA to be effective in silencing MECP2 in all cell types.

Interestingly, the level of protein silencing observed was much greater than the levels of mRNA silencing, indicating that there may be a fraction of transcribed mRNA retained in the nucleus and inaccessible to cytoplasmic siRNA activity, as has been reported for huntingtin, another CNS target^44^. More importantly, it underscores the importance of the MECP2 protein level as the primary measure of pharmacodynamics since quantification of the mRNA alone may underestimate the degree of silencing. Further work on dose titration using MECP2 protein levels as a readout of pharmacological activity is essential to the preclinical development of these siRNAs.

### A single administration of siRNA 112_S1 partially and siRNA 1764_S1 modifies disease in severe Tg3 MDS mice

To evaluate the efficacy of the lead compounds in modifying disease, we turned to a severe MDS model, the Tg3 mice, to evaluate the effectiveness of *Mecp2* silencing siRNA in disease modification. This model reportedly overexpresses the human *Mecp2* transgene at approximately 3.5-fold higher levels than wild-type FVB mice. The Tg3 mice were obtained from Jackson Laboratory (JAX) through Speed Expansion, using cryopreserved sperm from Tg3 males and oocytes from wild-type FVB females for the first cohort, with subsequent cohorts generated by mating mutant females with WT males. MECP2 overexpression was confirmed by JAX before the study, with Western blot data showing an ~8.4-fold increase in MECP2 expression compared to FVB wild-type controls (Supplementary Fig. S3). Behavioral assays were conducted using standard operating procedures established by JAX In Vivo Pharmacology Services. The study was conducted at JAX.

**Fig. 4:**
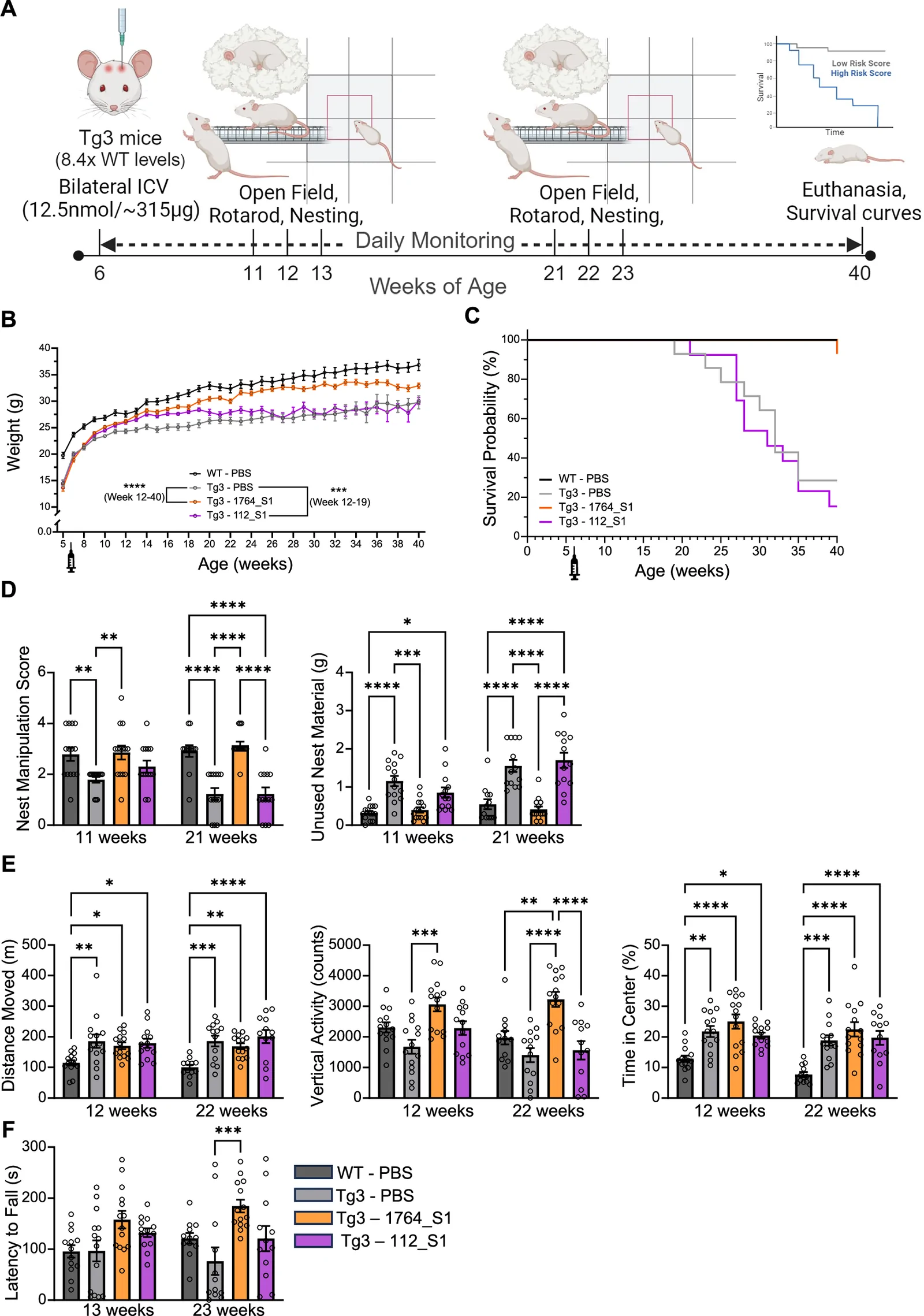
112_S1 partially and 1764_S1 completely rescues severe MDS in Tg3 mice. **A** Illustration of study design including dosage, route of administration, timeline and readouts in WT and Tg3 mice treated with PBS (black data points – WT, gray data points – Tg3), 1764_S1 (orange data points) and 112_S1 (purple data points). **B** Weight of treated animals over time (*n* = 14 mice per group). **C** Mortality of treated animals over time expressed as the probability of survival. **D** Nest building assay scores, including nest manipulation score and unused nesting material measured at 11 and 21 weeks of age (*n* = 13 mice for Tg3-112_S1 at 11 weeks, *n* = 14 for all other groups at 11 weeks; *n* = 12 mice for WT-PBS, *n* = 13 mice for Tg3-PBS and Tg3-112_S1, *n* = 14 mice for all other groups at 21 weeks). **E** Open field test results including distance moved (left), vertical activity counts (middle), and time in the center measured at 12 and 22 weeks of age (*n* = 13 mice for Tg3-112_S1 at 12 weeks, *n* = 14 for all other groups at 12 weeks; *n* = 12 mice for WT-PBS, *n* = 13 mice for Tg3-PBS and Tg3-112_S1, *n* = 14 mice for all other groups at 22 weeks). (**F**) Latency to fall on the rotarod test measured at 13 and 23 weeks of age (*n* = 13 mice for Tg3-112_S1 at 13 weeks, *n* = 14 for all other groups at 13 weeks; *n* = 12 mice for WT-PBS, *n* = 13 mice for Tg3-PBS and Tg3-112_S1, *n* = 14 mice for all other groups at 23 weeks). Data represented as mean ± s.e.m. of individual animals. Statistical analysis was performed using two-way ANOVA with adjustment for multiple comparisons for body weights, Log-rank for humane endpoint, and mixed effect model for nest building; *-*p* < 0.05, **-*p* < 0.01, ***-*p* < 0.001, ****-*p* < 0.0001). Exact *p*-values have been provided in the statistical analysis tables in the supplementary Table S3. A was created in BioRender. Fakih, H. (2026) https://BioRender.com/srdyn7n.

Adult six-week-old male Tg3 mice were administered 12.5 nmol ( ~ 315 µg) of either 1764_S1, 112_S1, or phosphate-buffered saline (PBS) as a vehicle control via intracerebroventricular (ICV) injection. This dose was chosen based on previously published data on divalent siRNAs with similar potency^8,46–48^. Wild-type littermates received PBS as a negative control for the MDS genotype. Technicians were blinded to the contents of each test article throughout the study. The mice were monitored for up to 40 weeks post-administration before euthanasia. Behavioral assessments included nest building at 11 and 21 weeks of age, the Open Field Test at 12 and 22 weeks of age, and rotarod performance at 13 and 23 weeks of age (Fig. 4A). These time points were chosen to evaluate behavior at a reasonable onset age and to track disease progression. The study aimed to assess the potential of lead siRNAs to alter behavior and mortality in MDS mice; tissue samples were not collected for molecular biology analyses before euthanasia.

Tg3 male mice started at lower body weights than WT males. However, both the non-isoform-selective 1764_S1 and the E1 isoform-selective 112_S1 increased the body weight of Tg3 mice to WT levels, with statistically significant weight differences from PBS-treated Tg3 mice observed between 12–19 weeks of age for 112_S1-treated Tg3 mice (*p* < 0.001). After 19 weeks of age, 112_S1-treated mice gained weight at a slower rate, aligning with Tg3 PBS levels thereafter. In contrast, 1764_S1-treated Tg3 mice showed significant weight improvement (*p* < 0.0001), maintaining this gain throughout their lifespan (Fig. 4B).

Tg3 mice exhibited early mortality, with a median survival probability of 32 weeks. Treatment with the E1 isoform-selective 112_S1 did not affect early mortality, resulting in a median survival probability of 31 weeks. Notably, 1764_S1-treated mice did not exhibit any signs of mortality up to 40 weeks of age, the study’s endpoint, showing survival indistinguishable from WT PBS mice (Fig. 4C).

The nest-building test, conducted at 11 and 21 weeks of age, was significantly impacted by genotype, with Tg3 PBS males showing lower nest scores and higher amounts of unused nest material compared to WT PBS males at both time points. Treatment with the E1 isoform-selective 112_S1 slightly improved nest scores and reduced unused nest material at 11 weeks compared to Tg3 PBS mice, but these improvements were not statistically significant and were not sustained at 21 weeks of age. In contrast, Tg3 mice treated with 1764_S1 showed a complete rescue of nest scores and unused nest material at 11 weeks of age, maintaining these improvements at 21 weeks of age, with scores closely resembling those of WT PBS mice (Fig. 4D).

The Open Field Test, administered at 12 and 22 weeks of age, revealed genotype effects, with Tg3 PBS males exhibiting increased distance moved, reduced vertical activity, and increased time spent in the center compared to WT PBS males. Neither 1764_S1 nor 112_S1 treatments rescued the increased distance moved or time spent in the center, as all Tg3 cohorts displayed similar patterns regardless of treatment. Although 112_S1 treatment slightly increased vertical activity to WT levels at 12 weeks of age, this effect was not statistically significant, and by 22 weeks of age, vertical activity in 112_S1-treated Tg3 mice aligned with Tg3 PBS controls. However, 1764_S1 treatment significantly increased vertical activity at both 12 weeks of age (*p* < 0.001) and 22 weeks of age (*p* < 0.0001) compared to Tg3 PBS controls, with activity levels even surpassing those of WT PBS males, indicating a potential overcorrection of this behavior (Fig. 4E).

The rotarod test, administered at 13 and 23 weeks of age, showed no genotype effects at 13 weeks, with similar latency to fall times for Tg3 PBS and WT PBS males. However, by 23 weeks of age, Tg3 PBS animals exhibited a reduced latency to fall compared to WT animals. Treatment with 112_S1 improved latency to fall in Tg3 animals at both 13 and 23 weeks, although this improvement was not statistically significant. In contrast, 1764_S1 treatment significantly increased latency to fall at 13 weeks of age (*p* < 0.05) and 23 weeks of age (*p* < 0.001) compared to Tg3 PBS males, with latency times even surpassing those of WT PBS males (Fig. 4F).

It is noteworthy that, despite the early mortality observed in Tg3 PBS males, the survival probability of this genotype was significantly higher, notwithstanding higher MECP2 transgene expression than in the original study describing these mice, where premature death was seen as early as 2–4 weeks of age^37^. This suggests that the MDS phenotype in Tg3 mice has been significantly modified since the transgenic line was first studied.

In conclusion, total *Mecp2* modulation through a single treatment with 1764_ S1 led to some neurobehavioral phenotype recovery and rescued the premature mortality observed in both control and isoform-selective treatment groups. Selective modulation of *Mecp2 E1* improved body weight initially, but the effects diminished after four months without impacting overall survival. It is unclear whether the lower disease-modifying efficacy of 112_S1 is due to the isoform specificity or due to differences in potency between 1764_S1 and 112_S1 in disease mice. Future studies would benefit from measuring the extent of gene silencing in the context of Tg3 mice to accurately correlate the pharmacodynamic effect of siRNA with behavioral modification. While this study provides proof-of-concept for disease modification, the amount of target gene silencing required to provide disease modification remains an outstanding question. Future studies using a repeat dosing regimen with target gene silencing as an additional readout are needed to evaluate the extent of gene silencing required and if isoform E1 silencing alone could be sufficient to ameliorate MDS phenotypes and provide long term disease modification.

## DISCUSSION

The development of nucleic acid therapeutics for treating rare genetic diseases holds immense promise, offering potential cures for conditions that currently rely solely on symptomatic management. However, addressing diseases involving dosage-sensitive genes such as *Mecp2* presents significant challenges, as both over-correction and under-correction can lead to pathological outcomes. In the case of MDS, excessive silencing of MECP2 may induce Rett-like syndromes, which are equally debilitating^2^. Therefore, the preclinical development of nucleic acid therapeutics for MDS must ensure that *Mecp2* silencing is robust, tunable, and durable to achieve therapeutic efficacy without adverse effects. One innovative approach we employed involves isoform-selective silencing, leveraging the functional overlap between the two *Mecp2* isoforms, E1 and E2, where one isoform can potentially compensate for the loss of the other^23^. We developed small interfering RNAs (siRNAs) that target all isoforms of *Mecp2* non-selectively, as well as siRNAs that selectively target the E1 isoform with minimal impact on E2, to identify the best therapeutic strategy.

A single dose of lead siRNAs achieved up to 80% *mecp2* mRNA silencing at four months post-injection in wildtype mice, with maximum silencing levels varying by isoform selectivity and chemical modifications. In the severe MDS mouse model (Tg3), a non-isoform selective siRNA (1764_S1) rescued weight loss, select behavioral deficits, and survival for the one-year study period, marking the first observed survival benefit in MDS therapy with a single dose. E1 isoform-selective silencing using 112_S1 provided initial gains in weight only until 19 weeks, despite multi-month durability in wildtype mice. This suggests that single-dose E1-selective silencing may not be as effective as total isoform silencing as a single dose. However, repeat dosing to maintain efficacy should be explored in future studies.

A major challenge in this study was the delayed onset of phenotypes in the Tg3 MDS models compared to published data. In this study, the median survival was 31 weeks, with the first death post-15 weeks, whereas literature reports mortality as early as 2–4 weeks^37^. While we were unable to investigate the cause of this transgenerational phenotypic variation, a variety of factors including epistasis, compensatory mechanisms and environmental factors could be contributing to the observed dampening of MDS phenotypes in these transgenic lines^49–51^.

Consistent with published reports, silencing durability varied by siRNA scaffold modifications, highlighting potential for tunable silencing in dosage-sensitive genes and pediatric cases^32,52^. A remarkable finding was the profound survival benefit and enduring phenotypic improvements observed for up to forty weeks following a single administration of the 1764_S1 lead compound at six weeks of age. The study’s complexity precluded tissue collection at survival endpoints, resulting in a lack of information regarding the exact duration of effect. However, previously published reports using divalent siRNA and ICV delivery in the mouse brain have demonstrated at least six months of pharmacodynamic activity for similar doses^8^. Given that the siRNA scaffold and not the sequence determines durability and pharmacokinetics, it is reasonable to conclude that the duration of silencing in this experiment was at least six months. However, due to the absence of silencing information, it remains uncertain whether the phenotypic benefits of 1764_S1 persisted beyond the pharmacodynamic effect as reported with antisense oligonucleotides in MDS^5^. Future studies need to evaluate gene silencing in disease models for a better understanding of the duration of the effect.

An important caveat to the behavioral experiments conducted in this study is the usage of a vehicle control (PBS) instead of the conventional non-targeting control (NTC) siRNA of similar backbone chemistry but scrambled sequence. The choice of PBS control prevents the evaluation of the chemical and structural effects of the siRNA backbone alone on the behavior of MDS mice. Since the current study is a pharmacology and proof-of-concept study intended to support further pre-clinical and clinical development, we elected to use a PBS control to evaluate the lead compounds ‘as a whole’, including both sequence-related (on-target) and chemical composition-related (off-target) effects to mimic the study design typically used in pre-clinical drug development and in clinical trials. Ideally, utilizing both a PBS and NTC control would allow for assessing the impact of sequence-related and chemical composition-related effects, but mouse model availability and practical cohort size limitations precluded this option. However, we have extensive historical control data across multiple mouse models (ALS, Alzheimer’s, and Huntington’s mouse models) showing that the divalent siRNA backbone and chemical structure of NTC do not affect disease^46,48,53^.

The siRNAs reported here are promising due to their extended duration of effect with a single administration. Since nucleic acid therapeutics generally cannot cross the blood-brain barrier (BBB) without a BBB shuttle or specific scaffold^54–57^, intrathecal (IT) or intracerebroventricular (ICV) administration is required, posing significant patient risks^11–14^. Therefore, durability is crucial for clinical utility and acceptance, particularly in the pediatric population. Additionally, divalent siRNAs show homogenous distribution and silencing in deep brain regions across multiple preclinical species^58–60^, making them ideal investigational therapeutic candidates for MDS, which affects all brain regions.

The current clinical landscape for MDS is led by ION440, an antisense oligonucleotide (ASO) therapeutic in Phase 1–2 trials. While experimental differences preclude a direct comparison between ION440 and the lead siRNA candidates presented here, these modalities offer complementary advantages. The catalytic nature of the RNA-induced silencing complex utilized by siRNAs allows for robust potency, a characteristic highlighted in previous comparative studies of oligonucleotide therapeutics^48^. ASOs, meanwhile, benefit from a longer history of clinical investigation, a substantial safety database, and dual-compartment activity in both the nucleus and cytoplasm^61^.

Clinical translation of these novel siRNAs will require further characterization of isoform-selective vs. non-selective silencing effects on the brain’s transcriptomic landscape, with comparisons to MECP2 knockout and overexpression controls via RNAseq. Preclinical toxicity studies must establish clear no adverse effect levels (NOAELs) in multiple species, ensuring safety at clinically effective doses. While MECP2 loss is linked to Rett syndrome, mainly studied in knockout models, these siRNAs will help determine the MECP2 reduction required to trigger Rett-like phenotypes postnatally and the timing between transcriptomic dysregulation and behavioral onset. Our wildtype mouse studies saw no overt effects from ~80% *mecp2* mRNA silencing for four months, though early behavioral changes may have been missed. Addressing these questions is likely essential for clinical use. Additionally, plasma biomarkers for target engagement need to be established, with MDS biomarker research from ongoing Ionis Pharmaceuticals trials potentially informing future programs.

This study has developed and preliminarily characterized siRNA compounds for MDS, paving the way for formal preclinical development. These siRNA tools also offer valuable resources for studying MECP2 isoform function and regulation, supporting sustained gene-silencing in Rett syndrome models across species. Future work will entail detailed molecular analyses of transcriptomic effects from total and E1 isoform-selective suppression and dose-ranging studies to clarify the pharmacokinetics and pharmacodynamics for safely regulating MECP2 levels.

## Materials And Methods

### siRNA design and selection

siRNA sequences were selected based on established rules of thermodynamics, G + C content, self-complementarity, and off-targets with seed region homology, using an algorithm developed by Shmushkovich et al^40^. Briefly, siRNAs were designed based on a 20-nucleotide target sequence from human (accession number: NM_001110792.2) and mouse (accession number: NM_001081979.2) MECP2 transcripts. siRNA sequences were excluded if they had (a) a high GC content ( > 56%), (b) single nucleotide stretches ( > 4), or (c) perfect homology to human miRNA seeds at the 2–7 position of the antisense strand. To minimize transcriptomic off-target effects, siRNAs were excluded if position 2–17 of the antisense strand had full complementarity to non-target mRNAs (except other MECP2/mecp2 isoforms). Targeting sequences (along with +10/ − 15 flanking nucleotides) were scored using a weight matrix, and top-scoring sequences in each species were identified^40^. Potential siRNA sequences were further filtered based on isoform selectivity, seed region matches to off-target mRNA and lncRNA, and species cross-homology. siRNAs were named based on the position in the target transcript from which they were extracted (e.g., human siRNA 1764 was extracted from positions 1764–1783 of NM_001110792.2); for cross-species targeting sequences, naming was based on the human transcript. Oligonucleotide synthesis, deprotection, purification for in vitro and in vivo experiments and LC-MS analysis are included in the Supplementary Materials.

### Cell culture

HeLa cells (ATCC, #CCL-2) and N2A cells (ATCC, #CCL-131) were maintained in Dulbecco′s Modified Eagle′s Medium (DMEM) (Cellgro, #10-013CV). The media were supplemented with 9% fetal bovine serum (FBS) (Gibco, #26140), and cells were grown at 37 °C and 5% CO2. All cells were maintained at 20–40% confluency and split every 3–5 days until the passage number reached 15. The supernatant medium was discarded, the cells were washed with DPBS (Gibco) and then detached by incubation with TrypLE Express (Gibco) for 5 min at 37 °C and 5% CO2. Trypsinization was halted by adding fresh media. Cell counting was performed by trypan blue staining using a Cellometer Auto 1000 instrument (Nexcelom).

### In vitro screening

HeLa and N2a cells were treated with cholesterol-conjugated siRNAs at 1.5 μM, which is the maximal dose for dose–response assays, for 72 h in 3% FBS media (50/50 volume of 6% FBS media/Opti-MEM media (Gibco, #31985–079)). Harvesting of cells was in a diluted lysis mixture consisting of a 1:2 ratio of lysis mixture (Invitrogen, #13228) to water and 0.2 mg/mL of proteinase K (Invitrogen, #25530–049), then lysed at 55 °C for 30 min incubation. For each compound, three technical replicates were prepared and incubated at 37 °C and 5% CO2. After 72 h, mRNA was quantified as described below. All experiments were performed in three independent biological replicates.

### Animal studies

All experimental studies involving mice were approved by the University of Massachusetts Chan Medical School Institutional Animal Care and Use Committee (IACUC) (protocol # A-2411) and the JAX In Vivo Pharmacology Services IACUC (protocol AUS #21053). FVB-Tg(MECP2)3Hzo/J (Tg3) (JR#8680) mice were obtained from the Jackson laboratory and maintained on the FVB background. Mice were housed at up to five mice per cage (University of Massachusetts Chan Medical School) or 3 mice per cage (JAX In Vivo Pharmacology Services) with food and water ad libitum.

Detailed procedures, including surgical administration of test articles and behavioral tests, are included in the Supplementary Materials.

### mRNA quantification

mRNA quantification was performed from in vitro cell lysate or from tissue punches stabilized in RNAlater (Invitrogen) using the QuantiGene™ Singleplex branched DNA assay (Invitrogen) as previously described^62^. Briefly, cells were lysed in QuantiGene™ Lysis Mixture and tissue biopsy punches were homogenized in QuantiGene™ Homogenizing Solution containing 0.2 mg/mL Proteinase K according to the manufacturer’s instructions. mRNA was detected according to the QuantiGene™ Singleplex branched DNA assay protocol using the following probe sets: mouse HPRT (SB-15463), mouse MECP2 (SB-11697), human HPRT (SA-10030), human MECP2 (SA-14665). Custom probe sets were designed to selectively detect mouse MECP2 E1 (DR7DPC3), mouse MECP2 E2 (DR47VRG), human MECP2 E1 (DR32Z69) and human MECP2 E2 (DR2W7MC). Luminescence was detected on a Tecan M1000 (Tecan) plate reader. In vitro experiments were performed using at least three independent biological replicates, and animal experiments were performed with at least four animals per group. The assay was run in technical duplicates.

### Microscopy and immunofluorescence of tissue sections

Formalin-fixed, paraffin-embedded tissue sections were deparaffinized in xylene, rehydrated, and stained with MECP2 (D43) XP Rabbit mAb #3456 at a 1:50 dilution (Cell Signaling Technology). Donkey anti-rabbit IgG (H + L) (Alexa Fluor® 594) (#A-21207, Invitrogen) was used as a secondary antibody at a 1:500 dilution. The nucleus was stained with DAPI (Molecular Probes). Images of three sections per mouse, 100 µm apart, were acquired with a Leica DMi8 inverted tiling microscope (Leica Microsystems) and processed using LAS-X.

### Image analysis and quantification of nuclei

Image analysis of immunofluorescence tissue sections was performed using the ImageJ tools. Briefly, images were loaded onto ImageJ and split into a DAPI channel and an MECP2 channel. The threshold for the DAPI signal was adjusted using the ‘adjust → threshold’ options until the DAPI signal was accurately partitioned from the background. After threshold adjustment, the particle analysis function was used by going to ‘analyze → analyze particles’ to obtain a region of interest (ROI) list. Each ROI was a nucleus identified by the ImageJ software. The ROI list was saved as ‘total nuclei’. Next, the MECP2 channel was preprocessed by background subtraction using a rolling ball radius of 50 pixels.

A custom ImageJ script was run to measure the MECP2 signal per nucleus that was previously identified from the DAPI channel and is available in supplementary methods.

### Reporting summary

Further information on research design is available in the Nature Portfolio Reporting Summary linked to this article.

## Supplementary information

Supplementary Information

Reporting Summary

Transparent Peer Review file

## Source data

Source Data

## Data Availability

All data are available in the main text or the supplementary information. Source data are provided with this paper.
