# Supplementary material for: Single-dose administration of therapeutic divalent siRNA targeting MECP2 prevents lethality in an MECP2 duplication mouse model
Source: Nat Commun. 2026 Jul 21;17:8877. doi: 10.1038/s41467-026-75611-7 (PMC13500697; doi:10.1038/s41467-026-75611-7)
Supplement: Supplementary file 1 [file 41467_2026_75611_MOESM1_ESM.pdf]

Supplementary Information

Single-dose administration of therapeutic divalent siRNA targeting MECP2 prevents lethality in an MECP2 duplication mouse model

Vignesh N. Hariharan[1]*, Ashley Summers[1], Amy E. Clipperton-Allen[2], Jillian Caiazzi[1], Samuel R. Hildebrand[1], Daniel O' Reilly[1], Qi Tang[1], Zachary Kennedy[1], Dimas Echeverria[1], Nicholas McHugh[1], David Cooper[1], Jacqueline Souza[1], Chantal Ferguson[1], Hassan H. Fakih[1], Laurent Bogdanik[2], Monica Coenraads[3] and Anastasia Khvorova[1]*

Affiliations:

[1]RNA Therapeutic Institute, University of Massachusetts Chan Medical School; Worcester, United States of America.
[2]The Jackson Laboratory; Bar Harbor, United States of America.
[3]The Rett Syndrome Research Trust; Trumbull, United States of America.

*Correspondence should be addressed to Vignesh.hariharan@umassmed.edu and Anastasia.khvorova@umassmed.edu.

Table of Contents

Supplementary Materials and Methods………………………………………………………...2
Supplementary Figures……………………………………………………………………………8
Supplementary Tables……………………………………………………………………………11
Source Data – Wes………..……………………………………………………………………...42

Supplementary Materials and Methods
Oligonucleotide synthesis

Oligonucleotides were synthesized by phosphoramidite solid-phase synthesis on automated synthesizer using a MerMade12 (Biosearch Technologies, Novato, CA), Dr Oligo 48 (Biolytic, Fremont, CA) or AKTA Oligopilot 100 (Cytiva, Marlborough, MA). 5'-(E)-Vinyl tetraphosphonate (pivaloyloxymethyl) 2'-O-methyl-uridine 3'-CE phosphoramidite was used for the addition of 5'-Vinyl Phosphonate, 2′-F, 2′-OMe phosphoramidites with standard protecting groups were used to make the modified oligonucleotides. Phosphoramidites were dissolved at 0.1 M in anhydrous acetonitrile (ACN), with added anhydrous 15% dimethylformamide in the case of the 2'-OMe-Uridine amidite. 5-(Benzylthio)-1H-tetrazole (BTT) was used as the activator at 0.25 M. Coupling times were 4 minutes. Detritylations was performed using 3% trichloroacetic acid in dichloromethane or Toluene. Capping reagents used were CAP A (20% N-methylimidazole in ACN) and CAP B (20% acetic anhydride and 30% 2,6-lutidine in ACN). Phosphite oxidation to convert to phosphate or phosphorothioate was performed with 0.05 M iodine in pyridine-water (9:1, v/v) or 0.1 M solution of 3-[(dimethylaminomethylene)amino]-3H-1,2,4-dithiazole-5-thione (DDTT) in pyridine for 3 min, respectively. All reagents were purchased from Chemgenes, Wilmington, MA, phospohoramidites were purchased from Chemgenes and Hongene Biotech, Union City, CA. Oligonucleotides were grown on long-chain alkyl amine (LCAA) 500Å controlled pore glass (CPG) functionalized with Unylinker terminus for unconjugated oligonucleotides. Cholesterol conjugated oligonucleotides were synthesized on a 500Å LCAA-CPG support, tetraethyleneglycol cholesterol moiety was the functionalized group joined by a succinate linker to the support (Chemgenes). Divalent oligonucleotides were synthesized on a 1000Å LCAA-CPG support functionalized via succinyl linker with a glycerol-tetraethyleneglycol linker (Hongene Biotech).

Deprotection and purification of oligonucleotides for in vitro experiments

Oligonucleotides with or without cholesterol conjugate were cleaved and deprotected on-column with Ammonia gas (Airgas Specialty Gases). Briefly, columns were pre-wet with 100uL of water and immediately spun to remove the excess water. Columns were then placed in a reaction chamber (Biolytic) 90min at 65°C. A modified on-column ethanol precipitation protocol was used for desalting and counterion exchange. Briefly, 1mL of 0.1M sodium acetate in 80% ethanol is flushed through the column, followed by a rinse with 1mL 80% ethanol and finally after drying the excess ethanol, oligonucleotides were eluted with 600uL of water in 96 deep well plates.

Deprotection and Purification of Oligonucleotides for in vivo Experiments

5'-(E)-Vinyl-phosphonate containing oligonucleotides were cleaved and deprotected with 3% diethylamine in ammonium hydroxide, for 20 h at 35°C with agitation. Divalent oligonucleotides were cleaved and deprotected with 1:1 ammonium hydroxide and 40% aqueous monomethylamine, for 2 h at 25°C with slight agitation. The controlled pore glass was subsequently filtered and rinsed with 30 mL of 5% ACN in water and dried overnight by centrifugal vacuum concentration. Purifications were performed on an Agilent 1290 Infinity II HPLC system using Source 15Q anion exchange resin (Cytiva). The loading solution was 20 mM sodium acetate in 10% ACN in water, and elution solution was the loading solution with 1M sodium bromide. Both oligonucleotide strands were eluted using a linear gradient from 30 to

70% in 40 min at 50°C. Peaks were monitored at 260nm. Pure fractions were combined and desalted by size exclusion using Sephadex G-25 resins (Cytiva). Oligonucleotides were then lyophilized and resuspended in water.

LC-MS analysis of oligonucleotides

Purity and identity of oligonucleotides were confirmed by IP-RP HPLC coupled to an Agilent 6530 Accurate-mass Q-TOF. LC parameters: buffer A: 100 mM 1,1,1,3,3,3-hexafluoroisopropanol (HFIP) (Oakwood Chemicals) and 9 mM triethylamine (TEA) (Fisher Scientific) in LC–MS grade water (Fisher Scientific); buffer B:100 mM HFIP and 9 mM TEA in LC–MS grade methanol (Fisher Scientific); column, Agilent AdvanceBio oligonucleotides C18; linear gradient 5–35% B 5min was used for unconjugated and divalent oligonucleotides; linear gradient 25–80% B 5min was used for cholesterol conjugated oligonucleotides; temperature, 60°C; flow rate, 0.85 ml/min. Peaks were monitored at 260nm. MS parameters: Source, electrospray ionization; ion polarity, negative mode; range, 100–3,200 m/z; scan rate, 2 spectra/s; capillary voltage, 4,000; fragmentor, 200 V; gas temp, 325°C.

Duplex formation

Equimolar amounts of antisense and sense strands were prepared in 1x PBS and incubated at 95°C for 5 minutes, cooled to room temperature and incubated overnight at 4°C. To validate efficient duplex formation, 2 pmol of duplex was loaded onto a non-denaturing 20% tris-borate-EDTA (TBE) gel (Invitrogen # EC63155BOX) and run at constant 180V for 1 hour. The gel was then washed with deionized water for 10 minutes, stained with 1X SYBR Gold Nucleic Acid Gel Stain (Invitrogen # S11494) for 10 minutes and washed again. Bands were visualized on the ChemiDoc MP Imaging system (Bio-Rad # 17001402).

ImageJ script for quantification of MECP2 in nuclei

```
//select directory to save output

 dir = getDirectory("Choose a Directory");
waitForUser("click on image you wish to analyze. make sure you have ROI set of nuclei already loaded. click ok to continue");

//calling ROI list of nuclei
n = roiManager('count');
for (i = 0; i < n; i++) {
roiManager('select', i);

//process roi here

run("Set Measurements...", "area mean modal min integrated median redirect=None decimal=3");
run("Measure");

//selectWindow("Summary");
}
selectWindow("Results");
```

```
Table.save(dir+File.separator+"results"+i+".csv"); //saves table
 waitForUser ("done, close Results table before next analysis.");
```

Animal Studies at JAX In Vivo Pharmacology Services

In-life Observations and Procedures

Mouse studies for the rescue of MDS in Tg3 mice.

All mice were males of strain FVB-Tg(MECP2)3Hzo/J (JAX #8680) and housed in the research animal facility at The Jackson Laboratory under standard conditions, including PIV caging and ad libitum food and water. The housing and testing rooms were lighted entirely with artificial fluorescent lighting, with a controlled 12 h light/dark cycle (6 am to 6 pm light). The normal temperature and relative humidity ranges in the animal rooms are 22 ± 4°C and 50 ± 15%, respectively. All procedures were approved by the Institutional Animal Care and Use Committee of The Jackson Laboratory in accordance with policies outlined in The Guide to the Care and Use of Laboratory Animals (Institute of Laboratory Animal Resources, National Research Council, National Academy of Sciences, 1996).

Animals were housed 3–5 per cage up until the first nest building assay at 11 weeks of age; at that age, all males were housed singly to prevent aggression, observed in previous studies following re-grouping mice. At least 7 days elapsed before the next behavioral test.

Behavioral tests were all performed during the light cycle.

Mice were humanely euthanized at the end of the testing period for tissue analyses, or earlier if they reached a humane endpoint. Humane endpoint was met upon review by a Veterinarian if an untreatable condition developed. Conditions observed in this study were paraphimosis, dehydration not responding to liquid gel supplementation in the cage, extreme agitation, sustained body weight loss, lameness or labored breathing. Age at humane endpoint was used for the survival analyses.

Complete Blinding of the study

Cage Cards, test article containers and Experimental Logs were blinded for treatment as “A” (Test article NTC), “B” (Test article M2), “C” (Test article M3), to blind the technicians performing injections, phenotypic scoring, body weight recording and behavioral tests for the dose injected. In addition, genotypes were also blinded.

Intracerebral Ventricular Injections (ICV) in Adult Mice

The mouse was anesthetized, and analgesia is administered.

The fur was removed, using clippers from the dorsal head in a roughly triangular area beginning ~ 3mm caudal to the nares and extending caudally to the cervical vertebrae. The lateral borders follow a line from the eye to the base of the pinna. Loose fur was removed with adhesive tape, dry gauze or gauze slightly dampened with ethanol. Ophthalmic ointment was placed on the eyes to prevent drying of the cornea. The skin was disinfected with a surgical scrub (iodine or chlorhexidine) and 70% ethanol using sterile swabs. Application of 70% ethanol starts in the center of the proposed incision site and works outward in ever widening circles to cover the entire clipped area. Using a new sterile swab, the surgical scrub was applied in the same manner. Repeat 70% ethanol and surgical scrub one additional time. The mouse was placed in ventral

recumbency with the head secured on a stereotaxic device. A 1cm midsagittal skin incision was made on the scalp to expose the skull. The underlying fascia was separated, and the skin was retracted laterally. With a sterile cotton-tipped applicator soaked in 3% hydrogen peroxide, the skull was cleared of tissue down to the bone. The site was flushed with saline. The sterile drill was attached to the manipulator arm of the stereotaxic unit and the tip of the drill bit was aligned directly over bregma, a landmark on the skull and a stereotaxic reference point. Bregma was the position on the skull where the coronal and sagittal sutures intersect. The surgeon sets the X and Y coordinates of the stereotaxic device to zero. All subsequent stereotaxic coordinates reference these zero points. The manipulator arm with the attached drill is positioned at M/L +/-1.0, A/P -.40, and depth of 2.6 for injection (depth varies from 2.4 to 2.8). A hole was drilled through the skull but leaving the dura intact. The diameter of the drill bur was generally 0.6-1.6 mm. The site is flushed with sterile saline. The drill was removed and replaced on the manipulator arm with the sterile injector. The injector was lowered through the hole in the skull to the predetermined Z point. The siRNA was slowly injected at the specified amounts in each experiment, and the injector held in place for ~1 minute. Mice received a bilateral injection of 5 ul per ventricle for a total of 10 ul per mouse. 0.1% bupivacaine was applied to edges of the skin incision. The skin was closed with 6-0 suture with a swaged-on needle or tissue adhesive. Absorbable suture may be used to close skin incisions, but it was not preferred as it causes a greater inflammatory response than non-absorbable suture. Silk, plain gut, or chromic gut suture must never be used to close skin incisions. All wound closure material, except tissue adhesive or absorbable suture, must be removed after the wound has healed.

Open Field Test

Subjects were acclimated to the testing room for at least 60 min.

Equipment: Omnitech Versamax Open Field Arenas (40cmx 40cm x 40cm) with photobeams.

Mice were placed individually into the center of the arena. Data were recorded via a sensitive infrared (IR) photobeam three-dimensional grid system that is invisible to mice. When the mouse moves or travels, its body breaks the otherwise continuous beam. The automated system then translates the beam breaks into measurements such as distance traveled (cm), horizontal activity, vertical activity (rearing), and time spent in the center (anxiety-like) for 60 min.

Rotarod

Subjects were acclimated to the testing room for a minimum of 60 min.

Equipment: Ugo-Basile Accelerating Rotarod for Mice (model 47600).

Up to 5 mice were placed on the rod, which was rotating at the baseline speed of 5 RPM. Once all mice were placed on the rod, it accelerated linearly to 40 RPM over a period of 300 sec (5 min). Mice are run on three successive trials, and the latency to fall, which is measured as the time from the initiation of the 300 sec linear acceleration period to when the mouse falls off the rod into the tray below, is recorded for each mouse on each trial. Mice lasting 300 sec on the rod, without falling, receive a “latency to fall” time of 300 sec.

Nest building

Nesting behavior is an intrinsic behavior performed by male and female rodents that requires fine motor skills. The nest building test is a simple and versatile behavioral test suitable for the

evaluation of motor deficits, cognitive decline as well as changes in general health or welfare. It is sensitive to environmental and physiological challenges, genetic mutations, and pharmacological interventions. There are several advantages of this test: it is performed in the home cage, without the presence of the experimenter, bedding material that is not used can be weighed to obtain an objective measure, and it is simple to perform while still yielding robust readouts. To test the individual nest building behavior, mice were individually housed in cages containing wood chip bedding and one square of pressed cotton `nestlet´(Ancare, Vatalog number NES3600). No other nesting material was provided. After 24hrs, photographs of the nests were taken, un-manipulated bedding material was weighed, and the manipulation of the nestlet and the quality of the built nest were analyzed manually according to a five-point scale.

Manipulation of the bedding material and nest quality were scored as follows on a rating scale modified from https://www.nature.com/articles/nprot.2006.170:

Manipulation of nestlet:

o Score of 0 = If the material is untouched

o Score of 1 = If it is not clear where the nest is located in the cage (i.e., the material is spread throughout the cage).

o Score of 2-5 (see below) = If nest is centralized to one spot

Nest site:

o Score of 2 = The nest is flat with no shallow walls, centralized in one spot

o Score of 3 = A slightly cupped shape where the walls of the nest are less than ½ the height of a dome that would cover a mouse.

o Score of 4 = Walls that are ½ the height of a dome that would cover a mouse.

o Score of 5 = Walls that are more than ½ the height of a dome that would cover a mouse, which may or may not fully enclose the nest.

Statistical Analyses

Statistical analyses were performed with GraphPad Prism v.10

For body weights, main effects of treatment, age, and treatment x age interaction were tested with a two-way ANOVA between the Tg3-PBS, Tg3-1764_S1 and Tg3-112_S1 groups. Multiplicity-adjusted p values were calculated with the Sidak test for each individual week, between the Tg3-PBS and Tg3-1764_S1 groups, and between the Tg3-PBS and Tg3-112_S1 groups. Figure 4.B represent the week span with significant differences (weeks 12-40 for Tg3-1764_S1 and weeks 12-19 for Tg3-112_S1).

For times to humane endpoint, ages in week of humane euthanasia for reaching endpoint, or of spontaneous death, were compared on a Kaplan-Meier curve with the Log-rank (Mantel-Cox) test between the Tg3-PBS, Tg3-1764_S1 and Tg3-112_S1 groups (Figure 4.C, $p<0.0001$).

Nest building scores, open field and wire hang performances at 12 and 22 weeks were analyzed with a mixed-effect model instead of a 2-way ANOVA to accommodate data points present at 12 but not 22 weeks. Main effects of the age and experimental groups, and age x group interaction were tested. Pairwise Tukey's multiple comparison tests for all possible comparisons at a given age were calculated and p value reported on the graphs in Figure 4.D-F.

Supplementary Figures

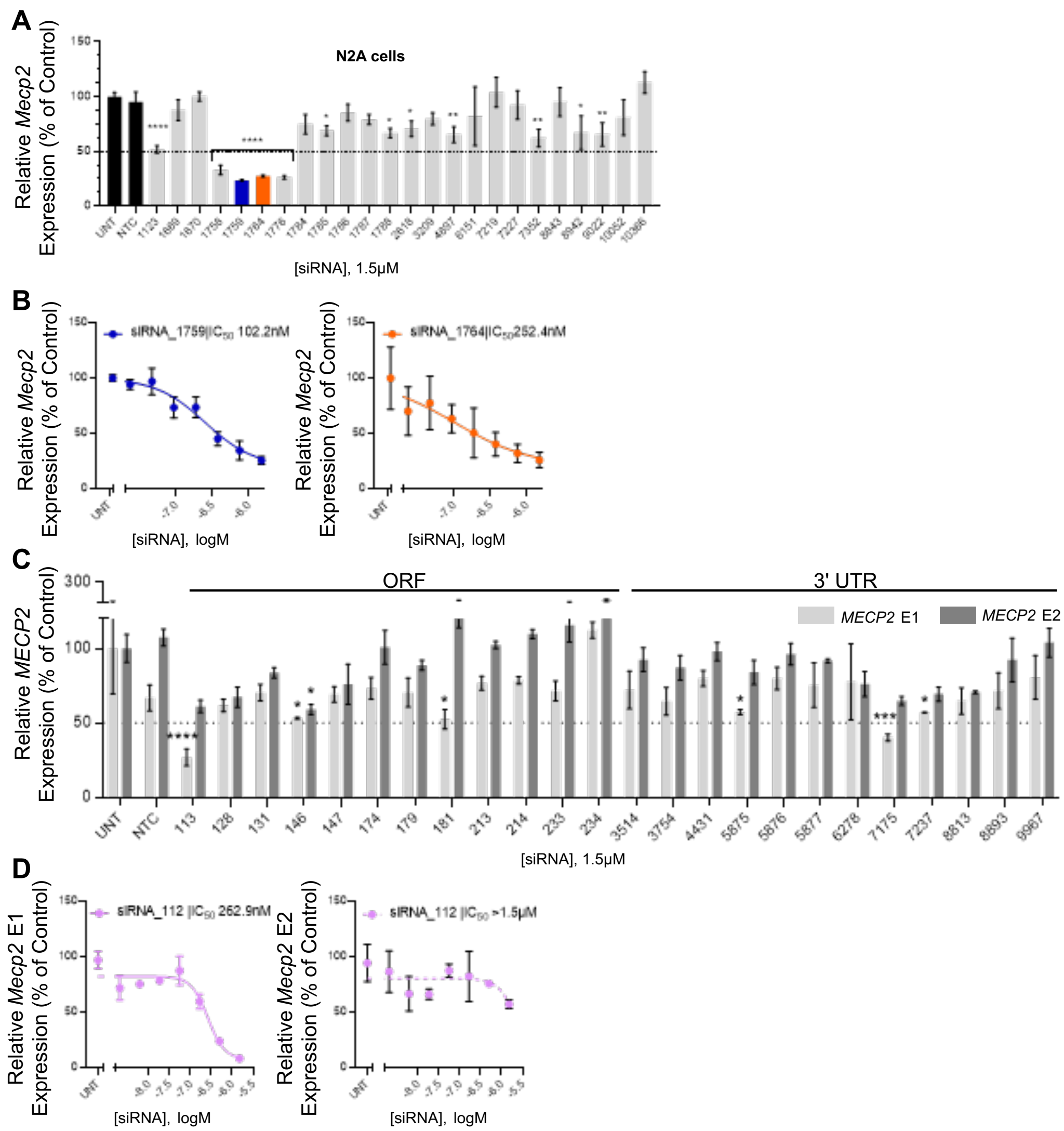

Supplementary Figure S1. **Lead siRNAs identified through in vitro screening**. (A) Relative *Mecp2* mRNA expression in mouse Neuro2A cells 72h post treatment with siRNAs at 1.5µM. (B) Relative *Mecp2* mRNA expression in mouse Neuro2A cells 72h post treatment with lead siRNAs in a seven-point dose response. (C) Relative *Mecp2* E1 (solid bars) and *Mecp2* E2 (patterned bars) mRNA expression in mouse Neuro2A cells 72h post treatment with siRNAs at 1.5µM. (D) Relative *Mecp2* E1 (left) and E2 (right) mRNA expression in mouse Neuro2A cells 72h post treatment with siRNA_112 in a seven-point dose response. Gene expression was measured using the QuantiGene™ Singleplex Assay Kit. Data represented as mean±s.e.m. of three independent replicates. Statistical analysis performed using Ordinary one-way ANOVA with Dunnett's adjustment for multiple comparisons (*-p<0.05, **-p<0.01, ***-p<0.001, ****-p<0.0001).

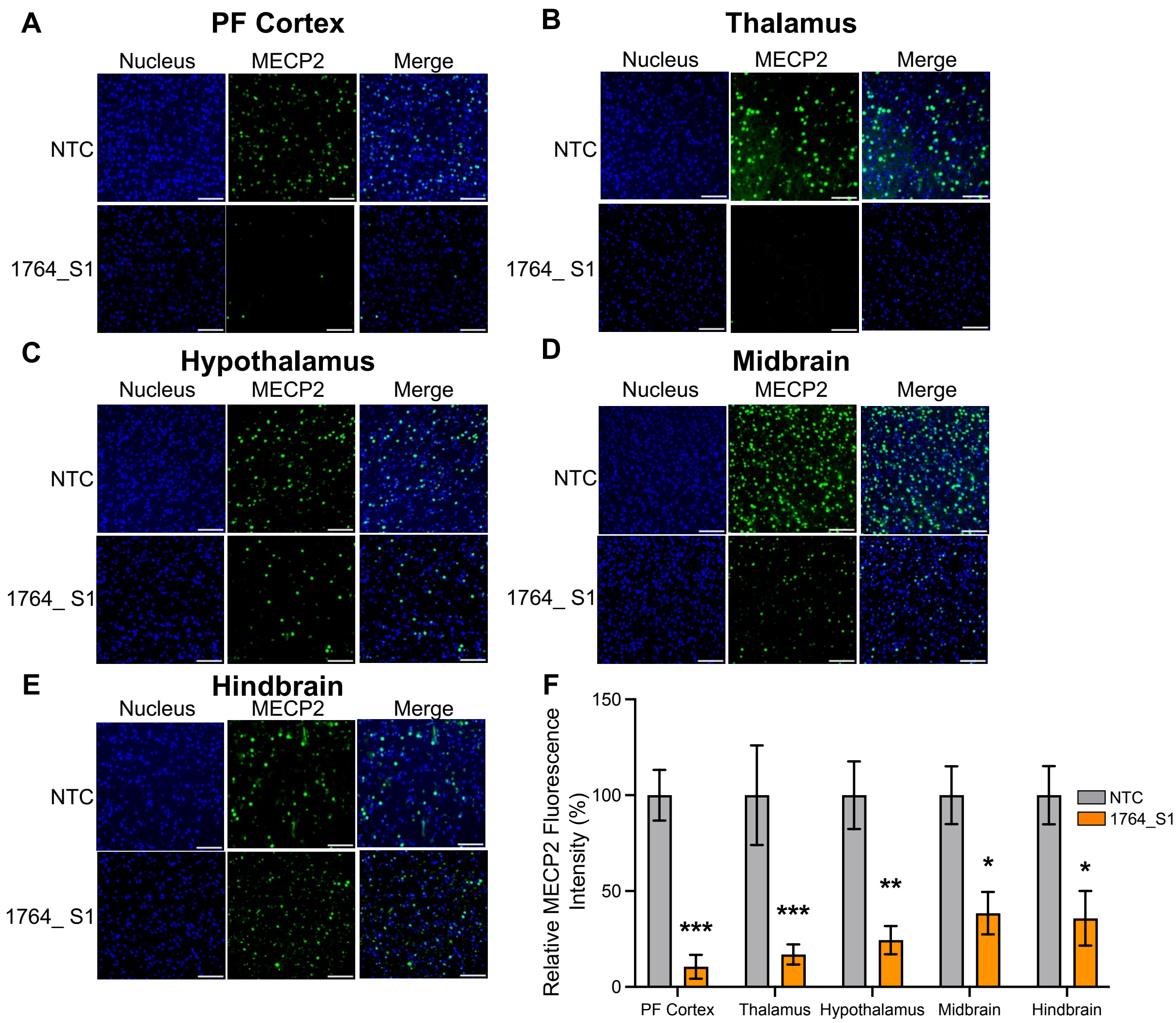

Supplementary Figure S2. Quantification of MECP2 positive nuclei in brain sections. Representative immunofluorescence images of DAPI and MECP2 stained sections of (A) prefrontal (PF) cortex, (B) thalamus, (C) hypothalamus, (D) midbrain, and (E) hindbrain along with (F) quantification of nuclear immunofluorescence intensity of MECP2. Data represented as mean±s.e.m. of individual animals. Immunofluorescence imaging was performed on sagittal brain sections stained with DAPI and anti-MECP2 antibody and imaged using Leica DMi8 widefield microscope. Quantification of MECP2 nuclear intensity was performed using a custom imageJ script. Statistical analysis performed using two-way ANOVA with Dunnett's adjustment for multiple comparisons (*-$p<0.05$, **-$p<0.01$, ***-$p<0.001$).

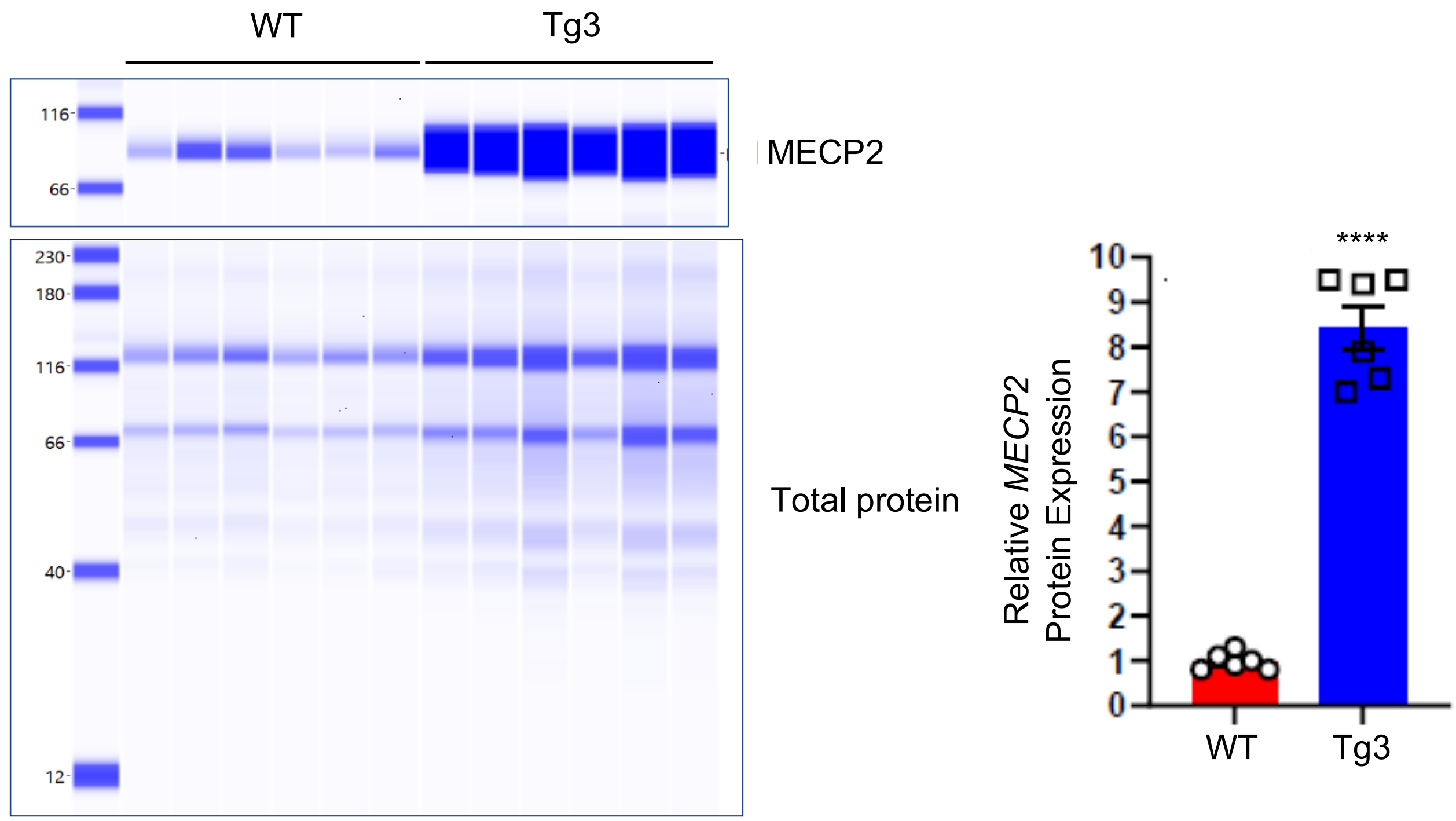

Supplementary Figure S3. Validation of Tg3 mouse model. Western blot gel images (left) and quantification of protein bands (right) from 4 weeks old Tg3 mice. Mecp2 protein bands were normalized to total protein. WT = FVB background, Tg3 = transgenic strain. Data in bar graphs represented as mean±s.d. of individual animals (n=6 mice per group). Statistical significance computed by unpaired t-test (****-p<0.0001).

Supplementary Table S1. List of oligonucleotide sequences used in this study

Single strand ID	Oligo Name	Modified sequence
7051	MECP2_575_s	(mG)#(mA)#(mA)(mG)(fU)(fA)(fU)(mG)(fA)(mU)(mG)(mU)(mG)#(mU)#(mA)-TegChol
7087	MECP2_575_as	P(mU)#(fA)#(mC)(mA)(mC)(fA)(mU)(mC)(mA)(mU)(mA)(mC)(mU)#(fU)#(mC)#(fC)#(mC)#(mA)#(mG)#(fC)
7052	MECP2_572_s	(mU)#(mG)#(mG)(mG)(fA)(fA)(fG)(mU)(fA)(mU)(mG)(mA)(mU)#(mG)#(mA)-TegChol
7088	MECP2_572_as	P(mU)#(fC)#(mA)(mU)(mC)(fA)(mU)(mA)(mC)(mU)(mU)(mC)(mC)#(fC)#(mA)#(fG)#(mC)#(mA)#(mG)#(fA)
7053	MECP2_300_s	(mA)#(mA)#(mG)(mU)(fU)(fU)(fA)(mA)(fA)(mA)(mA)(mG)(mG)#(mU)#(mA)-TegChol
7089	MECP2_300_as	P(mU)#(fA)#(mC)(mC)(mU)(fU)(mU)(mU)(mU)(mA)(mA)(mA)(mC)#(fU)#(mU)#(fG)#(mA)#(mG)#(mG)#(fG)
7054	MECP2_827_s	(mC)#(mA)#(mC)(mG)(fU)(fC)(fA)(mG)(fA)(mG)(mG)(mG)(mU)#(mG)#(mA)-TegChol
7090	MECP2_827_as	P(mU)#(fC)#(mA)(mC)(mC)(fC)(mU)(mC)(mU)(mG)(mA)(mC)(mG)#(fU)#(mG)#(fG)#(mC)#(mC)#(mG)#(fC)
7055	MECP2_603_s	(mC)#(mA)#(mG)(mG)(fG)(fA)(fA)(mA)(fA)(mG)(mC)(mC)(mU)#(mU)#(mA)-TegChol
7091	MECP2_603_as	P(mU)#(fA)#(mA)(mG)(mG)(fC)(mU)(mU)(mU)(mU)(mC)(mC)(mC)#(fU)#(mG)#(fG)#(mG)#(mG)#(mA)#(fU)
7056	MECP2_887_s	(mC)#(mA)#(mA)(mG)(fA)(fU)(fG)(mC)(fC)(mU)(mU)(mU)(mU)#(mC)#(mA)-TegChol
7092	MECP2_887_as	P(mU)#(fG)#(mA)(mA)(mA)(fA)(mG)(mG)(mC)(mA)(mU)(mC)(mU)#(fU)#(mG)#(fA)#(mC)#(mA)#(mA)#(fG)
7057	MECP2_285_s	(mA)#(mA)#(mG)(mG)(fA)(fC)(fA)(mA)(fA)(mC)(mC)(mC)(mC)#(mU)#(mA)-TegChol
7093	MECP2_285_as	P(mU)#(fA)#(mG)(mG)(mG)(fG)(mU)(mU)(mU)(mG)(mU)(mC)(mC)#(fU)#(mU)#(fG)#(mA)#(mG)#(mG)#(fC)
7058	MECP2_605_s	(mG)#(mG)#(mG)(mA)(fA)(fA)(fA)(mG)(fC)(mC)(mU)(mU)(mU)#(mC)#(mA)-TegChol
7094	MECP2_605_as	P(mU)#(fG)#(mA)(mA)(mA)(fG)(mG)(mC)(mU)(mU)(mU)(mU)(mC)#(fC)#(mC)#(fU)#(mG)#(mG)#(mG)#(fG)
7059	MECP2_662_s	(mC)#(mA)#(mC)(mA)(fU)(fC)(fC)(mC)(fU)(mG)(mG)(mA)(mC)#(mC)#(mA)-TegChol
7095	MECP2_662_as	P(mU)#(fG)#(mG)(mU)(mC)(fC)(mA)(mG)(mG)(mG)(mA)(mU)(mG)#(fU)#(mG)#(fU)#(mC)#(mG)#(mC)#(fC)

7060	MECP2_535_s	(mC)#(mA)#(mC)(mG)(fG)(fA)(fA)(mG)(fC)(mU)(mU)(mA)(mA)#(mG)#(mA)-TegChol
7096	MECP2_535_as	P(mU)#(fC)#(mU)(mU)(mA)(fA)(mG)(mC)(mU)(mU)(mC)(mC)(mG)#(fU)#(mG)#(fU)#(mC)#(mC)#(mA)#(fG)
7061	MECP2_303_s	(mU)#(mU)#(mU)(mA)(fA)(fA)(fA)(mA)(fG)(mG)(mU)(mG)(mA)#(mA)#(mA)-TegChol
7097	MECP2_303_as	P(mU)#(fU)#(mU)(mC)(mA)(fC)(mC)(mU)(mU)(mU)(mU)(mU)(mA)#(fA)#(mA)#(fC)#(mU)#(mU)#(mG)#(fA)
7062	MECP2_895_s	(mC)#(mU)#(mU)(mU)(fU)(fC)(fA)(mA)(fA)(mC)(mU)(mU)(mC)#(mG)#(mA)-TegChol
7098	MECP2_895_as	P(mU)#(fC)#(mG)(mA)(mA)(fG)(mU)(mU)(mU)(mG)(mA)(mA)(mA)#(fA)#(mG)#(fG)#(mC)#(mA)#(mU)#(fC)
7063	MECP2_1776_s	(mA)#(mU)#(mU)(mA)(fA)(fC)(fU)(mG)(fA)(mA)(mA)(mU)(mA)#(mA)#(mA)-TegChol
7099	MECP2_1776_as	P(mU)#(fU)#(mU)(mA)(mU)(fU)(mU)(mC)(mA)(mG)(mU)(mU)(mA)#(fA)#(mU)#(fC)#(mG)#(mG)#(mG)#(fA)
7064	MECP2_1785_s	(mA)#(mA)#(mU)(mA)(fA)(fA)(fA)(mA)(fA)(mU)(mA)(mU)(mU)#(mU)#(mA)-TegChol
7100	MECP2_1785_as	P(mU)#(fA)#(mA)(mA)(mU)(fA)(mU)(mU)(mU)(mU)(mU)(mU)(mA)#(fU)#(mU)#(fU)#(mC)#(mA)#(mG)#(fU)
7065	MECP2_1759_s	(mU)#(mC)#(mU)(mG)(fA)(fC)(fA)(mA)(fA)(mG)(mC)(mU)(mU)#(mC)#(mA)-TegChol
7101	MECP2_1759_as	P(mU)#(fG)#(mA)(mA)(mG)(fC)(mU)(mU)(mU)(mG)(mU)(mC)(mA)#(fG)#(mA)#(fG)#(mC)#(mC)#(mC)#(fU)
7066	Mecp2_1123_s	(mC)#(mA)#(mA)(mA)(fA)(fA)(fG)(mA)(fA)(mA)(mG)(mC)(mC)#(mG)#(mA)-TegChol
7102	Mecp2_1123_as	P(mU)#(fC)#(mG)(mG)(mC)(fU)(mU)(mU)(mC)(mU)(mU)(mU)(mU)#(fU)#(mG)#(fG)#(mC)#(mC)#(mU)#(fC)
7067	MECP2_1787_s	(mU)#(mA)#(mA)(mA)(fA)(fA)(fA)(mU)(fA)(mU)(mU)(mU)(mU)#(mU)#(mA)-TegChol
7103	MECP2_1787_as	P(mU)#(fA)#(mA)(mA)(mA)(fA)(mU)(mA)(mU)(mU)(mU)(mU)(mU)#(fU)#(mA)#(fU)#(mU)#(mU)#(mC)#(fA)
7068	MECP2_1784_s	(mA)#(mA)#(mA)(mU)(fA)(fA)(fA)(mA)(fA)(mA)(mU)(mA)(mU)#(mU)#(mA)-TegChol
7104	MECP2_1784_as	P(mU)#(fA)#(mA)(mU)(mA)(fU)(mU)(mU)(mU)(mU)(mU)(mA)(mU)#(fU)#(mU)#(fC)#(mA)#(mG)#(mU)#(fU)
7069	MECP2_1786_s	(mA)#(mU)#(mA)(mA)(fA)(fA)(fA)(mA)(fU)(mA)(mU)(mU)(mU)#(mU)#(mA)-TegChol

7105	MECP2_1786_as	P(mU)#(fA)#(mA)(mA)(mA)(fU)(mA)(mU)(mU)(mU)(mU)(mU)(mU)#(fA)#(mU)#(fU)#(mU)#(mC)#(mA)#(fG)
7070	MECP2_1788_s	(mA)#(mA)#(mA)(mA)(fA)(fA)(fU)(mA)(fU)(mU)(mU)(mU)(mU)#(mU)#(mA)-TegChol
7106	MECP2_1788_as	P(mU)#(fA)#(mA)(mA)(mA)(fA)(mA)(mU)(mA)(mU)(mU)(mU)(mU)#(fU)#(mU)#(fA)#(mU)#(mU)#(mU)#(fC)
7071	MECP2_1758_s	(mC)#(mU)#(mC)(mU)(fG)(fA)(fC)(mA)(fA)(mA)(mG)(mC)(mU)#(mU)#(mA)-TegChol
7107	MECP2_1758_as	P(mU)#(fA)#(mA)(mG)(mC)(fU)(mU)(mU)(mG)(mU)(mC)(mA)(mG)#(fA)#(mG)#(fC)#(mC)#(mC)#(mU)#(fA)
7072	MECP2_1669_s	(mA)#(mU)#(mA)(mU)(fU)(fU)(fU)(mU)(fU)(mU)(mU)(mU)(mC)#(mU)#(mA)-TegChol
7108	MECP2_1669_as	P(mU)#(fA)#(mG)(mA)(mA)(fA)(mA)(mA)(mA)(mA)(mA)(mA)(mU)#(fA)#(mU)#(fU)#(mU)#(mU)#(mU)#(fU)
7073	MECP2_1670_s	(mU)#(mA)#(mU)(mU)(fU)(fU)(fU)(mU)(fU)(mU)(mU)(mC)(mU)#(mU)#(mA)-TegChol
7109	MECP2_1670_as	P(mU)#(fA)#(mA)(mG)(mA)(fA)(mA)(mA)(mA)(mA)(mA)(mA)(mA)#(fU)#(mA)#(fU)#(mU)#(mU)#(mU)#(fU)
7074	MECP2_1764_s	(mC)#(mA)#(mA)(mA)(fG)(fC)(fU)(mU)(fC)(mC)(mC)(mG)(mA)#(mU)#(mA)-TegChol
7110	MECP2_1764_as	P(mU)#(fA)#(mU)(mC)(mG)(fG)(mG)(mA)(mA)(mG)(mC)(mU)(mU)#(fU)#(mG)#(fU)#(mC)#(mA)#(mG)#(fA)
7075	Mecp2_4897_s	(mC)#(mA)#(mU)(mU)(fG)(fU)(fA)(mA)(fA)(mG)(mU)(mG)(mU)#(mG)#(mA)-TegChol
7111	Mecp2_4897_as	P(mU)#(fC)#(mA)(mC)(mA)(fC)(mU)(mU)(mU)(mA)(mC)(mA)(mA)#(fU)#(mG)#(fU)#(mU)#(mC)#(mA)#(fA)
7076	Mecp2_7219_s	(mU)#(mA)#(mU)(mA)(fU)(fA)(fU)(mA)(fU)(mA)(mU)(mC)(mU)#(mG)#(mA)-TegChol
7112	Mecp2_7219_as	P(mU)#(fC)#(mA)(mG)(mA)(fU)(mA)(mU)(mA)(mU)(mA)(mU)(mA)#(fU)#(mA)#(fU)#(mA)#(mU)#(mA)#(fU)
7077	Mecp2_10052_s	(mU)#(mU)#(mU)(mG)(fG)(fG)(fA)(mC)(fA)(mA)(mU)(mU)(mA)#(mC)#(mA)-TegChol
7113	Mecp2_10052_as	P(mU)#(fG)#(mU)(mA)(mA)(fU)(mU)(mG)(mU)(mC)(mC)(mC)(mA)#(fA)#(mA)#(fG)#(mC)#(mA)#(mC)#(fA)
7078	Mecp2_6151_s	(mA)#(mA)#(mA)(mA)(fA)(fU)(fU)(mA)(fU)(mA)(mU)(mU)(mU)#(mU)#(mA)-TegChol
7114	Mecp2_6151_as	P(mU)#(fA)#(mA)(mA)(mA)(fU)(mA)(mU)(mA)(mA)(mU)(mU)(mU)#(fU)#(mU)#(fU)#(mG)#(mU)#(mU)#(fA)

7079	Mecp2_3209_s	(mC)#(mC)#(mU)(mU)(fA)(fA)(fA)(mC)(fA)(mA)(mU)(mG)(mA)#(mG)#(mA)-TegChol
7115	Mecp2_3209_as	P(mU)#(fC)#(mU)(mC)(mA)(fU)(mU)(mG)(mU)(mU)(mU)(mA)(mA)#(fG)#(mG)#(fU)#(mC)#(mU)#(mG)#(fA)
7080	Mecp2_2618_s	(mA)#(mA)#(mG)(mG)(fA)(fA)(fA)(mA)(fU)(mA)(mU)(mU)(mC)#(mU)#(mA)-TegChol
7116	Mecp2_2618_as	P(mU)#(fA)#(mG)(mA)(mA)(fU)(mA)(mU)(mU)(mU)(mU)(mC)(mC)#(fU)#(mU)#(fU)#(mC)#(mU)#(mU)#(fC)
7081	Mecp2_9022_s	(mC)#(mA)#(mG)(mA)(fG)(fA)(fC)(mA)(fA)(mA)(mU)(mG)(mC)#(mU)#(mA)-TegChol
7117	Mecp2_9022_as	P(mU)#(fA)#(mG)(mC)(mA)(fU)(mU)(mU)(mG)(mU)(mC)(mU)(mC)#(fU)#(mG)#(fG)#(mA)#(mA)#(mC)#(fA)
7082	Mecp2_7352_s	(mA)#(mA)#(mG)(mG)(fG)(fA)(fA)(mA)(fA)(mA)(mA)(mU)(mU)#(mU)#(mA)-TegChol
7118	Mecp2_7352_as	P(mU)#(fA)#(mA)(mA)(mU)(fU)(mU)(mU)(mU)(mU)(mC)(mC)(mC)#(fU)#(mU)#(fG)#(mU)#(mC)#(mC)#(fU)
7083	Mecp2_10366_s	(mC)#(mA)#(mU)(mA)(fA)(fG)(fG)(mU)(fU)(mC)(mU)(mU)(mU)#(mU)#(mA)-TegChol
7119	Mecp2_10366_as	P(mU)#(fA)#(mA)(mA)(mA)(fG)(mA)(mA)(mC)(mC)(mU)(mU)(mA)#(fU)#(mG)#(fA)#(mA)#(mA)#(mA)#(fA)
7084	Mecp2_8843_s	(mU)#(mU)#(mG)(mG)(fU)(fU)(fU)(mU)(fA)(mU)(mU)(mU)(mU)#(mU)#(mA)-TegChol
7120	Mecp2_8843_as	P(mU)#(fA)#(mA)(mA)(mA)(fA)(mU)(mA)(mA)(mA)(mA)(mC)(mC)#(fA)#(mA)#(fA)#(mC)#(mC)#(mA)#(fA)
7085	Mecp2_8942_s	(mG)#(mU)#(mG)(mA)(fA)(fA)(fG)(mG)(fA)(mA)(mC)(mU)(mU)#(mU)#(mA)-TegChol
7121	Mecp2_8942_as	P(mU)#(fA)#(mA)(mA)(mG)(fU)(mU)(mC)(mC)(mU)(mU)(mU)(mC)#(fA)#(mC)#(fC)#(mC)#(mA)#(mC)#(fC)
7086	Mecp2_7227_s	(mU)#(mA)#(mU)(mC)(fU)(fG)(fU)(mA)(fU)(mA)(mU)(mU)(mU)#(mC)#(mA)-TegChol
7122	Mecp2_7227_as	P(mU)#(fG)#(mA)(mA)(mA)(fU)(mA)(mU)(mA)(mC)(mA)(mG)(mA)#(fU)#(mA)#(fU)#(mA)#(mU)#(mA)#(fU)
8174	MECP2_E1_s_111	(mA)#(mG)#(fG)(mA)(fG)(mG)(fA)(mG)(fA)(mG)(mA)(mC)(fU)#(mG)#(mA)-TegChol
8222	MECP2_E1_as_111	P(mU)#(fC)#(mA)(fG)(fU)(fC)(mU)(fC)(mU)(fC)(mC)(fU)(mC)#(fC)#(mU)#(fC)#(mG)#(mC)#(mC)#(fU)
8168	MECP2_E1_s_112	(mG)#(mG)#(fA)(mG)(fG)(mA)(fG)(mA)(fG)(mA)(mC)(mU)(fG)#(mG)#(mA)-TegChol
8216	MECP2_E1_as_112	P(mU)#(fC)#(mC)(fA)(fG)(fU)(mC)(fU)(mC)(fU)(mC)(fC)(mU)#(fC)#(mC)#(fU)#(mC)#(mG)#(mC)#(fC)

8169	MECP2_E1_s_113	(mG)#(mA)#(fG)(mG)(fA)(mG)(fA)(mG)(fA)(mC)(mU)(mG)(fG)#(mA)#(mA)-TegChol
8217	MECP2_E1_as_113	P(mU)#(fU)#(mC)(fC)(fA)(fG)(mU)(fC)(mU)(fC)(mU)(fC)(mC)#(fU)#(mC)#(fC)#(mU)#(mC)#(mG)#(fC)
8170	MECP2_E1_s_114	(mA)#(mG)#(fG)(mA)(fG)(mA)(fG)(mA)(fC)(mU)(mG)(mG)(fA)#(mA)#(mA)-TegChol
8218	MECP2_E1_as_114	P(mU)#(fU)#(mU)(fC)(fC)(fA)(mG)(fU)(mC)(fU)(mC)(fU)(mC)#(fC)#(mU)#(fC)#(mC)#(mU)#(mC)#(fG)
8173	MECP2_E1_s_115	(mG)#(mG)#(fA)(mG)(fA)(mG)(fA)(mC)(fU)(mG)(mG)(mA)(fA)#(mG)#(mA)-TegChol
8221	MECP2_E1_as_115	P(mU)#(fC)#(mU)(fU)(fC)(fC)(mA)(fG)(mU)(fC)(mU)(fC)(mU)#(fC)#(mC)#(fU)#(mC)#(mC)#(mU)#(fC)
8176	MECP2_E1_s_117	(mA)#(mG)#(fA)(mG)(fA)(mC)(fU)(mG)(fG)(mA)(mA)(mG)(fA)#(mA)#(mA)-TegChol
8224	MECP2_E1_as_117	P(mU)#(fU)#(mU)(fC)(fU)(fU)(mC)(fC)(mA)(fG)(mU)(fC)(mU)#(fC)#(mU)#(fC)#(mC)#(mU)#(mC)#(fC)
8175	MECP2_E1_s_118	(mG)#(mA)#(fG)(mA)(fC)(mU)(fG)(mG)(fA)(mA)(mG)(mA)(fA)#(mA)#(mA)-TegChol
8223	MECP2_E1_as_118	P(mU)#(fU)#(mU)(fU)(fC)(fU)(mU)(fC)(mC)(fA)(mG)(fU)(mC)#(fU)#(mC)#(fU)#(mC)#(mC)#(mU)#(fC)
8171	MECP2_E1_s_121	(mA)#(mC)#(fU)(mG)(fG)(mA)(fA)(mG)(fA)(mA)(mA)(mA)(fG)#(mU)#(mA)-TegChol
8219	MECP2_E1_as_121	P(mU)#(fA)#(mC)(fU)(fU)(fU)(mU)(fC)(mU)(fU)(mC)(fC)(mA)#(fG)#(mU)#(fC)#(mU)#(mC)#(mU)#(fC)
8166	MECP2_E1_s_122	(mC)#(mU)#(fG)(mG)(fA)(mA)(fG)(mA)(fA)(mA)(mA)(mG)(fU)#(mC)#(mA)-TegChol
8214	MECP2_E1_as_122	P(mU)#(fG)#(mA)(fC)(fU)(fU)(mU)(fU)(mC)(fU)(mU)(fC)(mC)#(fA)#(mG)#(fU)#(mC)#(mU)#(mC)#(fU)
8177	MECP2_E1_s_123	(mU)#(mG)#(fG)(mA)(fA)(mG)(fA)(mA)(fA)(mA)(mG)(mU)(fC)#(mA)#(mA)-TegChol
8225	MECP2_E1_as_123	P(mU)#(fU)#(mG)(fA)(fC)(fU)(mU)(fU)(mU)(fC)(mU)(fU)(mC)#(fC)#(mA)#(fG)#(mU)#(mC)#(mU)#(fC)
8164	MECP2_E1_s_126	(mA)#(mA)#(fG)(mA)(fA)(mA)(fA)(mG)(fU)(mC)(mA)(mG)(fA)#(mA)#(mA)-TegChol
8212	MECP2_E1_as_126	P(mU)#(fU)#(mU)(fC)(fU)(fG)(mA)(fC)(mU)(fU)(mU)(fU)(mC)#(fU)#(mU)#(fC)#(mC)#(mA)#(mG)#(fU)
8172	MECP2_E1_s_127	(mA)#(mG)#(fA)(mA)(fA)(mA)(fG)(mU)(fC)(mA)(mG)(mA)(fA)#(mG)#(mA)-TegChol

8220	MECP2_E1_as_127	P(mU)#(fC)#(mU)(fU)(fC)(fU)(mG)(fA)(mC)(fU)(mU)(fU)(mU)#(fC)#(mU)#(fU)#(mC)#(mC)#(mA)#(fG)
8155	MECP2_E1_s_1678	(mU)#(mU)#(fU)(mC)(fU)(mU)(fU)(mC)(fA)(mG)(mU)(mA)(fA)#(mA)#(mA)-TegChol
8203	MECP2_E1_as_1678	P(mU)#(fU)#(mU)(fU)(fA)(fC)(mU)(fG)(mA)(fA)(mA)(fG)(mA)#(fA)#(mA)#(fA)#(mA)#(mA)#(mA)#(fA)
8167	MECP2_E1_s_1679	(mU)#(mU)#(fC)(mU)(fU)(mU)(fC)(mA)(fG)(mU)(mA)(mA)(fA)#(mA)#(mA)-TegChol
8215	MECP2_E1_as_1679	P(mU)#(fU)#(mU)(fU)(fU)(fA)(mC)(fU)(mG)(fA)(mA)(fA)(mG)#(fA)#(mA)#(fA)#(mA)#(mA)#(mA)#(fA)
8159	MECP2_E1_s_1680	(mU)#(mC)#(fU)(mU)(fU)(mC)(fA)(mG)(fU)(mA)(mA)(mA)(fA)#(mA)#(mA)-TegChol
8207	MECP2_E1_as_1680	P(mU)#(fU)#(mU)(fU)(fU)(fU)(mA)(fC)(mU)(fG)(mA)(fA)(mA)#(fG)#(mA)#(fA)#(mA)#(mA)#(mA)#(fA)
8162	MECP2_E1_s_1681	(mC)#(mU)#(fU)(mU)(fC)(mA)(fG)(mU)(fA)(mA)(mA)(mA)(fA)#(mA)#(mA)-TegChol
8210	MECP2_E1_as_1681	P(mU)#(fU)#(mU)(fU)(fU)(fU)(mU)(fA)(mC)(fU)(mG)(fA)(mA)#(fA)#(mG)#(fA)#(mA)#(mA)#(mA)#(fA)
8158	MECP2_E1_s_1682	(mU)#(mU)#(fU)(mC)(fA)(mG)(fU)(mA)(fA)(mA)(mA)(mA)(fA)#(mA)#(mA)-TegChol
8206	MECP2_E1_as_1682	P(mU)#(fU)#(mU)(fU)(fU)(fU)(mU)(fU)(mA)(fC)(mU)(fG)(mA)#(fA)#(mA)#(fG)#(mA)#(mA)#(mA)#(fA)
8165	MECP2_E1_s_1683	(mU)#(mU)#(fC)(mA)(fG)(mU)(fA)(mA)(fA)(mA)(mA)(mA)(fA)#(mA)#(mA)-TegChol
8213	MECP2_E1_as_1683	P(mU)#(fU)#(mU)(fU)(fU)(fU)(mU)(fU)(mU)(fA)(mC)(fU)(mG)#(fA)#(mA)#(fA)#(mG)#(mA)#(mA)#(fA)
8163	MECP2_E1_s_1684	(mU)#(mC)#(fA)(mG)(fU)(mA)(fA)(mA)(fA)(mA)(mA)(mA)(fA)#(mA)#(mA)-TegChol
8211	MECP2_E1_as_1684	P(mU)#(fU)#(mU)(fU)(fU)(fU)(mU)(fU)(mU)(fU)(mA)(fC)(mU)#(fG)#(mA)#(fA)#(mA)#(mG)#(mA)#(fA)
8157	MECP2_E1_s_1685	(mC)#(mA)#(fG)(mU)(fA)(mA)(fA)(mA)(fA)(mA)(mA)(mA)(fA)#(mA)#(mA)-TegChol
8205	MECP2_E1_as_1685	P(mU)#(fU)#(mU)(fU)(fU)(fU)(mU)(fU)(mU)(fU)(mU)(fA)(mC)#(fU)#(mG)#(fA)#(mA)#(mA)#(mG)#(fA)
8160	MECP2_E1_s_1686	(mA)#(mG)#(fU)(mA)(fA)(mA)(fA)(mA)(fA)(mA)(mA)(mA)(fA)#(mA)#(mA)-TegChol
8208	MECP2_E1_as_1686	P(mU)#(fU)#(mU)(fU)(fU)(fU)(mU)(fU)(mU)(fU)(mU)(fU)(mA)#(fC)#(mU)#(fG)#(mA)#(mA)#(mA)#(fG)

8161	MECP2_E1_s_1687	(mG)#(mU)#(fA)(mA)(fA)(mA)(fA)(mA)(fA)(mA)(mA)(mA)(fA)#(mA)#(mA)-TegChol
8209	MECP2_E1_as_1687	P(mU)#(fU)#(mU)(fU)(fU)(fU)(mU)(fU)(mU)(fU)(mU)(fU)(mU)#(fA)#(mC)#(fU)#(mG)#(mA)#(mA)#(fA)
8156	MECP2_E1_s_1688	(mU)#(mA)#(fA)(mA)(fA)(mA)(fA)(mA)(fA)(mA)(mA)(mA)(fA)#(mA)#(mA)-TegChol
8204	MECP2_E1_as_1688	P(mU)#(fU)#(mU)(fU)(fU)(fU)(mU)(fU)(mU)(fU)(mU)(fU)(mU)#(fU)#(mA)#(fC)#(mU)#(mG)#(mA)#(fA)
8154	MECP2_E1_s_1689	(mA)#(mA)#(fA)(mA)(fA)(mA)(fA)(mA)(fA)(mA)(mA)(mA)(fA)#(mA)#(mA)-TegChol
8202	MECP2_E1_as_1689	P(mU)#(fU)#(mU)(fU)(fU)(fU)(mU)(fU)(mU)(fU)(mU)(fU)(mU)#(fU)#(mU)#(fA)#(mC)#(mU)#(mG)#(fA)
8180	MECP2_E2_s_113	(mG)#(mA)#(fG)(mG)(fA)(mG)(fA)(mG)(fA)(mC)(mU)(mG)(fC)#(mU)#(mA)-TegChol
8228	MECP2_E2_as_113	P(mU)#(fA)#(mG)(fC)(fA)(fG)(mU)(fC)(mU)(fC)(mU)(fC)(mC)#(fU)#(mC)#(fC)#(mU)#(mC)#(mG)#(fC)
8189	MECP2_E2_s_128	(mC)#(mA)#(fU)(mA)(fA)(mA)(fA)(mA)(fU)(mA)(mC)(mA)(fG)#(mA)#(mA)-TegChol
8237	MECP2_E2_as_128	P(mU)#(fU)#(mC)(fU)(fG)(fU)(mA)(fU)(mU)(fU)(mU)(fU)(mA)#(fU)#(mG)#(fG)#(mA)#(mG)#(mC)#(fA)
8182	MECP2_E2_s_131	(mA)#(mA)#(fA)(mA)(fA)(mU)(fA)(mC)(fA)(mG)(mA)(mC)(fU)#(mC)#(mA)-TegChol
8230	MECP2_E2_as_131	P(mU)#(fG)#(mA)(fG)(fU)(fC)(mU)(fG)(mU)(fA)(mU)(fU)(mU)#(fU)#(mU)#(fA)#(mU)#(mG)#(mG)#(fA)
8181	MECP2_E2_s_146	(mC)#(mC)#(fA)(mG)(fU)(mU)(fC)(mC)(fU)(mG)(mC)(mU)(fU)#(mU)#(mA)-TegChol
8229	MECP2_E2_as_146	P(mU)#(fA)#(mA)(fA)(fG)(fC)(mA)(fG)(mG)(fA)(mA)(fC)(mU)#(fG)#(mG)#(fU)#(mG)#(mA)#(mG)#(fU)
8188	MECP2_E2_s_147	(mC)#(mA)#(fG)(mU)(fU)(mC)(fC)(mU)(fG)(mC)(mU)(mU)(fU)#(mG)#(mA)-TegChol
8236	MECP2_E2_as_147	P(mU)#(fC)#(mA)(fA)(fA)(fG)(mC)(fA)(mG)(fG)(mA)(fA)(mC)#(fU)#(mG)#(fG)#(mU)#(mG)#(mA)#(fG)
8184	MECP2_E2_s_174	(mC)#(mU)#(fC)(mC)(fC)(mC)(fA)(mG)(fA)(mA)(mU)(mA)(fC)#(mA)#(mA)-TegChol
8232	MECP2_E2_as_174	P(mU)#(fU)#(mG)(fU)(fA)(fU)(mU)(fC)(mU)(fG)(mG)(fG)(mG)#(fA)#(mG)#(fU)#(mC)#(mA)#(mC)#(fA)
8179	MECP2_E2_s_179	(mC)#(mA)#(fG)(mA)(fA)(mU)(fA)(mC)(fA)(mC)(mC)(mU)(fU)#(mG)#(mA)-TegChol
8227	MECP2_E2_as_179	P(mU)#(fC)#(mA)(fA)(fG)(fG)(mU)(fG)(mU)(fA)(mU)(fU)(mC)#(fU)#(mG)#(fG)#(mG)#(mG)#(mA)#(fG)

8178	MECP2_E2_s_181	(mG)#(mA)#(fA)(mU)(fA)(mC)(fA)(mC)(fC)(mU)(mU)(mG)(fC)#(mU)#(mA)-TegChol
8226	MECP2_E2_as_181	P(mU)#(fA)#(mG)(fC)(fA)(fA)(mG)(fG)(mU)(fG)(mU)(fA)(mU)#(fU)#(mC)#(fU)#(mG)#(mG)#(mG)#(fG)
8183	MECP2_E2_s_213	(mC)#(mA)#(fG)(mG)(fA)(mU)(fU)(mC)(fC)(mA)(mU)(mG)(fG)#(mU)#(mA)-TegChol
8231	MECP2_E2_as_213	P(mU)#(fA)#(mC)(fC)(fA)(fU)(mG)(fG)(mA)(fA)(mU)(fC)(mC)#(fU)#(mG)#(fU)#(mU)#(mG)#(mG)#(fA)
8186	MECP2_E2_s_214	(mA)#(mG)#(fG)(mA)(fU)(mU)(fC)(mC)(fA)(mU)(mG)(mG)(fU)#(mA)#(mA)-TegChol
8234	MECP2_E2_as_214	P(mU)#(fU)#(mA)(fC)(fC)(fA)(mU)(fG)(mG)(fA)(mA)(fU)(mC)#(fC)#(mU)#(fG)#(mU)#(mU)#(mG)#(fG)
8185	MECP2_E2_s_233	(mG)#(mA)#(fU)(mG)(fU)(mU)(fA)(mG)(fG)(mG)(mC)(mU)(fC)#(mA)#(mA)-TegChol
8233	MECP2_E2_as_233	P(mU)#(fU)#(mG)(fA)(fG)(fC)(mC)(fC)(mU)(fA)(mA)(fC)(mA)#(fU)#(mC)#(fC)#(mC)#(mA)#(mG)#(fC)
8187	MECP2_E2_s_234	(mA)#(mU)#(fG)(mU)(fU)(mA)(fG)(mG)(fG)(mC)(mU)(mC)(fA)#(mG)#(mA)-TegChol
8235	MECP2_E2_as_234	P(mU)#(fC)#(mU)(fG)(fA)(fG)(mC)(fC)(mC)(fU)(mA)(fA)(mC)#(fA)#(mU)#(fC)#(mC)#(mC)#(mA)#(fG)
8201	MECP2_E2_s_3514	(mA)#(mA)#(fU)(mG)(fG)(mC)(fA)(mA)(fU)(mG)(mU)(mU)(fU)#(mU)#(mA)-TegChol
8249	MECP2_E2_as_3514	P(mU)#(fA)#(mA)(fA)(fA)(fC)(mA)(fU)(mU)(fG)(mC)(fC)(mA)#(fU)#(mU)#(fC)#(mA)#(mA)#(mG)#(fA)
8197	MECP2_E2_s_3754	(mU)#(mA)#(fU)(mA)(fU)(mC)(fU)(mA)(fA)(mA)(mU)(mC)(fU)#(mG)#(mA)-TegChol
8245	MECP2_E2_as_3754	P(mU)#(fC)#(mA)(fG)(fA)(fU)(mU)(fU)(mA)(fG)(mA)(fU)(mA)#(fU)#(mA)#(fA)#(mG)#(mA)#(mG)#(fA)
8193	MECP2_E2_s_4431	(mU)#(mU)#(fU)(mU)(fU)(mA)(fU)(mG)(fU)(mA)(mU)(mU)(fA)#(mU)#(mA)-TegChol
8241	MECP2_E2_as_4431	P(mU)#(fA)#(mU)(fA)(fA)(fU)(mA)(fC)(mA)(fU)(mA)(fA)(mA)#(fA)#(mA)#(fC)#(mC)#(mC)#(mA)#(fA)
8200	MECP2_E2_s_5875	(mG)#(mC)#(fA)(mU)(fA)(mU)(fA)(mC)(fA)(mU)(mU)(mU)(fU)#(mU)#(mA)-TegChol
8248	MECP2_E2_as_5875	P(mU)#(fA)#(mA)(fA)(fA)(fA)(mU)(fG)(mU)(fA)(mU)(fA)(mU)#(fG)#(mC)#(fC)#(mC)#(mA)#(mA)#(fA)
8190	MECP2_E2_s_5876	(mC)#(mA)#(fU)(mA)(fU)(mA)(fC)(mA)(fU)(mU)(mU)(mU)(fU)#(mA)#(mA)-TegChol

8238	MECP2_E2_as_5876	P(mU)#(fU)#(mA)(fA)(fA)(fA)(mA)(fU)(mG)(fU)(mA)(fU)(mA)#(fU)#(mG)#(fC)#(mC)#(mC)#(mA)#(fA)
8198	MECP2_E2_s_5877	(mA)#(mU)#(fA)(mU)(fA)(mC)(fA)(mU)(fU)(mU)(mU)(mU)(fA)#(mG)#(mA)-TegChol
8246	MECP2_E2_as_5877	P(mU)#(fC)#(mU)(fA)(fA)(fA)(mA)(fA)(mU)(fG)(mU)(fA)(mU)#(fA)#(mU)#(fG)#(mC)#(mC)#(mC)#(fA)
8199	MECP2_E2_s_6278	(mC)#(mA)#(fG)(mA)(fA)(mA)(fA)(mU)(fU)(mA)(mC)(mA)(fU)#(mU)#(mA)-TegChol
8247	MECP2_E2_as_6278	P(mU)#(fA)#(mA)(fU)(fG)(fU)(mA)(fA)(mU)(fU)(mU)(fU)(mC)#(fU)#(mG)#(fC)#(mC)#(mA)#(mA)#(fA)
8196	MECP2_E2_s_7175	(mU)#(mA)#(fU)(mU)(fG)(mC)(fA)(mC)(fA)(mA)(mU)(mU)(fA)#(mU)#(mA)-TegChol
8244	MECP2_E2_as_7175	P(mU)#(fA)#(mU)(fA)(fA)(fU)(mU)(fG)(mU)(fG)(mC)(fA)(mA)#(fU)#(mA)#(fU)#(mA)#(mC)#(mA)#(fG)
8195	MECP2_E2_s_7237	(mA)#(mA)#(fU)(mU)(fA)(mU)(fA)(mC)(fC)(mU)(mG)(mU)(fU)#(mG)#(mA)-TegChol
8243	MECP2_E2_as_7237	P(mU)#(fC)#(mA)(fA)(fC)(fA)(mG)(fG)(mU)(fA)(mU)(fA)(mA)#(fU)#(mU)#(fU)#(mU)#(mA)#(mA)#(fC)
8191	MECP2_E2_s_8813	(mG)#(mU)#(fG)(mA)(fA)(mA)(fG)(mG)(fA)(mA)(mU)(mU)(fU)#(mU)#(mA)-TegChol
8239	MECP2_E2_as_8813	P(mU)#(fA)#(mA)(fA)(fA)(fU)(mU)(fC)(mC)(fU)(mU)(fU)(mC)#(fA)#(mC)#(fC)#(mC)#(mA)#(mC)#(fC)
8194	MECP2_E2_s_8893	(mC)#(mA)#(fG)(mA)(fG)(mA)(fC)(mA)(fA)(mA)(mU)(mA)(fU)#(mU)#(mA)-TegChol
8242	MECP2_E2_as_8893	P(mU)#(fA)#(mA)(fU)(fA)(fU)(mU)(fU)(mG)(fU)(mC)(fU)(mC)#(fU)#(mG)#(fG)#(mA)#(mA)#(mC)#(fA)
8192	MECP2_E2_s_9967	(mU)#(mU)#(fU)(mG)(fG)(mG)(fA)(mC)(fA)(mA)(mU)(mU)(fA)#(mC)#(mA)-TegChol
8240	MECP2_E2_as_9967	P(mU)#(fG)#(mU)(fA)(fA)(fU)(mU)(fG)(mU)(fC)(mC)(fC)(mA)#(fA)#(mA)#(fA)#(mC)#(mA)#(mC)#(fA)

P=phosphate, #=phosphorothioate, m=2'-Omethyl, f=2'-fluoro, A=adenine, U=uracil, G=guanine, C=cytosine, TegChol = cholesterol conjugate with linker

Supplementary Table S2. Nest building rating scale

Score		Definition
Nestlet Manipulation Score	0	Nestlet is untouched.
	1	It is not clear where the nest is located in the cage (i.e., the nestlet material is spread throughout the cage).
	2	The nest is flat with no shallow walls, centralized in one spot.

Nest Site Score	3	A slightly cupped shape where the walls of the nest are less than ½ the height of a dome that would cover a mouse.
	4	Walls that are ½ the height of a dome that would cover a mouse.
	5	Walls that are more than ½ the height of a dome that would cover a mouse, which may or may not fully enclose the nest.

Supplementary Table S3. Statistical analysis tables and exact p-values for figures

Figure 1A

Dunnett's multiple comparisons test	Mean Diff.	95.00% CI of diff.	Below threshold?	Summary	Adjusted P Value
UNT vs. NTC	11.56	-23.37 to 46.49	No	ns	0.9923
UNT vs. 575	20.80	-14.13 to 55.73	No	ns	0.5738
UNT vs. 572	19.93	-15.00 to 54.85	No	ns	0.6324
UNT vs. 300	11.03	-23.90 to 45.96	No	ns	0.9955
UNT vs. 827	-16.11	-51.03 to 18.82	No	ns	0.8686
UNT vs. 603	19.32	-15.61 to 54.24	No	ns	0.6734
UNT vs. 887	52.66	17.74 to 87.59	Yes	***	0.0005
UNT vs. 285	20.07	-14.86 to 55.00	No	ns	0.6226
UNT vs. 605	9.517	-25.41 to 44.44	No	ns	0.9993
UNT vs. 662	24.37	-10.56 to 59.30	No	ns	0.3569
UNT vs. 535	31.27	-3.661 to 66.19	No	ns	0.1075
UNT vs. 303	52.15	17.22 to 87.08	Yes	***	0.0006
UNT vs. 895	39.83	4.903 to 74.76	Yes	*	0.0160
UNT vs. 1776	57.62	22.69 to 92.55	Yes	***	0.0001
UNT vs. 1785	19.02	-15.90 to 53.95	No	ns	0.6931
UNT vs. 1759	67.35	32.42 to 102.3	Yes	****	<0.0001
UNT vs. 1123	13.27	-21.66 to 48.20	No	ns	0.9693
UNT vs. 1787	2.960	-31.97 to 37.89	No	ns	>0.9999
UNT vs. 1784	13.04	-21.89 to 47.97	No	ns	0.9738
UNT vs. 1786	5.693	-29.23 to 40.62	No	ns	>0.9999
UNT vs. 1788	28.15	-6.774 to 63.08	No	ns	0.1926

UNT vs. 1758	51.49	16.57 to 86.42	Yes	***	0.0007
UNT vs. 1669	12.76	-22.17 to 47.69	No	ns	0.9786
UNT vs. 1670	18.50	-16.43 to 53.43	No	ns	0.7277
UNT vs. 1764	54.70	19.77 to 89.63	Yes	***	0.0003

Figure 1D

Dunnett's multiple comparisons test	Mean Diff.	95.00% CI of diff.	Below threshold?	Summary	Adjusted P Value
Isoform E1					
UNT vs. NTC	-55.82	-123.0 to 11.36	No	ns	0.1711
UNT vs. 111	87.54	20.37 to 154.7	Yes	**	0.0030
UNT vs. 112	97.47	30.30 to 164.6	Yes	***	0.0006
UNT vs. 113	62.32	-4.852 to 129.5	No	ns	0.0873
UNT vs. 114	57.80	-9.372 to 125.0	No	ns	0.1406
UNT vs. 115	76.84	9.667 to 144.0	Yes	*	0.0144
UNT vs. 117	65.50	-1.667 to 132.7	No	ns	0.0609
UNT vs. 118	50.35	-16.82 to 117.5	No	ns	0.2811
UNT vs. 121	33.77	-33.40 to 100.9	No	ns	0.8105
UNT vs. 122	43.62	-23.55 to 110.8	No	ns	0.4731
UNT vs. 123	17.43	-49.74 to 84.60	No	ns	0.9998
UNT vs. 125	30.05	-37.12 to 97.22	No	ns	0.9096
UNT vs. 127	44.64	-22.53 to 111.8	No	ns	0.4401
UNT vs. 1678	-15.30	-82.47 to 51.87	No	ns	>0.9999
UNT vs. 1679	-7.922	-75.09 to 59.25	No	ns	>0.9999
UNT vs. 1680	2.180	-64.99 to 69.35	No	ns	>0.9999
UNT vs. 1681	-52.78	-119.9 to 14.40	No	ns	0.2273
UNT vs. 1682	-1.049	-68.22 to 66.12	No	ns	>0.9999
UNT vs. 1683	17.87	-49.30 to 85.04	No	ns	0.9998
UNT vs. 1684	15.62	-51.55 to 82.79	No	ns	>0.9999
UNT vs. 1685	14.51	-52.66 to 81.68	No	ns	>0.9999
UNT vs. 1686	9.895	-57.28 to 77.07	No	ns	>0.9999

UNT vs. 1687	11.29	-55.88 to 78.46	No	ns	>0.9999
UNT vs. 1688	44.52	-22.65 to 111.7	No	ns	0.4440
UNT vs. 1689	8.293	-58.88 to 75.46	No	ns	>0.9999
Isoform E2					
UNT vs. NTC	-36.40	-103.6 to 30.77	No	ns	0.7239
UNT vs. 111	12.33	-54.84 to 79.50	No	ns	>0.9999
UNT vs. 112	28.05	-39.12 to 95.22	No	ns	0.9469
UNT vs. 113	22.84	-44.34 to 90.01	No	ns	0.9932
UNT vs. 114	14.66	-52.51 to 81.83	No	ns	>0.9999
UNT vs. 115	22.01	-45.16 to 89.18	No	ns	0.9956
UNT vs. 117	10.71	-56.46 to 77.88	No	ns	>0.9999
UNT vs. 118	-1.692	-68.86 to 65.48	No	ns	>0.9999
UNT vs. 121	7.009	-60.16 to 74.18	No	ns	>0.9999
UNT vs. 122	28.50	-38.67 to 95.67	No	ns	0.9396
UNT vs. 123	5.514	-61.66 to 72.68	No	ns	>0.9999
UNT vs. 125	12.13	-55.04 to 79.30	No	ns	>0.9999
UNT vs. 127	21.09	-46.08 to 88.26	No	ns	0.9974
UNT vs. 1678	16.56	-50.61 to 83.73	No	ns	>0.9999
UNT vs. 1679	2.162	-65.01 to 69.33	No	ns	>0.9999
UNT vs. 1680	-8.218	-75.39 to 58.95	No	ns	>0.9999
UNT vs. 1681	-94.57	-161.7 to -27.40	Yes	***	0.0010
UNT vs. 1682	-18.94	-86.11 to 48.23	No	ns	0.9994
UNT vs. 1683	-2.590	-69.76 to 64.58	No	ns	>0.9999
UNT vs. 1684	20.51	-46.66 to 87.68	No	ns	0.9982
UNT vs. 1685	-9.173	-76.34 to 58.00	No	ns	>0.9999
UNT vs. 1686	-15.66	-82.83 to 51.51	No	ns	>0.9999
UNT vs. 1687	-3.418	-70.59 to 63.75	No	ns	>0.9999
UNT vs. 1688	3.684	-63.49 to 70.86	No	ns	>0.9999
UNT vs. 1689	3.979	-63.19 to 71.15	No	ns	>0.9999

Figure 1E

Dunnett's multiple comparisons test	Mean Diff.	95.00% CI of diff.	Below threshold?	Summary	Adjusted P Value
UNT vs. NTC	14.05	-38.84 to 66.93	No	ns	0.9995
UNT vs. 111	-87.13	-140.0 to -34.24	Yes	***	0.0001
UNT vs. 112	-96.67	-149.6 to -43.78	Yes	****	<0.0001
UNT vs. 113	-51.28	-104.2 to 1.610	No	ns	0.0630
UNT vs. 114	-49.97	-102.9 to 2.921	No	ns	0.0756
UNT vs. 115	-70.76	-123.6 to -17.87	Yes	**	0.0026
UNT vs. 117	-60.52	-113.4 to -7.638	Yes	*	0.0154
UNT vs. 118	-51.08	-104.0 to 1.811	No	ns	0.0648
UNT vs. 121	-29.13	-82.02 to 23.75	No	ns	0.6785
UNT vs. 122	-14.64	-67.52 to 38.25	No	ns	0.9992
UNT vs. 123	-12.70	-65.59 to 40.19	No	ns	0.9999
UNT vs. 125	-20.71	-73.60 to 32.17	No	ns	0.9600
UNT vs. 127	-30.31	-83.19 to 22.58	No	ns	0.6264
UNT vs. 1678	46.52	-6.362 to 99.41	No	ns	0.1195
UNT vs. 1679	12.29	-40.60 to 65.18	No	ns	>0.9999
UNT vs. 1680	-9.753	-62.64 to 43.13	No	ns	>0.9999
UNT vs. 1681	-22.17	-75.06 to 30.72	No	ns	0.9312
UNT vs. 1682	-15.51	-68.40 to 37.38	No	ns	0.9982
UNT vs. 1683	-18.99	-71.88 to 33.89	No	ns	0.9819
UNT vs. 1684	29.13	-23.75 to 82.02	No	ns	0.6787
UNT vs. 1685	-21.60	-74.49 to 31.28	No	ns	0.9436
UNT vs. 1686	-22.34	-75.23 to 30.54	No	ns	0.9272
UNT vs. 1687	-15.18	-68.07 to 37.70	No	ns	0.9986
UNT vs. 1688	-42.37	-95.25 to 10.52	No	ns	0.1986
UNT vs. 1689	-4.703	-57.59 to 48.18	No	ns	>0.9999

Figure 2C – 1 month

Dunnett's multiple comparisons test	Predicted (LS) mean diff.	95.00% CI of diff.	Below threshold?	Summary	Adjusted P Value

Cortex					
NTC vs. 1759_S1	73.95	46.91 to 101.0	Yes	****	<0.0001
NTC vs. 1764_S1	69.90	46.49 to 93.32	Yes	****	<0.0001
NTC vs. 112_S1	28.39	4.977 to 51.81	Yes	*	0.0106
NTC vs. 1759_S2	63.69	38.85 to 88.53	Yes	****	<0.0001
NTC vs. 1764_S2	68.26	44.84 to 91.68	Yes	****	<0.0001
NTC vs. 112_S2	35.34	11.93 to 58.76	Yes	***	0.0008
Striatum					
NTC vs. 1759_S1	71.23	45.27 to 97.19	Yes	****	<0.0001
NTC vs. 1764_S1	72.25	47.62 to 96.87	Yes	****	<0.0001
NTC vs. 112_S1	19.85	-<br>4.783 to 44.47	No	ns	0.1590
NTC vs. 1759_S2	71.01	45.05 to 96.97	Yes	****	<0.0001
NTC vs. 1764_S2	58.95	34.32 to 83.58	Yes	****	<0.0001
NTC vs. 112_S2	40.88	16.25 to 65.50	Yes	***	0.0002
Hippocampus					
NTC vs. 1759_S1	60.98	36.17 to 85.78	Yes	****	<0.0001
NTC vs. 1764_S1	70.68	47.29 to 94.06	Yes	****	<0.0001
NTC vs. 112_S1	32.03	8.641 to 55.41	Yes	**	0.0029
NTC vs. 1759_S2	67.45	42.65 to 92.26	Yes	****	<0.0001
NTC vs. 1764_S2	66.85	43.46 to 90.23	Yes	****	<0.0001
NTC vs. 112_S2	41.29	17.91 to 64.68	Yes	****	<0.0001
Cerebellum					
NTC vs. 1759_S1	57.03	32.22 to 81.83	Yes	****	<0.0001
NTC vs. 1764_S1	61.42	38.04 to 84.81	Yes	****	<0.0001
NTC vs. 112_S1	12.01	-<br>11.38 to 35.39	No	ns	0.5888
NTC vs. 1759_S2	46.84	22.04 to 71.65	Yes	****	<0.0001
NTC vs. 1764_S2	28.94	5.557 to 52.33	Yes	**	0.0087
NTC vs. 112_S2	28.51	5.129 to 51.90	Yes	*	0.0101

Figure 2C – 4 months

Dunnett's multiple comparisons test	Predicted (LS) Mean diff.	95.00% CI of diff.	Below threshold?	Summary	Adjusted P Value
Cortex					
NTC vs. 1759_S1	46.18	18.44 to 73.93	Yes	***	0.0002
NTC vs. 1764_S1	64.94	37.19 to 92.68	Yes	****	<0.0001
NTC vs. 112_S1	36.89	10.32 to 63.45	Yes	**	0.0024
NTC vs. 1759_S2	59.54	27.50 to 91.58	Yes	****	<0.0001
NTC vs. 1764_S2	53.84	26.10 to 81.59	Yes	****	<0.0001
NTC vs. 112_S2	16.03	-11.71 to 43.78	No	ns	0.4687
Striatum					
NTC vs. 1759_S1	52.86	25.15 to 80.57	Yes	****	<0.0001
NTC vs. 1764_S1	62.99	35.28 to 90.69	Yes	****	<0.0001
NTC vs. 112_S1	22.28	-4.244 to 48.81	No	ns	0.1338
NTC vs. 1759_S2	64.75	35.36 to 94.14	Yes	****	<0.0001
NTC vs. 1764_S2	51.44	23.73 to 79.14	Yes	****	<0.0001
NTC vs. 112_S2	16.53	-11.18 to 44.24	No	ns	0.4332
Hippocampus					
NTC vs. 1759_S1	56.89	29.18 to 84.60	Yes	****	<0.0001
NTC vs. 1764_S1	72.07	44.36 to 99.77	Yes	****	<0.0001
NTC vs. 112_S1	42.06	15.53 to 68.59	Yes	***	0.0004
NTC vs. 1759_S2	65.33	35.94 to 94.72	Yes	****	<0.0001
NTC vs. 1764_S2	68.11	40.40 to 95.82	Yes	****	<0.0001
NTC vs. 112_S2	31.30	3.590 to 59.01	Yes	*	0.0201
Cerebellum					
NTC vs. 1759_S1	12.16	-15.55 to 39.87	No	ns	0.7258
NTC vs. 1764_S1	51.73	24.02 to 79.44	Yes	****	<0.0001
NTC vs. 112_S1	19.57	-6.954 to 46.10	No	ns	0.2301
NTC vs. 1759_S2	36.74	7.355 to 66.13	Yes	**	0.0079

NTC vs. 1764_S2	25.93	-1.782 to 53.63	No	ns	0.0757
NTC vs. 112_S2	20.36	-7.349 to 48.07	No	ns	0.2337

Figure 2D – 1 month

Dunnett's multiple comparisons test	Predicted (LS) Mean diff.	95.00% CI of diff.	Below threshold?	Summary	Adjusted P Value
Cortex					
NTC vs. 1759_S1	78.46	51.45 to 105.5	Yes	****	<0.0001
NTC vs. 1764_S1	75.85	52.46 to 99.23	Yes	****	<0.0001
NTC vs. 112_S1	64.25	40.86 to 87.63	Yes	****	<0.0001
NTC vs. 1759_S2	78.91	54.10 to 103.7	Yes	****	<0.0001
NTC vs. 1764_S2	78.23	54.84 to 101.6	Yes	****	<0.0001
NTC vs. 112_S2	83.72	60.33 to 107.1	Yes	****	<0.0001
Striatum					
NTC vs. 1759_S1	78.55	53.78 to 103.3	Yes	****	<0.0001
NTC vs. 1764_S1	82.57	59.22 to 105.9	Yes	****	<0.0001
NTC vs. 112_S1	72.98	49.62 to 96.33	Yes	****	<0.0001
NTC vs. 1759_S2	81.58	56.80 to 106.4	Yes	****	<0.0001
NTC vs. 1764_S2	79.81	56.45 to 103.2	Yes	****	<0.0001
NTC vs. 112_S2	91.04	67.68 to 114.4	Yes	****	<0.0001
Hippocampus					
NTC vs. 1759_S1	69.41	42.40 to 96.41	Yes	****	<0.0001
NTC vs. 1764_S1	73.47	50.08 to 96.86	Yes	****	<0.0001
NTC vs. 112_S1	60.05	36.66 to 83.44	Yes	****	<0.0001
NTC vs. 1759_S2	63.45	38.64 to 88.26	Yes	****	<0.0001
NTC vs. 1764_S2	67.79	44.40 to 91.18	Yes	****	<0.0001
NTC vs. 112_S2	78.94	55.55 to 102.3	Yes	****	<0.0001
Cerebellum					
NTC vs. 1759_S1	61.61	36.84 to 86.38	Yes	****	<0.0001

NTC vs. 1764_S1	67.99	44.63 to 91.35	Yes	****	<0.0001
NTC vs. 112_S1	48.40	25.04 to 71.76	Yes	****	<0.0001
NTC vs. 1759_S2	50.23	25.45 to 75.00	Yes	****	<0.0001
NTC vs. 1764_S2	45.25	21.90 to 68.61	Yes	****	<0.0001
NTC vs. 112_S2	72.04	48.68 to 95.40	Yes	****	<0.0001

Figure 2D – 4 months

Dunnett's multiple comparisons test	Predicted (LS) mean diff.	95.00% CI of diff.	Below threshold?	Summary	Adjusted P Value
Cortex					
NTC vs. 1759_S1	61.05	31.59 to 90.52	Yes	****	<0.0001
NTC vs. 1764_S1	73.78	44.31 to 103.2	Yes	****	<0.0001
NTC vs. 112_S1	62.34	34.12 to 90.55	Yes	****	<0.0001
NTC vs. 1759_S2	58.74	24.71 to 92.77	Yes	****	<0.0001
NTC vs. 1764_S2	53.41	23.94 to 82.88	Yes	****	<0.0001
NTC vs. 112_S2	66.90	37.44 to 96.37	Yes	****	<0.0001
Striatum					
NTC vs. 1759_S1	61.05	31.62 to 90.48	Yes	****	<0.0001
NTC vs. 1764_S1	72.08	42.65 to 101.5	Yes	****	<0.0001
NTC vs. 112_S1	57.91	29.73 to 86.08	Yes	****	<0.0001
NTC vs. 1759_S2	66.12	34.91 to 97.34	Yes	****	<0.0001
NTC vs. 1764_S2	56.06	26.63 to 85.49	Yes	****	<0.0001
NTC vs. 112_S2	82.57	53.14 to 112.0	Yes	****	<0.0001
Hippocampus					
NTC vs. 1759_S1	63.43	34.00 to 92.86	Yes	****	<0.0001
NTC vs. 1764_S1	74.53	45.10 to 104.0	Yes	****	<0.0001
NTC vs. 112_S1	61.84	33.66 to 90.01	Yes	****	<0.0001
NTC vs. 1759_S2	67.42	36.21 to 98.64	Yes	****	<0.0001
NTC vs. 1764_S2	68.69	39.26 to 98.12	Yes	****	<0.0001
NTC vs. 112_S2	72.44	43.01 to 101.9	Yes	****	<0.0001
Cerebellum					

NTC vs. 1759_S1	18.99	-10.44 to 48.42	No	ns	0.3540
NTC vs. 1764_S1	55.32	25.90 to 84.75	Yes	****	<0.0001
NTC vs. 112_S1	34.90	6.727 to 63.08	Yes	**	0.0087
NTC vs. 1759_S2	35.82	4.604 to 67.03	Yes	*	0.0176
NTC vs. 1764_S2	25.55	-3.884 to 54.97	No	ns	0.1139
NTC vs. 112_S2	39.61	10.18 to 69.04	Yes	**	0.0036

Figure 2E – 1 month

Dunnett's multiple comparisons test	Predicted (LS) Mean diff.	95.00% CI of diff.	Below threshold?	Summary	Adjusted P Value
Cortex					
NTC vs. 1759_S1	85.90	45.43 to 126.4	Yes	****	<0.0001
NTC vs. 1764_S1	80.68	45.64 to 115.7	Yes	****	<0.0001
NTC vs. 112_S1	35.45	0.4044 to 70.49	Yes	*	0.0463
NTC vs. 1759_S2	82.75	45.58 to 119.9	Yes	****	<0.0001
NTC vs. 1764_S2	75.32	40.28 to 110.4	Yes	****	<0.0001
NTC vs. 112_S2	32.77	-2.270 to 67.81	No	ns	0.0761
Striatum					
NTC vs. 1759_S1	87.38	50.26 to 124.5	Yes	****	<0.0001
NTC vs. 1764_S1	82.00	47.00 to 117.0	Yes	****	<0.0001
NTC vs. 112_S1	51.63	16.63 to 86.63	Yes	**	0.0011
NTC vs. 1759_S2	82.61	45.49 to 119.7	Yes	****	<0.0001
NTC vs. 1764_S2	75.67	40.67 to 110.7	Yes	****	<0.0001
NTC vs. 112_S2	50.11	15.11 to 85.11	Yes	**	0.0017
Hippocampus					
NTC vs. 1759_S1	70.62	30.16 to 111.1	Yes	****	<0.0001
NTC vs. 1764_S1	75.21	40.17 to 110.3	Yes	****	<0.0001
NTC vs. 112_S1	21.92	-13.12 to 56.97	No	ns	0.3901

NTC vs. 1759_S2	65.78	28.61 to 102.9	Yes	****	<0.0001
NTC vs. 1764_S2	65.36	30.32 to 100.4	Yes	****	<0.0001
NTC vs. 112_S2	8.116	-26.93 to 43.16	No	ns	0.9798
Cerebellum					
NTC vs. 1759_S1	74.46	37.34 to 111.6	Yes	****	<0.0001
NTC vs. 1764_S1	68.55	33.55 to 103.5	Yes	****	<0.0001
NTC vs. 112_S1	6.854	-28.14 to 41.85	No	ns	0.9911
NTC vs. 1759_S2	19.62	-17.50 to 56.74	No	ns	0.5601
NTC vs. 1764_S2	2.036	-32.96 to 37.03	No	ns	>0.9999
NTC vs. 112_S2	5.893	-29.10 to 40.89	No	ns	0.9960

Figure 2E – 4 months

Dunnett's multiple comparisons test	Predicted (LS) mean diff.	95.00% CI of diff.	Below threshold?	Summary	Adjusted P Value
Cortex					
NTC vs. 1759_S1	60.94	28.42 to 93.46	Yes	****	<0.0001
NTC vs. 1764_S1	75.07	42.55 to 107.6	Yes	****	<0.0001
NTC vs. 112_S1	42.08	10.95 to 73.21	Yes	**	0.0034
NTC vs. 1759_S2	70.70	33.15 to 108.2	Yes	****	<0.0001
NTC vs. 1764_S2	65.98	33.46 to 98.49	Yes	****	<0.0001
NTC vs. 112_S2	32.51	-0.008229 to 65.03	No	ns	0.0501
Striatum					
NTC vs. 1759_S1	68.89	36.42 to 101.4	Yes	****	<0.0001
NTC vs. 1764_S1	74.65	42.18 to 107.1	Yes	****	<0.0001
NTC vs. 112_S1	25.65	-5.440 to 56.74	No	ns	0.1455
NTC vs. 1759_S2	75.95	41.50 to 110.4	Yes	****	<0.0001
NTC vs. 1764_S2	67.64	35.17 to 100.1	Yes	****	<0.0001

NTC vs. 112_S2	24.66	-7.812 to 57.13	No	ns	0.2064
Hippocampus					
NTC vs. 1759_S1	70.00	37.49 to 102.5	Yes	****	<0.0001
NTC vs. 1764_S1	78.90	46.38 to 111.4	Yes	****	<0.0001
NTC vs. 112_S1	52.94	21.81 to 84.08	Yes	***	0.0001
NTC vs. 1759_S2	84.19	46.64 to 121.7	Yes	****	<0.0001
NTC vs. 1764_S2	75.48	42.96 to 108.0	Yes	****	<0.0001
NTC vs. 112_S2	42.35	9.830 to 74.86	Yes	**	0.0051
Cerebellum					
NTC vs. 1759_S1	8.186	-24.29 to 40.66	No	ns	0.9679
NTC vs. 1764_S1	46.19	13.72 to 78.67	Yes	**	0.0018
NTC vs. 112_S1	12.69	-18.40 to 43.78	No	ns	0.7804
NTC vs. 1759_S2	38.22	3.778 to 72.66	Yes	*	0.0233
NTC vs. 1764_S2	29.93	-2.540 to 62.41	No	ns	0.0825
NTC vs. 112_S2	38.08	5.605 to 70.55	Yes	*	0.0145

Figure 3B – Endogenous mouse gene

Šídák's multiple comparisons test	Predicted (LS) mean diff.	95.00% CI of diff.	Below threshold?	Summary	Adjusted P Value
1764_S1 - NTC					
Cortex	-65.22	-105.9 to -24.55	Yes	***	0.0009
Striatum	-79.76	-120.4 to -39.10	Yes	****	<0.0001
Hippocampus	-63.98	-104.6 to -23.31	Yes	**	0.0012
Cerebellum	-54.35	-95.02 to -13.68	Yes	**	0.0058

Figure 3B – Human transgene

Šídák's multiple comparisons test	Predicted (LS) mean diff.	95.00% CI of diff.	Below threshold?	Summary	Adjusted P Value
1764_S1 - NTC					

Cortex	-66.00	-109.4 to -22.60	Yes	**	0.0017
Striatum	-88.93	-132.3 to -45.53	Yes	****	<0.0001
Hippocampus	-57.41	-100.8 to -14.01	Yes	**	0.0063
Cerebellum	-60.63	-104.0 to -17.22	Yes	**	0.0038

Figure 3D – Endogenous mouse gene

Šídák's multiple comparisons test	Predicted (LS) mean diff.	95.00% CI of diff.	Below threshold?	Summary	Adjusted P Value
1764_S1 - NTC					
Cortex	-57.15	-91.75 to -22.55	Yes	***	0.0004
Striatum	-51.70	-86.30 to -17.10	Yes	**	0.0014
Hippocampus	-54.78	-89.39 to -20.18	Yes	***	0.0007
Cerebellum	-22.94	-57.54 to 11.66	No	ns	0.3207

Figure 3D – Human transgene

Šídák's multiple comparisons test	Predicted (LS) mean diff.	95.00% CI of diff.	Below threshold?	Summary	Adjusted P Value
1764_S1 - NTC					
Cortex	-51.19	-99.78 to -2.604	Yes	*	0.0352
Striatum	-52.95	-101.5 to -4.364	Yes	*	0.0276
Hippocampus	-48.92	-97.50 to -0.3301	Yes	*	0.0479
Cerebellum	-15.44	-64.02 to 33.15	No	ns	0.8819

Figure 3F

Šídák's multiple comparisons test	Mean diff.	95.00% CI of diff.	Below threshold?	Summary	Adjusted P Value
NTC - 1764_S1					
M Cortex	88.63	23.49 to 153.8	Yes	**	0.0038
Striatum	78.73	13.59 to 143.9	Yes	*	0.0119
Hippocampus	94.89	29.75 to 160.0	Yes	**	0.0018
Cerebellum	33.51	-31.63 to 98.66	No	ns	0.5681

Figure 4D – Nest manipulation score

Tukey's multiple comparisons test	Predicted (LS) mean diff.	95.00% CI of diff.	Below threshold?	Summary	Adjusted P Value
11 weeks					
WT - PBS vs. Tg3 - PBS	1.000	0.1908 to 1.809	Yes	**	0.0090
WT - PBS vs. Tg3 - 1764_S1	-0.07143	-0.8806 to 0.7377	No	ns	0.9956
WT - PBS vs. Tg3 - 112_S1	0.4780	-0.3466 to 1.303	No	ns	0.4325
Tg3 - PBS vs. Tg3 - 1764_S1	-1.071	-1.881 to -0.2623	Yes	**	0.0044
Tg3 - PBS vs. Tg3 - 112_S1	-0.5220	-1.347 to 0.3026	No	ns	0.3535
Tg3 - 1764_S1 vs. Tg3 - 112_S1	0.5495	-0.2751 to 1.374	No	ns	0.3081
21 weeks					
WT - PBS vs. Tg3 - PBS	1.686	0.8289 to 2.543	Yes	****	<0.0001
WT - PBS vs. Tg3 - 1764_S1	-0.2262	-1.068 to 0.6160	No	ns	0.8961
WT - PBS vs. Tg3 - 112_S1	1.686	0.8289 to 2.543	Yes	****	<0.0001

Tg3 - PBS vs. Tg3 - 1764_S1	-1.912	-2.737 to -1.088	Yes	****	<0.0001
Tg3 - PBS vs. Tg3 - 112_S1	0.000	-0.8397 to 0.8397	No	ns	>0.9999
Tg3 - 1764_S1 vs. Tg3 - 112_S1	1.912	1.088 to 2.737	Yes	****	<0.0001

Figure 4D – Unused nest material

Tukey's multiple comparisons test	Predicted (LS) mean diff.	95.00% CI of diff.	Below threshold?	Summary	Adjusted P Value
11 weeks					
WT - PBS vs. Tg3 - PBS	-0.8286	-1.283 to -0.3737	Yes	****	<0.0001
WT - PBS vs. Tg3 - 1764_S1	-0.07143	-0.5263 to 0.3834	No	ns	0.9766
WT - PBS vs. Tg3 - 112_S1	-0.5253	-0.9888 to -0.06178	Yes	*	0.0197
Tg3 - PBS vs. Tg3 - 1764_S1	0.7571	0.3023 to 1.212	Yes	***	0.0002
Tg3 - PBS vs. Tg3 - 112_S1	0.3033	-0.1602 to 0.7668	No	ns	0.3240
Tg3 - 1764_S1 vs. Tg3 - 112_S1	-0.4538	-0.9173 to 0.009644	No	ns	0.0573
21 weeks					
WT - PBS vs. Tg3 - PBS	-1.006	-1.488 to -0.5245	Yes	****	<0.0001
WT - PBS vs. Tg3 - 1764_S1	0.1345	-0.3389 to 0.6078	No	ns	0.8797
WT - PBS vs. Tg3 - 112_S1	-1.151	-1.633 to -0.6696	Yes	****	<0.0001
Tg3 - PBS vs. Tg3 - 1764_S1	1.141	0.6772 to 1.604	Yes	****	<0.0001

Tg3 - PBS vs. Tg3 - 112_S1	-0.1451	-0.6170 to 0.3269	No	ns	0.8527
Tg3 - 1764_S1 vs. Tg3 - 112_S1	-1.286	-1.749 to -0.8222	Yes	****	<0.0001

Figure 4E – Distance moved

Tukey's multiple comparisons test	Predicted (LS) mean diff.	95.00% CI of diff.	Below threshold?	Summary	Adjusted P Value
12 weeks					
WT - PBS vs. Tg3 - PBS	-70.54	-125.3 to -15.74	Yes	**	0.0059
WT - PBS vs. Tg3 - 1764_S1	-55.55	-110.4 to -0.7451	Yes	*	0.0457
WT - PBS vs. Tg3 - 112_S1	-63.74	-119.6 to -7.890	Yes	*	0.0186
Tg3 - PBS vs. Tg3 - 1764_S1	14.99	-39.81 to 69.79	No	ns	0.8909
Tg3 - PBS vs. Tg3 - 112_S1	6.803	-49.04 to 62.65	No	ns	0.9888
Tg3 - 1764_S1 vs. Tg3 - 112_S1	-8.189	-64.04 to 47.66	No	ns	0.9807
22 weeks					
WT - PBS vs. Tg3 - PBS	-95.01	-152.2 to -37.83	Yes	***	0.0002
WT - PBS vs. Tg3 - 1764_S1	-69.36	-125.8 to -12.92	Yes	**	0.0095
WT - PBS vs. Tg3 - 112_S1	-102.3	-160.6 to -43.97	Yes	****	<0.0001
Tg3 - PBS vs. Tg3 - 1764_S1	25.65	-29.91 to 81.22	No	ns	0.6241
Tg3 - PBS vs. Tg3 - 112_S1	-7.265	-64.72 to 50.19	No	ns	0.9875
Tg3 - 1764_S1 vs. Tg3 - 112_S1	-32.92	-89.64 to 23.80	No	ns	0.4314

Figure 4E – Vertical activity

Tukey's multiple comparisons test	Predicted (LS) mean diff.	95.00% CI of diff.	Below threshold?	Summary	Adjusted P Value
12 weeks					
WT - PBS vs. Tg3 - PBS	635.5	-196.9 to 1468	No	ns	0.1968
WT - PBS vs. Tg3 - 1764_S1	-751.2	-1584 to 81.22	No	ns	0.0921
WT - PBS vs. Tg3 - 112_S1	24.43	-823.9 to 872.7	No	ns	0.9998
Tg3 - PBS vs. Tg3 - 1764_S1	-1387	-2219 to -554.3	Yes	***	0.0002
Tg3 - PBS vs. Tg3 - 112_S1	-611.1	-1459 to 237.2	No	ns	0.2421
Tg3 - 1764_S1 vs. Tg3 - 112_S1	775.6	-72.65 to 1624	No	ns	0.0857
22 weeks					
WT - PBS vs. Tg3 - PBS	622.0	-243.7 to 1488	No	ns	0.2443
WT - PBS vs. Tg3 - 1764_S1	-1259	-2114 to -403.7	Yes	**	0.0012
WT - PBS vs. Tg3 - 112_S1	434.4	-448.3 to 1317	No	ns	0.5738
Tg3 - PBS vs. Tg3 - 1764_S1	-1881	-2724 to -1038	Yes	****	<0.0001
Tg3 - PBS vs. Tg3 - 112_S1	-187.6	-1058 to 683.2	No	ns	0.9427
Tg3 - 1764_S1 vs. Tg3 - 112_S1	1693	833.0 to 2554	Yes	****	<0.0001

Figure 4E – Time in center

Tukey's multiple comparisons test	Predicted (LS) mean diff.	95.00% CI of diff.	Below threshold?	Summary	Adjusted P Value
12 weeks					
WT - PBS vs. Tg3 - PBS	-8.924	-15.36 to -2.485	Yes	**	0.0026
WT - PBS vs. Tg3 - 1764_S1	-12.17	-18.61 to -5.731	Yes	****	<0.0001

WT - PBS vs. Tg3 - 112_S1	-7.590	-14.15 to -1.028	Yes	*	0.0166
Tg3 - PBS vs. Tg3 - 1764_S1	-3.246	-9.685 to 3.193	No	ns	0.5540
Tg3 - PBS vs. Tg3 - 112_S1	1.334	-5.227 to 7.896	No	ns	0.9512
Tg3 - 1764_S1 vs. Tg3 - 112_S1	4.581	-1.981 to 11.14	No	ns	0.2681
22 weeks					
WT - PBS vs. Tg3 - PBS	-11.00	-17.79 to -4.199	Yes	***	0.0003
WT - PBS vs. Tg3 - 1764_S1	-14.84	-21.53 to -8.156	Yes	****	<0.0001
WT - PBS vs. Tg3 - 112_S1	-12.04	-18.97 to -5.113	Yes	****	<0.0001
Tg3 - PBS vs. Tg3 - 1764_S1	-3.847	-10.40 to 2.707	No	ns	0.4213
Tg3 - PBS vs. Tg3 - 112_S1	-1.048	-7.851 to 5.756	No	ns	0.9778
Tg3 - 1764_S1 vs. Tg3 - 112_S1	2.799	-3.894 to 9.492	No	ns	0.6947

Figure 4F

Tukey's multiple comparisons test	Predicted (LS) mean diff.	95.00% CI of diff.	Below threshold?	Summary	Adjusted P Value
13 weeks					
WT - PBS vs. Tg3 - PBS	-1.256	-64.99 to 62.48	No	ns	>0.9999
WT - PBS vs. Tg3 - 1764_S1	-62.57	-126.3 to 1.168	No	ns	0.0563
WT - PBS vs. Tg3 - 112_S1	-36.95	-101.9 to 27.95	No	ns	0.4484
Tg3 - PBS vs. Tg3 - 1764_S1	-61.31	-123.9 to 1.233	No	ns	0.0568
Tg3 - PBS vs. Tg3 - 112_S1	-35.69	-99.43 to 28.04	No	ns	0.4630

Tg3 - 1764_S1 vs. Tg3 - 112_S1	25.62	-<br>38.12 to 89.35	No	ns	0.7200
23 weeks					
WT - PBS vs. Tg3 - PBS	48.60	-<br>18.53 to 115.7	No	ns	0.2380
WT - PBS vs. Tg3 - 1764 _S1	-62.72	-<br>127.7 to 2.219	No	ns	0.0623
WT - PBS vs. Tg3 - 112_ S1	0.8550	-<br>66.40 to 68.11	No	ns	>0.9999
Tg3 - PBS vs. Tg3 - 1764 _S1	-111.3	-176.1 to -46.51	Yes	***	0.0001
Tg3 - PBS vs. Tg3 - 112_ S1	-47.74	-<br>114.9 to 19.39	No	ns	0.2524
Tg3 - 1764_S1 vs. Tg3 - 112_S1	63.58	-<br>1.364 to 128.5	No	ns	0.0573

Supplementary Figure S1A

Dunnett's multiple comparisons test	Mean Diff.	95.00% CI of diff.	Below threshold?	Summary	Adjusted P Value
UNT vs. NTC	4.775	-23.41 to 32.96	No	ns	>0.9999
UNT vs. 4897	34.72	6.530 to 62.91	Yes	**	0.0071
UNT vs. 7219	-3.926	-32.11 to 24.26	No	ns	>0.9999
UNT vs. 10052	19.04	-9.146 to 47.23	No	ns	0.3996
UNT vs. 6151	17.90	-10.29 to 46.09	No	ns	0.4844
UNT vs. 3209	20.33	-7.856 to 48.52	No	ns	0.3151
UNT vs. 2618	29.31	1.126 to 57.50	Yes	*	0.0366
UNT vs. 9022	34.58	6.391 to 62.77	Yes	**	0.0075
UNT vs. 7352	37.74	9.549 to 65.93	Yes	**	0.0026
UNT vs. 10366	-12.91	-41.10 to 15.28	No	ns	0.8741
UNT vs. 8843	4.861	-23.33 to 33.05	No	ns	>0.9999
UNT vs. 8942	32.96	4.769 to 61.15	Yes	*	0.0124
UNT vs. 7227	7.686	-20.50 to 35.87	No	ns	0.9993
UNT vs. 1776	73.56	45.37 to 101.8	Yes	****	<0.0001
UNT vs. 1785	30.98	2.792 to 59.17	Yes	*	0.0226

UNT vs. 1759	76.49	48.30 to 104.7	Yes	****	<0.0001
UNT vs. 1123	48.14	19.95 to 76.33	Yes	****	<0.0001
UNT vs. 1787	20.90	-7.292 to 49.09	No	ns	0.2821
UNT vs. 1784	25.17	-3.016 to 53.36	No	ns	0.1091
UNT vs. 1786	14.70	-13.49 to 42.89	No	ns	0.7463
UNT vs. 1788	33.37	5.177 to 61.55	Yes	*	0.0110
UNT vs. 1758	66.96	38.77 to 95.15	Yes	****	<0.0001
UNT vs. 1669	12.27	-15.92 to 40.46	No	ns	0.9100
UNT vs. 1670	-0.1748	-28.36 to 28.01	No	ns	>0.9999
UNT vs. 1764	72.50	44.31 to 100.7	Yes	****	<0.0001

Supplementary Figure S1C

Dunnett's multiple comparisons test	Mean Diff.	95.00% CI of diff.	Below threshold?	Summary	Adjusted P Value
Isoform E1					
UNT vs. NTC	33.32	-7.330 to 73.98	No	ns	0.1839
UNT vs. 113	73.22	32.56 to 113.9	Yes	****	<0.0001
UNT vs. 128	38.31	-2.346 to 78.96	No	ns	0.0783
UNT vs. 131	29.75	-10.90 to 70.41	No	ns	0.3107
UNT vs. 146	47.01	6.358 to 87.67	Yes	*	0.0129
UNT vs. 147	31.01	-9.640 to 71.67	No	ns	0.2604
UNT vs. 174	26.79	-13.86 to 67.45	No	ns	0.4520
UNT vs. 179	29.62	-11.04 to 70.27	No	ns	0.3165
UNT vs. 181	47.62	6.966 to 88.27	Yes	*	0.0112
UNT vs. 213	23.42	-17.23 to 64.08	No	ns	0.6432
UNT vs. 214	21.50	-19.15 to 62.16	No	ns	0.7538
UNT vs. 233	28.60	-12.05 to 69.25	No	ns	0.3619
UNT vs. 234	-12.18	-52.83 to 28.48	No	ns	0.9986
UNT vs. 3514	27.94	-12.71 to 68.60	No	ns	0.3934
UNT vs. 3754	35.57	-5.084 to 76.22	No	ns	0.1273
UNT vs. 4431	20.21	-20.45 to 60.86	No	ns	0.8223

UNT vs. 5875	42.90	2.246 to 83.55	Yes	*	0.0317
UNT vs. 5876	20.21	-20.45 to 60.86	No	ns	0.8222
UNT vs. 5877	24.69	-15.96 to 65.35	No	ns	0.5689
UNT vs. 6278	22.53	-18.12 to 63.18	No	ns	0.6952
UNT vs. 7175	59.86	19.20 to 100.5	Yes	***	0.0005
UNT vs. 7237	43.15	2.492 to 83.80	Yes	*	0.0301
UNT vs. 8813	35.34	-5.314 to 75.99	No	ns	0.1324
UNT vs. 8893	28.49	-12.17 to 69.14	No	ns	0.3673
UNT vs. 9967	19.47	-21.19 to 60.12	No	ns	0.8575
Isoform E2					
UNT vs. NTC	-7.273	-47.93 to 33.38	No	ns	>0.9999
UNT vs. 113	39.05	-1.600 to 79.71	No	ns	0.0681
UNT vs. 128	32.72	-7.936 to 73.37	No	ns	0.2021
UNT vs. 131	16.43	-24.23 to 57.08	No	ns	0.9600
UNT vs. 146	41.13	0.4725 to 81.78	Yes	*	0.0455
UNT vs. 147	24.18	-16.48 to 64.83	No	ns	0.5989
UNT vs. 174	-0.6553	-41.31 to 40.00	No	ns	>0.9999
UNT vs. 179	11.43	-29.23 to 52.08	No	ns	0.9994
UNT vs. 181	-26.71	-67.36 to 13.94	No	ns	0.4565
UNT vs. 213	-2.335	-42.99 to 38.32	No	ns	>0.9999
UNT vs. 214	-9.659	-50.31 to 31.00	No	ns	>0.9999
UNT vs. 233	-15.63	-56.29 to 25.02	No	ns	0.9747
UNT vs. 234	-38.34	-79.00 to 2.313	No	ns	0.0778
UNT vs. 3514	7.658	-33.00 to 48.31	No	ns	>0.9999
UNT vs. 3754	13.03	-27.63 to 53.68	No	ns	0.9967
UNT vs. 4431	2.229	-38.43 to 42.88	No	ns	>0.9999
UNT vs. 5875	16.22	-24.43 to 56.88	No	ns	0.9642
UNT vs. 5876	3.868	-36.79 to 44.52	No	ns	>0.9999
UNT vs. 5877	8.403	-32.25 to 49.06	No	ns	>0.9999
UNT vs. 6278	23.97	-16.69 to 64.62	No	ns	0.6111

UNT vs. 7175	35.35	-5.309 to 76.00	No	ns	0.1323
UNT vs. 7237	30.79	-9.869 to 71.44	No	ns	0.2691
UNT vs. 8813	29.54	-11.12 to 70.19	No	ns	0.3198
UNT vs. 8893	7.840	-32.81 to 48.49	No	ns	>0.9999
UNT vs. 9967	-3.827	-44.48 to 36.83	No	ns	>0.9999

Supplementary Figure S2F

Šídák's multiple comparisons test	Mean diff.	95.00% CI of diff.	Below threshold?	Summary	Adjusted P Value
NTC - 1764_S1					
PF Cortex	40.32	20.39 to 60.25	Yes	****	<0.0001
Thalamus	28.76	8.826 to 48.69	Yes	**	0.0016
Hypothalamus	25.49	5.560 to 45.42	Yes	**	0.0061
Midbrain	15.22	-4.706 to 35.15	No	ns	0.2147
Hindbrain	18.35	-1.575 to 38.28	No	ns	0.0846

Source Data for Supplementary Figure S3 - Wes

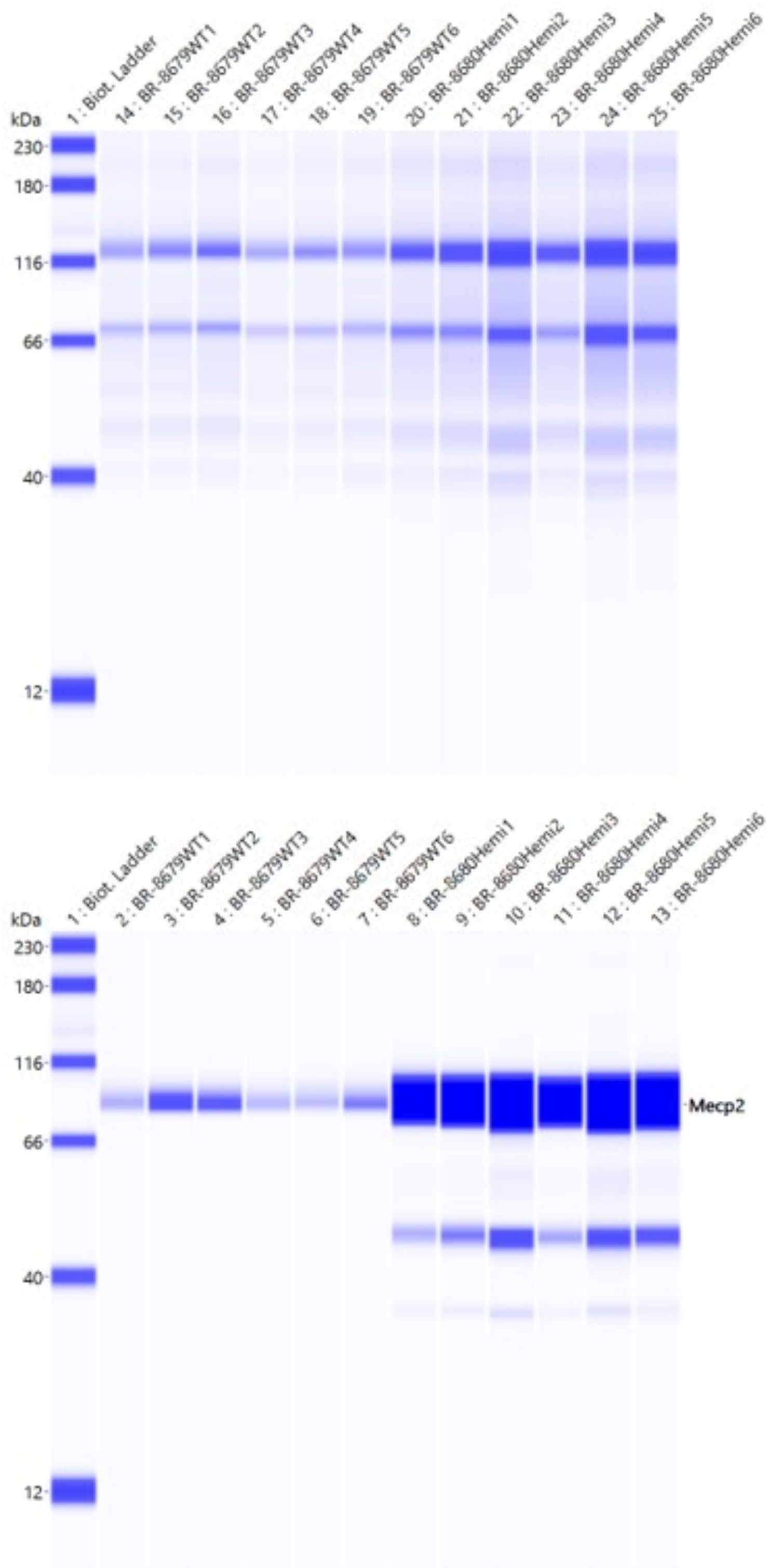

Source data for supplementary figure S3. Uncropped Wes images of total protein (top) used for normalization of Mecp2 bands (bottom).
