# Supplementary material for: Single-dose administration of therapeutic divalent siRNA targeting MECP2 prevents lethality in an MECP2 duplication mouse model
Source: Nat Commun. 2026 Jul 21;17:8877. doi: 10.1038/s41467-026-75611-7 (PMC13500697; doi:10.1038/s41467-026-75611-7)
Supplement: Supplementary file 3 [file 41467_2026_75611_MOESM3_ESM.pdf]

nature portfolio

Peer Review File

Single-dose administration of therapeutic divalent siRNA targeting MECP2 prevents lethality in an MECP2 duplication mouse model

Corresponding Author: Dr Vignesh Narayan Hariharan

This file contains all reviewer reports in order by version, followed by all author rebuttals in order by version.

Version 0:

Reviewer comments:

Reviewer #1

(Remarks to the Author)
The manuscript NCOMMS-25-29417-T, entitled " Single-dose administration of therapeutic divalent siRNA targeting MECP2 prevents
lethality for one year in an MECP2 duplication mouse model" by V Hariharan et al reports on the development of a novel siRNA to treat MECP2 duplication syndrome (MDS). The manuscript presents the screening, characterization and initial treatment results using cell lines and the two Tg Mouse models previously developed by the Zhogbi lab. The paper is complete in its screening and initial evaluation of these novel siRNA with modified backbones for this class of molecules. While generally well done there are issues that need to be addressed for this work.
Major issues:
1) The protein quantification of MECP2. The choice of immunoflorescence for quantification is unusual. Why did they not do western or other form of quantification. Then for the immuno- there should not be a reduction in percent of nuclei but rather a decrease in the intensity of all the nuclei. The reduction in RNA is n% in all cells so all cells should have a reduction in intensity- so there is no explanation why 70% of nuclei loose expression totally and 30% are still as bright. Even if the siRNA only gets in 70% of cells, they should just get dimmer, and not disappear. If the issue is there is a threshold for the immunoflorescene then some level doing spatial RNA sequencing to look at cellular level changes to show that there is cell to cell variability to explain the protein changes would be needed. The other issue with the immuno images is that MECP2 should be in all cells- just brighter in neurons. They chose to co-stain with DAPI- so having staining with NeuN, GFAP, and MBP to show which cells if any there is specificity for should occur.

2) The presentation of the methods, both in the main manuscript and the supplementary methods is not very descriptive and hard for the reader to know what exactly was done. This is across all of the methods for development of the siRNA through all the measurements.

3) The authors find that their levels of expression of mecp2 in the Tg and Tg3 mice are different than the original papers and that the behavioral findings and survival are also different. The explainations in the results and the discussion are not very comprehensive. The first Tg paper they describe retinal toxicity in the pure FVB line and then they use hybrid strains for the other testing. It would benefit this manuscript to have a clearer understanding of the differences between the lines derived at Jax labs and the orginal lines. Crossing these out to Bl6 or other work to sort this out would help.

4) A better understanding of this difference in knock down of the E1 isoform vs total Mecp2 is needed. The authors give some general and not specific explaintions for the contradictory findings. They also report that knock down in the WT does not lead to symptoms, but then propose that the over knockdown in the mutant could lead to symptoms. This does not fit together. This needs better explaination.

5) The choice of PBS as control and not the S1 backbone or the NTC is not sufficient. While the cells the PBS and NTC are the same as they showed, issues of off target effects, dosing, ect.. require that the control for the behavioral experiments be the NTC or backbone not PBS. If this is what they did (then the methods need to be improved- see above) but if it is not, to be thorough and better preclinical data- the controls should be the NTC.

Minor
1) The logic presented at the onset of the paper- to knock down MECP2 isoforms as the way to go forward then the experiments that present the complete knockdown followed by the isoform specific KO does not follow. If the isoforms are important, why not just do the isoform specific siRNAs and not do the total knockdown siRNA. Some clarification of the logic of the work is needed.

2) The conclusions on Page 18 results about the differences between the 1764 and 112 is not written very clearly- hard to parse what the authors are saying.

Reviewer #2

(Remarks to the Author)
This paper, “single dose administration of therapeutic divalent siRNA targeting MECP2 prevents lethality for one year in an MECP2 duplication model” by Hariharan et al. Descripbes the design and fabrication of isoform specific siRNA derivatives that are able to reduce the transcription and translation of MECP2. This is relevant, as duplication of the gene results in a severe neurodevelopmental disorder and reduction of the excess expression may benefit patients. The resulst of the study are encouraging in a sense that a long term reduction of the MECP2 expression could be observed in the control mice. When tested in humanized isease models, producing a 2 and 5 fold overexpression of the MECP2 protein, a similar reduction in MECP2 exptression was noticed, but the results of the behaare somewhat less interpretable due to a lack of a clear phenotype in the disease models. The poor phenotypic expression could not be foreseen, as earlies studies reported a much more severe phenotype of exactly the same mouse models. Nevertheless, the results show some abnomalities in the Tg1 (2x overexpression) model, perhaps related tot he MECP2 deletion syndrome Rett. In the Tg3 model that normally die prematurely, the life-span of the mice is restored to wild-type levels.

The results are relevant and nicely presented from a technical point of view. At points, I feel the biological relevance of the results could be clarified some more. Some suggesteions your will find below.

Comments:

Introduction:

The authors mention Rett and MECP2 duplication syndrome, but the relation between those could be clarified. At least, it should state that Rett is a female disorder due to absense of any functional MECP2 on one allele. In addition, some mutations in males couse a form of non-specific intellectual disabilty (for instance but not limited to the A140V mutations). The for instance do not mention this is a male-specific (well, mostly) synrome and of course X-linked. The term “mosaic” at l42+48 needs some clarification, e.g., “mosaic due to X-inactivation in females”

The discussion on the E1/E2 isoforms could at least mention that most MDS patients are due to a duplication of a large genomic fragment and so have both isoforms in excess.

Are the Tg1/3 mouse models also X-linked? This is clearly suggested, but a reference confirming (the provided reference suggest this only) X-linkage would be helpful.

Results

Are the Tg1/3 mouse models also X-linked? This is clearly suggested, but a reference confirming X-linkage would be helpful.

The Tg1 model. The expression of the MECP2 gene is reduced and some adverse effect on the behavior is notices. As this is likely due tot he reduced expression, one would expect the suppressed model to behave in the direction of a Rett mouse model. Is this indeed the case? This is not clearly stated.

Reviewer #3

(Remarks to the Author)
The manuscript from Dr. Hariharan and colleagues outlines a promising novel RNA silencing (siRNA)-based therapy for the treatment of MECP2 duplication syndrome (MDS), a rare X-linked neurodevelopmental disorder, for which no cure currently exists. Nucleic acid-based therapeutics (NATs) are being developed to silence MeCP2, with the antisense oligonucleotide (ASO) ION440 just entering clinical trial. As MECP2 is a dose-sensitive gene whose silencing lead to Rett Syndrome, the major challenge of RNA interfering techniques is the need to precisely control MECP2 titration. This is further complicated by variability in MECP2 levels among patients, and by the need to achieve long-term efficacy after a single therapeutic administration, as these therapies are delivered intrathecally. The Authors here propose a novel NAT strategy based on a class of fully chemically modified siRNA that are particularly suited to achieve a more controlled and durable gene silencing effect. They further hypothesize that targeting both MECP2 isoforms or selectively targeting the brain-prevalent e1 isoform may represent personalized strategies tailored to address the most severe and milder cases, respectively. After rounds of screening of different siRNA constructs, the Authors select two compounds (isoform and non-isoform-selective) with cross-

species homology and in vivo durable MECP2 silencing effects, to be tested for therapeutic efficacy in MDS animal models.

This study is highly innovative and has the potential to deliver a powerful therapeutic approach for the treatment of a severe and uncurable disease. In fact, the development of a siRNA therapeutic strategy with extended efficacy after a single ICV injection and cross-species homology may offer a rapid path to clinical testing. However, the main conclusions drawn by the Authors in the manuscript are not fully supported by the presented results. This includes the claim that "a single dose of the total isoform-silencing siRNA fully rescues early mortality and behavioral impairment", since only some of the behavioral/physiological alterations presented by a model of severe MDS were found to be improved. Furthermore, it is stated that "the isoform-selective targeting strategy may be safer in mild cases of MDS where exaggerated pharmacology may lead to Rett Syndrome". Results obtained treating a model of the milder MDS form with the isoform-selective targeting strategy are uninterpretable due to the absence of the expected phenotype in the mouse model and the lack of treatment effects. Further studies are thus needed to support this hypothesis. The Author also claim that their siRNA has extended effect duration compared to ION440, showing MECP2 silencing for 6-8 weeks following a single injection– however, they have limited the evaluation of the long term (4-month) efficacy to wt animals only. In the absence of studies demonstrating that a long term effects on MECP2 expression can be achieved in the MDS model, this claim does not appear to be supported. All these limitations should be properly addressed or the emphasis strongly dampened to make the manuscript ready for publication.

Another major concern is related to the notably milder behavioural phenotype exhibited by the two MDS models used in this study, despite elevated levels of transgene expression (which indeed contrast with previously reported findings in the literature). As mentioned above, the absence of a disease phenotype in the mouse model of milder MDS prevents drawing definitive conclusions about the efficacy of siRNAs, and claims regarding the potential toxicity of the non-selective siRNA appears overstated. Further investigations should be undertaken to clarify the differences from previous studies and the interpretation of the present findings.

Importantly, the work also suffers from major flaws in experimental design that must be addressed to meet publication standards. First of all, the lack of a comparison to wt controls hampers the possibility to evaluate whether the siRNA treatment restores physiological levels of MECP2, rather than simply being effective in reducing both mouse and human gene expression. The Authors should compare MECP2 protein levels in treated and untreated MDS mice to those in wt, to demonstrate the rescue at the molecular level and allow the reader to appreciate the therapeutic value of the proposed treatments.

The in vivo experimental design lacks coherence. In fact, mice of different ages and sexes and varying treatment strategies (e.g. the length and the age at which the treatments are initiated) have been used across experiments, preventing a clear comparison and interpretation. The Authors should more clearly justify their experimental choices and provide evidence of both molecular and behavioral rescue in models of comparable sex and age.

Methodological details are scarce for in vivo experiments. For example, lighting conditions during behavioral tests is not reported, and no details are provided on how the nest-site score was evaluated, and the scoring criteria for the 5-point Likert scale are not explained. The authors state that animals were housed 3–5 per cage, yet also mention that males were individually housed due to increased aggression. More details should be provided regarding the extent of this phenomenon and the timing of isolation within the experimental timeline, as social isolation can significantly impact behavioral outcomes.

Details on the statistical analyses should be included in the Methods section. Additionally, the statistical approach should be reviewed and post hoc comparisons re -run, since Dunnett's test is not appropriate for comparing a single group against multiple control groups.

Please also consider to more carefully justify the decision to assess mortality. In addition to the significant ethical implications involved, this choice hampered the possibility to confirm the rescue of MECP2 overexpression in the brain of treated mice. This confounds the interpretation of results, especially the lack of effects exerted by the e1 isoform-selective silencing. Please also provide details on the humane endpoints used in the survival experiments.

Reviewer #4

(Remarks to the Author)
I co-reviewed this manuscript with one of the reviewers who provided the listed reports. This is part of the Nature Communications initiative to facilitate training in peer review and to provide appropriate recognition for Early Career Researchers who co-review manuscripts.

Version 1:

Reviewer comments:

Reviewer #1

(Remarks to the Author)

The authors provided thoughtful responses to the reviews and made appropate text and figure changes that have approved the manuscript.

Reviewer #2

(Remarks to the Author)
The authors have nicely incorporated the suggestions of the reviewers, I have no further comments

Reviewer #3

(Remarks to the Author)
Although we still believe that the therapeutic strategy proposed by Dr. Hariharan and colleagues may have an important impact for the treatment of MDS, a severe and yet incurable disease, The Authors failed to convincingly address the multiple issues raised in our previous revision, thereby reducing confidence in the robustness of the results.

1. Comparing the degree of treatment-induced MeCP2 silencing in Tg3 mice to basal MeCP2 levels in wt mice is detrimental for interpreting phenotypic rescue, and as such do not appear to fall outside the scope of the present manuscript. Indeed, we are not suggesting to run a dose-response experiment to find an optimal dosage for the proposed treatment.

2. The performed experiments appear insufficient to address MECP2 isoform-specific contribution to MDS pathology. Although we agree that testing MECP2-E1 specific siRNA might be a valuable strategy to gain insight into this issue, the Authors only tested the behavioural effects of treatment with the isoform-specific 112_S1 siRNA in the Tg3 mouse model of disease. The degree of silencing of the e1 isoform in the Tg3 model upon treatment should be provided, to allow its relative contribution to the disease phenotype to be fairly judged.

3. Comparisons with ION440 should be maintained to explicitly address the specific theoretical and practical advantages of pursuing the development of this siRNA-based strategy relative to a nucleic acid-based therapeutics which is already in clinical trial, and underscore the innovation of the present work.

4. Although the Authors claim to have followed guidance to maximize the impact of animal use, they in fact appear to have employed an excessive number of animals and unnecessarily severe procedures, while failing to perform key analyses on the same animals that could have yielded a much stronger proof of concept. For example, safety controls at early ages appear premature at this stage, as an effective dose has not yet been established. Also, the Authors did not justify, as requested at point 9 of our previous comments, their decision to assess mortality. As this hampered the possibility to evaluate gene silencing in disease models while increasing animal suffering, the relevance of such measurements should be carefully justified.

5. The Authors affirm that they isolated animals at age 12 weeks, which lies in the middle of behavioral testing (11 weeks: nest building, 12 weeks: open field, 13 weeks: rotarod). As isolation is a stressful procedure that requires behavoral adaptation, additional details on the distance from behavioral testing is required for proper interpretation of the results. Also, further explanation on the scoring rules adopted for the evaluation of “nestlet manipulation” and “nestlet site” are needed, as according to the description in the supplemental section they appear to overlap, whereas the final scores in fig 4D are slightly different. Please also provide a reference for the cited Nature 2006 article as the link do not appar to work.

6. It is still odd that the phenotype of Tg3 mice appear so dampened despite the huge increase in MECP2 overexpression (8 fold, instead of 3-5 fold) relative to existing literature on the same model. The Authors should at least comment on this.

7. Sentences such as the one at page 13 line 341-42 (“These levels across all four brain regions were close to ~50% of NTC controls, which we consider a tolerable yet disease-modifying level of efficacy”) are inappropriate and should be revised, especially considering that Tg3 mice were found to display a ~8.4-fold increase in MeCP2 expression compared to wt controls.

8. The Authors should also explain how they choose to the specific tested dose.

9. Why didn’t the Authors perform comparisons with wt mice for survival and body weight measurements?

10. The Authors should further revise some statements throughout the text to more precisely reflect results. Some examples are:
a. “total MECP2 modulation through a single treatment with 1764_S1 at six weeks of age led to significant neurobehavioral phenotype recovery and completely reversed the premature mortality” - neurological measures were not performed on disease model mice treated at 6 weeks of age.
b. “Selective modulation of MECP2 E1 improved phenotypes initially” - the only phenotype significantly improved in the first 4 months is body weight, as other behavioural measures did not significantly differ from untreated Tg3 mice.
c. The Authors refer to a “1 year study period” while they evaluate treatment effect at 40 weeks maximum.
d. “isoform-selective silencing using 112_S1 provided initial gains in weight and behavioral trends in Tg3 but lost efficacy after 19 weeks, despite multi-month durability in wildtype mice.” – the latest effects in wt mice were assessed at 22 weeks.

11. In general, the manuscript should be revised with more attention. For instance, since the experiments on Tg1 mice have

been removed, all corresponding statements should likewise be deleted. Few examples are below:
a. One Sentence Summary: “Therapeutic siRNAs provide safe and durable modulation of MECP2 for the treatment of mild and severe MECP2 Duplication Syndrome”
b. Abstract: “Overall, this study introduces a series of preclinical candidates with the capacity to address the varying levels of MECP2 duplication encountered in clinical settings”
c. Results: Page 17, line 416-417 “we turned to a more severe MDS model, the Tg3 mice”

Reviewer #4

(Remarks to the Author)
I co-reviewed this manuscript with one of the reviewers who provided the listed reports. This is part of the Nature Communications initiative to facilitate training in peer review and to provide appropriate recognition for Early Career Researchers who co-review manuscripts.

Version 2:

Reviewer comments:

Reviewer #3

(Remarks to the Author)
While I do not consider completely solved some concerns of mine, the authors have now better explained the reasons for not addressing them. Most importantly, they have also highlighted these relevant limitations of the study within the manuscript and dampened the emphasis, when not justified. These changes to the text increase the reliability of the reported results and better direct the potential impact within the field of investigation. In its current form, I thus consider the manuscript acceptable for publication. Please note that “completely modifies” should be replaced, since the results do not support this interpretation.

Reviewer #4

(Remarks to the Author)
I co-reviewed this manuscript with one of the reviewers who provided the listed reports. This is part of the Nature Communications initiative to facilitate training in peer review and to provide appropriate recognition for Early Career Researchers who co-review manuscripts.

Open Access This Peer Review File is licensed under a Creative Commons Attribution 4.0 International License, which permits use, sharing, adaptation, distribution and reproduction in any medium or format, as long as you give appropriate credit to the original author(s) and the source, provide a link to the Creative Commons license, and indicate if changes were made.
In cases where reviewers are anonymous, credit should be given to 'Anonymous Referee' and the source.
The images or other third party material in this Peer Review File are included in the article’s Creative Commons license, unless indicated otherwise in a credit line to the material. If material is not included in the article’s Creative Commons

license and your intended use is not permitted by statutory regulation or exceeds the permitted use, you will need to obtain permission directly from the copyright holder.
To view a copy of this license, visit https://creativecommons.org/licenses/by/4.0/

REVIEWER COMMENTS

Reviewer #1 (Remarks to the Author):

The manuscript NCOMMS-25-29417-T, entitled " Single-dose administration of therapeutic divalent siRNA targeting MECP2 prevents
lethality for one year in an MECP2 duplication mouse model" by V Hariharan et al reports on the development of a novel siRNA to treat MECP2 duplication syndrome (MDS). The manuscript presents the screening, characterization and initial treatment results using cell lines and the two Tg Mouse models previously developed by the Zhogbi lab. The paper is complete in its screening and initial evaluation of these novel siRNA with modified backbones for this class of molecules. While generally well done there are issues that need to be addressed for this work.
Major issues:
1) The protein quantification of MECP2. The choice of immunoflorescence for quantification is unusual. Why did they not do western or other form of quantification. Then for the immuno- there should not be a reduction in percent of nuclei but rather a decrease in the intensity of all the nuclei. The reduction in RNA is n% in all cells so all cells should have a reduction in intensity- so there is no explanation why 70% of nuclei loose expression totally and 30% are still as bright. Even if the siRNA only gets in 70% of cells, they should just get dimmer, and not disappear. If the issue is there is a threshold for the immunoflorescene then some level doing spatial RNA sequencing to look at cellular level changes to show that there is cell to cell variability to explain the protein changes would be needed. The other issue with the immuno images is that MECP2 should be in all cells- just brighter in neurons. They chose to co-stain with DAPI- so having staining with NeuN, GFAP, and MBP to show which cells if any there is specificity for should occur.

We thank the reviewer for this insightful comment and agree that the choice of plotting % positivity is less relevant than plotting the relative fluorescence intensity in each cell. We thus have now substituted the original graphs with the data displaying cumulative relative intensity (expressed as a percentage of NTC control treated animal group) These graphs have been replaced in the manuscript (Figure 3F and Supplementary figure S2F) and are shown below for convenience.

We chose immunofluorescence over western blotting as it allowed us to measure the protein silencing in multiple regions of the brain with greater precision, we found it difficult to obtain the same spatial resolution accurately using western blots.

Figure 3F

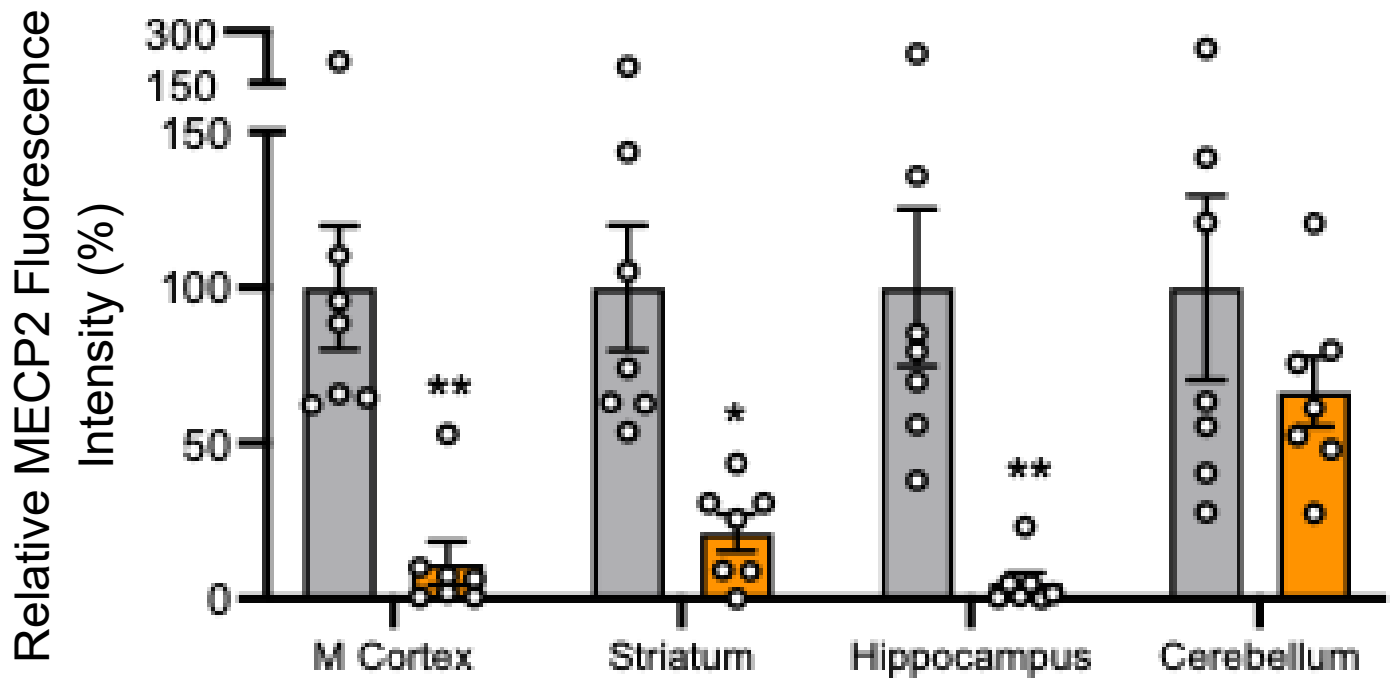

Supp Figure S2F

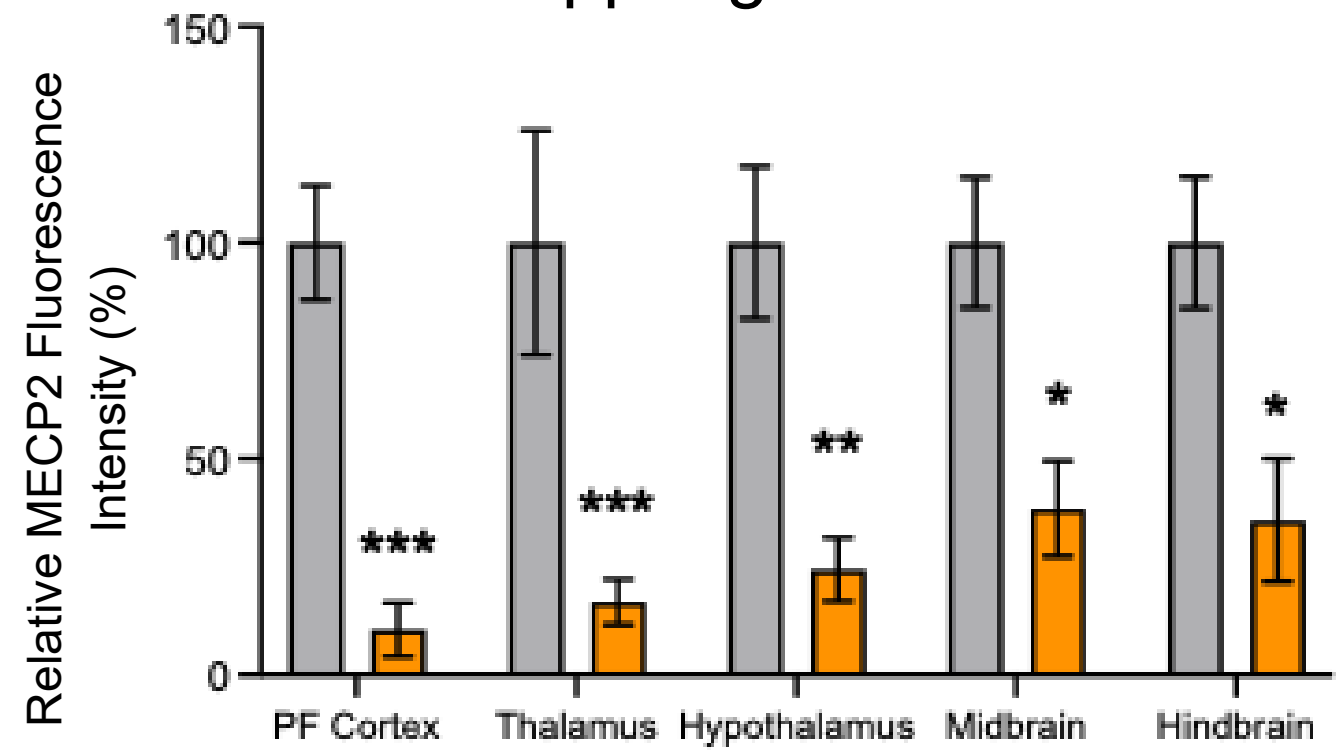

The following changes have been made to the manuscript text (in red font) lines 368:

For each region of interest, the integrated density of MECP2 immunofluorescence signal was evaluated and normalized to the number of cells in the view, defined by the number of dapi stained nuclei. On average, data for 500 to 1,000 cell per region were collected per animal.

MECP2 protein was significantly lower in 1764_S1-treated mice compared to NTC controls across most regions measured. The relative MECP2 abundance for each region, expressed as a percentage of NTC, was as follows: ~10% ($p < 0.001$) in the prefrontal cortex, ~21% ($p < 0.001$) in the striatum, ~11% ($p < 0.001$) in the medial cortex, ~5% ($p < 0.0001$) in the hippocampus, ~17% ($p < 0.001$) in the thalamus, ~24% ($p < 0.05$) in the hypothalamus, ~38% ($p = 0.06$) in the midbrain, ~36% ($p < 0.05$) in the hindbrain, and ~66% ($p = 0.7$) in the cerebellum (Figure 1F and Supplemental Figure S2F).

The question of cellular specificity of the expression and silencing is important and need to be discussed. We have previously characterized the divalent siRNA distribution and demonstrated uniformity in mouse brain distribution including efficient delivery to neurons, astrocytes and microglia,, labeled using NeuN, GFAP and IBA1 cellular markers. The divalent siRNA scaffold efficiently accumulates in both neurons and glial cells [1,2]. Using targets preferentially expressed only in neurons and glia (for example HTT and ApoE), we demonstrated silencing below level of detection, indicating that observed cellular accumulation is functionally active. The text of the manuscript has been changed to address this important concern. (line 380):

As observed in adult mice, 1764_S1 effectively reduced total MECP2 mRNA and protein levels in multiple brain regions of Tg3 neonatal mice. The divalent siRNA efficiently distributes to both neuronal and glia cells and can produce measurable effects, silencing neuron and glia selectively expressed genes below level of detection as shown in previous studies. [1,2] While MECP2 level of

expression was higher in neurons than glia, we expect the treatment with Di-valent siRNA to be effective in silencing MECP2 in all cell types.

2) The presentation of the methods, both in the main manuscript and the supplementary methods is not very descriptive and hard for the reader to know what exactly was done. This is across all of the methods for development of the siRNA through all the measurements.

We fully agree with this criticism and thank the reviewer for their comment. We have now expanded on our methods section, both in the main text and the supplementary file, to add more details and clarity. We have highlighted the changes in red throughout the methods sections. We have not added the edits here due to length constraints.

3) The authors find that their levels of expression of mecp2 in the Tg and Tg3 mice are different than the original papers and that the behavioral findings and survival are also different. The explainations in the results and the discussion are not very comprehensive. The first Tg paper they describe retinal toxicity in the pure FVB line and then they use hybrid strains for the other testing. It would benefit this manuscript to have a clearer understanding of the differences between the lines derived at Jax labs and the orginal lines. Crossing these out to Bl6 or other work to sort this out would help.

We agree with the reviewer that there are differences in the findings from MECP2 duplication mouse models (Tg1 and Tg3) between the original reports and our current study. Our work was performed at JAX using a colony rederived from frozen sperm. We had previously characterized survival in this line, and both our group and JAX were surprised to observe a much weaker phenotypic presentation. To rule out genotyping errors, we regenotyped all animals and confirmed the correct genotype.

To improve clarity and streamline the presentation for a broad audience journal such as *Nature Communications*, and in response to comments from multiple reviewers, we have revised the manuscript to focus solely on the Tg3 model and removed the Tg1 data. We are working with JAX to further investigate whether the method of genotype preservation (via sperm) could have contributed to the attenuation of phenotype—an effect recently described in detail for the widely used Alzheimer's 5xFAD model (ref).

Accordingly, we have removed the section describing Tg1 results and adjusted figures and text throughout to highlight the Tg3 data only. We believe that the Tg3 model adequately supports the key observations, and that removing Tg1 strengthens the clarity and focus of the manuscript without affecting the main conclusions.

Additionally, we have provided further details on the Tg3 strain and background as obtained from JAX in the manuscript as the reviewer suggests. These changes have been marked in red in the manuscript (line 321), and have been reproduced below for convenience:

In order to evaluate the efficacy of lead siRNAs and study their effect on disease phenotypes, we selected a well-characterized human transgenic MDS model developed by the Zoghbi lab[3]—FVB-Tg(MECP2)3Hzo/J (Tg3) obtained from the Jackson Laboratory and maintained on the FVB background (strain #008680), which expresses MECP2 at approximately three- to five-fold the wild-type level.

4) A better understanding of this difference in knock down of the E1 isoform vs total Mecp2 is needed. The authors give some general and not specific explaintions for the contradictory findings. They also report that knock down in the WT does not lead to symptoms, but then propose that the over knockdown in the mutant could lead to symptoms. This does not fit together. This needs better explaination.

We thank the reviewer for pointing out this deficiency in explanation. We have now added several lines of explanation to the introduction, results, and discussion sections to clarify the rationale of testing E1 vs total isoform silencing.

We have softened the language and removed the contradictory statements, as the study does evaluate the symptoms of over knockdown of MECP2 in WT mice. The changes we made ensure that the statements made in the manuscript are supported by the data generated therein.

All changes to the text are in red font, and have been reproduced for the reviewer's convenience below:

Lines 125: In this study, we developed fully chemically modified divalent siRNA therapeutic candidates capable of silencing total MECP2 or selectively MECP2 E1. Testing both E1 isoform selective and total MECP2 silencing will provide preliminary insights into the contribution of the E1 isoform to disease in this model and establish the ideal therapeutic target mRNA for the disease.

Lines 162: While studies have clearly shown that loss-of-function of the E1 isoform is necessary and sufficient to cause RTT in animals [4,5] and in humans[6-8], a similar understanding of the role of the E1 isoform in MDS is lacking. The two major MECP2 isoforms, E1 and E2, may have sufficient functional overlap to provide redundancy[9]. In healthy neurons, both isoforms are expressed at levels that maintain optimal total MECP2. In MECP2 duplication syndrome, where duplications typically span multiple genes, both E1 and E2 are overexpressed, resulting in elevated MECP2

levels. In order to evaluate whether MDS is driven by the E1 isoform alone, we also developed siRNA sequences that target isoform E1 but not E2.

Line 301: Since previous studies have shown that even reductions in MECP2 levels of 20-30% in wildtype mice result in anxiety-like phenotypes and other behavioral deficits that mimic some of the phenotypes of MECP2-null mice[10], behavioral analysis in WT mice should be conducted in future studies to fully understand the effect of the siRNAs developed here.

Line 487: In conclusion, total MECP2 modulation through a single treatment with 1764_S1 at six weeks of age led to significant neurobehavioral phenotype recovery and completely reversed the premature mortality observed in both control and isoform-selective treatment groups. Selective modulation of MECP2 E1 improved phenotypes initially, however effects diminished after four months without impacting overall survival. Future studies using a repeat dosing regimen are needed to evaluate if isoform E1 silencing alone could be sufficient to ameliorate MDS phenotypes and provide long term impact on disease phenotype.

Line 521: We developed small interfering RNAs (siRNAs) that target all isoforms of *MECP2* non-selectively, as well as siRNAs that selectively target the E1 isoform with minimal impact on E2 to identify the best therapeutic strategy.

Lines 532: This suggests that single-dose E1-selective silencing may not be as effective as total isoform silencing as a single dose. However, repeat dosing to maintain efficacy should be explored in future studies.

5) The choice of PBS as control and not the S1 backbone or the NTC is not sufficient. While the cells the PBS and NTC are the same as they showed, issues of off target effects, dosing, ect.. require that the control for the behavioral experiments be the NTC or backbone not PBS. If this is what they did (then the methods need to be improved- see above) but if it is not, to be thorough and better preclinical data- the controls should be the NTC.

We fully agree with the reviewer that NTC, and ideally both PBS and NTC together, are the best controls to include in all in vivo studies. Whenever possible, we follow this practice. For example, in our recently published long-term studies in Alzheimer's disease (5xFAD), ALS (G93A), and Huntington's disease (Q111), we included both PBS and NTC groups[2,11,12]. Across these studies, we observed no differences in phenotypes, biochemical readouts, or RNA-seq data, indicating that for this class of compounds there is minimal off-target activity in the CNS and no chemistry-related toxicity or pathology.

While this body of work provides supportive evidence, it is not an ideal substitute for including NTC directly in the present study. In this case, because the experiments were highly complex and conducted externally at JAX rather than in our lab, including an additional NTC group would have had significant cost implications that we were not able to accommodate. We prioritized conducting the study externally to increase the value and rigor of the dataset, particularly given the potential for these compounds to advance clinically.

We have revised the text to acknowledge this limitation and have added references to prior studies that directly compare PBS and NTC controls to further support the conclusion that the observed effects are target-specific rather than chemistry-related.

Line 554: An important caveat to the behavioral experiments conducted in this study is the usage of a vehicle control (PBS) instead of the conventional non-targeting control (NTC) siRNA of similar backbone chemistry but scrambled sequence. The choice of PBS control prevents the evaluation of the chemical and structural effects of the siRNA backbone alone on the behavior of MDS mice. Since the current study is a pharmacology and proof-of-concept study intended to support further pre-clinical and clinical development, we elected to use a PBS control to evaluate the lead compounds 'as a whole', including both sequence related (on-target) and chemical composition related (off-target) effects to mimic the study design typically used in pre-clinical drug development and in clinical trials. Ideally, utilizing both a PBS and NTC control would allow for assessing impact of sequence-related and chemical composition related effects, however mice availabilities and manageable cohort sizes limited this option. However, we have extensive historical control data across multiple mouse models (ALS, Alzheimer's, and Huntington's mouse models) showing that the divalent siRNA backbone and chemical structure of NTC does not affect disease [2,11,12]

Minor

1) The logic presented at the onset of the paper- to knock down MECP2 isoforms as the way to go forward then the experiments that present the complete knockdown followed by the isoform specific KO does not follow. If the isoforms are important, why not just do the isoform specific siRNAs and not do the total knockdown siRNA. Some clarification of the logic of the work is needed.

We thank the previewer for their comment. The logic has been re-written as suggested. Please see our response to Major comment #4.

2) The conclusions on Page 18 results about the differences between the 1764 and 112 is not written very clearly- hard to parse what the authors are saying.

This has been re-written to improve clarity. Please see edits to lines 479-481 in our response to Major comment #4.

Reviewer #2 (Remarks to the Author):

This paper, “single dose administration of therapeutic divalent siRNA targeting MECP2 prevents lethality for one year in an MECP2 duplication model” by Hariharan et al. Descripbes the design and fabrication of isoform specific siRNA derivatives that are able to reduce the transcription and translation of MECP2. This is relevant, as duplication of the gene results in a severe neurodevelopmental disorder and reduction of the excess expression may benefit patients. The resulst of the study are encouraging in a sense that a long term reduction of the MECP2 expression could be observed in the control mice. When tested in humanized isease models, producing a 2 and 5 fold overexpression of the MECP2 protein, a similar reduction in MECP2 exptression was noticed, but the results of the behaare somewhat less interpretable due to a lack of a clear phenotype in the disease models. The poor phenotypic expression could not be foreseen, as earlies studies reported a much more severe phenotype of exactly the same mouse models. Nevertheless, the results show some abnomalities in the Tg1 (2x overexpression) model, perhaps related tot he MECP2 deletion syndrome Rett. In the Tg3 model that normally die prematurely, the life-span of the mice is restored to wild-type levels.

The results are relevant and nicely presented from a technical point of view. At points, I feel the biological relevance of the results could be clarified some more. Some suggesteions your will find below.

Comments:

Introduction:

The authors mention Rett and MECP2 duplication syndrome, but the relation between those could be clarified. At least, it should state that Rett is a female disorder due to absense of any functional MECP2 on one allele. In addition, some mutations in males couse a form of non-specific intellectual disabilty (for instance but not limited to the A140V mutations). The for instance do not mention this is a male-specific (well, mostly) synrome and of course X-linked. The term “mosaic” at l42+48 needs some clarification, e.g., “mosaic due to X-inactivation in females”

We thank the reviewer for this valuable suggestion. We have added the following text in the introduction as per the suggestion:

Line 43: While both MDS and RTT are X-linked disorders, RTT is caused by heterozygous loss-of-function variants in MECP2 and predominantly affects females. In males, MECP2 loss-of-function is typically lethal due to the absence of a second X chromosome to provide functional MECP2, although rare cases of male RTT have been documented[13,14]. In contrast, MDS results from duplications of large regions of Xq28, including the MECP2 gene. MDS primarily affects males as most females usually show preferential inactivation of the X-chromosome carrying the duplication.

The discussion on the E1/E2 isoforms could at least mention that most MDS patients are due to a duplication of a large genomic fragment and so have both isoforms in excess.

We have added the following text as per the reviewer's suggestion:

Line 84-85: Due to the large regions of duplication, spanning several genes, MDS patients express all MECP2 isoforms in excess.

Are the Tg1/3 mouse models also X-linked? This is clearly suggested, but a reference confirming (the provided reference suggest this only) X-linkage would be helpful.

We thank the reviewer for this suggestion. We have now explicitly stated that the mouse model used in the study is also X-linked. The edits have been reproduced below –

Line 327-328: Additionally, the transgene is inserted on the X-chromosome, mimicking the X-linked presentation of the disease in humans.

Results

Are the Tg1/3 mouse models also X-linked? This is clearly suggested, but a reference confirming X-linkage would be helpful.

We thank the reviewer for this suggestion. We have now explicitly stated that the mouse model used in the study is also X-linked. The edits have been reproduced below –

Line 328: Additionally, the transgene is inserted on the X-chromosome, mimicking the X-linked presentation of the disease in humans.

The Tg1 model. The expression of the MECP2 gene is reduced and some adverse effect on the

behavior is notices. As this is likely due tot he reduced expression, one would expect the suppressed model to behave in the direction of a Rett mouse model. Is this indeed the case? This is not clearly stated.

We thank the reviewer for this comment. The questions regarding the lack of disease phenotype in Tg1 mice (compared to original publications) has been highlighted by other peer reviewers of this manuscript as a major drawback.

In order to maintain the quality of the results reported in this manuscript, and following suggestions of multiple reviewers we have decided to only present the results obtained using the Tg3 mouse since these results are more reliable. We have removed the section detailing results from the Tg1 model, as we continue to investigate the reasons for the discrepancy between this study and previously published studies.

Figures and text throughout the manuscript have been changed appropriately to describe results from Tg3 mice only.

Regarding the second question of whether Rett syndrome was induced in WT mice through MECP2 silencing, this has not been investigated appropriately in this manuscript. We have added the following clarifications in the text:

Lines 301: Since studies have shown that even reductions in MECP2 levels of 20-30% in wildtype mice result in anxiety-like phenotypes and other behavioral deficits that mimic some of the phenotypes of MECP2-null mice[10], behavioral analysis in WT mice should be conducted in future studies to fully understand the effect of the siRNAs developed here.

Reviewer #3 (Remarks to the Author):

The manuscript from Dr. Hariharan and colleagues outlines a promising novel RNA silencing (siRNA)-based therapy for the treatment of MECP2 duplication syndrome (MDS), a rare X-linked neurodevelopmental disorder, for which no cure currently exists. Nucleic acid-based therapeutics (NATs) are being developed to silence MeCP2, with the antisense oligonucleotide (ASO) ION440 just entering clinical trial. As MECP2 is a dose-sensitive gene whose silencing lead to Rett Syndrome, the major challenge of RNA interfering techniques is the need to precisely control MECP2 titration. This is further complicated by variability in MECP2 levels among patients, and by the need to achieve long-term efficacy after a single therapeutic administration, as these therapies are delivered intrathecally. The Authors here propose a novel NAT strategy based on a class of fully chemically modified siRNA that are particularly suited to achieve a more controlled and durable gene silencing effect. They further hypothesize that targeting both MECP2

isoforms or selectively targeting the brain-prevalent e1 isoform may represent personalized strategies tailored to address the most severe and milder cases, respectively. After rounds of screening of different siRNA constructs, the Authors select two compounds (isoform and non-isoform-selective) with cross-species homology and in vivo durable MECP2 silencing effects, to be tested for therapeutic efficacy in MDS animal models.

We thank the reviewer for their insightful review of this manuscript, and have addressed their concerns with appropriate edits to the manuscript text.

1. This study is highly innovative and has the potential to deliver a powerful therapeutic approach for the treatment of a severe and uncurable disease. In fact, the development of a siRNA therapeutic strategy with extended efficacy after a single ICV injection and cross-species homology may offer a rapid path to clinical testing. However, the main conclusions drawn by the Authors in the manuscript are not fully supported by the presented results. This includes the claim that "a single dose of the total isoform-silencing siRNA fully rescues early mortality and behavioral impairment", since only some of the behavioral/physiological alterations presented by a model of severe MDS were found to be improved.

We have softened the language around this claim to more accurately reflect the results of the study. We have made edits in several places to ensure that the conclusions are supported by the results as suggested by the reviewer. Edits (red font) made are:

Line 31: In this severe duplication model, a single dose of the total isoform-silencing siRNA rescued early mortality and select behavioral impairments.

Line 484: In conclusion, total MECP2 modulation through a single treatment with 1764_S1 at six weeks of age led to significant neurobehavioral phenotype recovery and completely reversed the premature mortality observed in both control and isoform-selective treatment groups. Selective modulation of MECP2 E1 improved phenotypes initially, however effects diminished after four months without impacting overall survival. Future studies using a repeat dosing regimen are needed to evaluate if isoform E1 silencing alone could be sufficient to ameliorate MDS phenotypes and provide long term impact on disease phenotype.

Line 523: In the severe MDS model (Tg3), a non-isoform selective siRNA (1764_S1) rescued weight loss, select behavioral deficits, and survival for the one-year study period, marking the first observed survival benefit in MDS therapy with a single dose. E1 isoform-selective silencing using 112_S1 provided initial gains in weight and behavioral trends in Tg3 but lost efficacy after 19 weeks, despite multi-month durability in wildtype mice. This suggests that single-dose E1-

selective silencing may not as effective as total isoform silencing as a single dose. However, repeat dosing to maintain efficacy should be explored in future studies.

2. Furthermore, it is stated that “the isoform-selective targeting strategy may be safer in mild cases of MDS where exaggerated pharmacology may lead to Rett Syndrome”. Results obtained treating a model of the milder MDS form with the isoform-selective targeting strategy are uninterpretable due to the absence of the expected phenotype in the mouse model and the lack of treatment effects. Further studies are thus needed to support this hypothesis.

We agree with the reviewer and have received similar feedback from other reviewers as well. We propose instead, an alternative value in testing the isoform E1 specific siRNA.

Furthermore, to maintain the quality of the results reported in this manuscript, we have decided to present the results obtained using the Tg3 mouse model only since these results are more reliable. We have removed the section detailing results from the Tg1 model, as we continue to investigate the reasons for the discrepancy between this study and published studies.

Figures and text throughout the manuscript have been changed appropriately to describe results from Tg3 mice only. The relevant changes to the manuscript have been reproduced below for convenience:

Lines 125: In this study, we developed fully chemically modified divalent siRNA therapeutic candidates capable of silencing total MECP2 or MECP2 E1 specifically. Testing both E1 isoform selective and total isoform silencing will provide preliminary insights into the contribution of the E1 isoform to the disease in this model.

Lines 162: While studies have clearly shown that loss-of-function of the E1 isoform is necessary and sufficient to cause RTT in animals [4,5] and in humans[6-8], a similar understanding of the role of the E1 isoform in MDS is lacking. The two major MECP2 isoforms, E1 and E2, may have sufficient functional overlap to provide redundancy[9]. In healthy neurons, both isoforms are expressed at levels that maintain optimal total MECP2. In MECP2 duplication syndrome, where duplications typically span multiple genes, both E1 and E2 are overexpressed, resulting in elevated MECP2 levels. In order to evaluate whether MDS is driven by the E1 isoform alone, we also developed siRNA sequences that target isoform E1 but not E2.

3. The Author also claim that their siRNA has extended effect duration compared to ION440, showing MECP2 silencing for 6-8 weeks following a single injection– however, they have limited the evaluation of the long term (4-month) efficacy to wt animals only. In the absence of studies demonstrating that a long term effects on MECP2 expression can be achieved in the MDS model, this claim does not appear to be supported. All these limitations should be properly addressed or the emphasis strongly dampened to make the manuscript ready for publication.

We acknowledge this limitation in the study and have removed comparisons with ION440 in the manuscript. Edits to the manuscript text are reproduced below for the reviewer's convenience:

Line 545: However, due to the absence of silencing information, it remains uncertain whether the phenotypic benefits of 1764_S1 persisted beyond the pharmacodynamic effect as reported with antisense oligonucleotides in MDS[15]. Future studies need to evaluate gene silencing in disease models for a better understanding of the duration of effect.

Line 563: The siRNAs reported here are promising due to their extended duration of effect with a single administration. Since nucleic acid therapeutics generally cannot cross the blood-brain barrier (BBB) without a BBB shuttle or specific scaffold[16-19], intrathecal (IT) or intracerebroventricular (ICV) administration is required, posing significant patient risks[20-23]. Therefore, durability is crucial for clinical utility and acceptance, particularly in the pediatric population. Additionally, divalent siRNAs show homogenous distribution and silencing in deep brain regions across multiple preclinical species[24-26], making them ideal investigational therapeutic candidates for MDS, which affects all brain regions.

4. Another major concern is related to the notably milder behavioural phenotype exhibited by the two MDS models used in this study, despite elevated levels of transgene expression (which indeed contrast with previously reported findings in the literature). As mentioned above, the absence of a disease phenotype in the mouse model of milder MDS prevents drawing definitive conclusions about the efficacy of siRNAs, and claims regarding the potential toxicity of the non-selective siRNA appears overstated. Further investigations should be undertaken to clarify the differences from previous studies and the interpretation of the present findings.

As mentioned in the response to a previous comment #2, we have removed the section detailing results from the Tg1 model, as we continue to investigate the reasons for the discrepancy between this study and published studies.

Figures and text throughout the manuscript have been changed appropriately to describe results from Tg3 mice only.

5. Importantly, the work also suffers from major flaws in experimental design that must be

addressed to meet publication standards. First of all, the lack of a comparison to wt controls hampers the possibility to evaluate whether the siRNA treatment restores physiological levels of MECP2, rather than simply being effective in reducing both mouse and human gene expression. The Authors should compare MECP2 protein levels in treated and untreated MDS mice to those in wt, to demonstrate the rescue at the molecular level and allow the reader to appreciate the therapeutic value of the proposed treatments.

We thank the reviewer for this comment and important suggestions. However, we believe that such a study is outside the scope of the current proof-of-concept manuscript as it would require an ascending dose study to find the optimal dose that reduces MECP2 to WT levels. We plan to run such study in the future, to gain a better understanding of the dose-PD relationship of these leads.

We therefore prioritized endpoints that could show disease (behavioral) modification as proof-of-concept and used a sufficiently high dose where we expect maximal silencing of MECP2.

6. The in vivo experimental design lacks coherence. In fact, mice of different ages and sexes and varying treatment strategies (e.g. the length and the age at which the treatments are initiated) have been used across experiments, preventing a clear comparison and interpretation. The Authors should more clearly justify their experimental choices and provide evidence of both molecular and behavioral rescue in models of comparable sex and age.

Our default treatment paradigm is to treat mice at 6 weeks of age and then follow phenotypic effects over the duration of the study. In the first study (Figure 2), we evaluated silencing at 1 and 4 months post-treatment. These are typical timepoints used in similar siRNA studies and allow us to assess and confirm the durability of the effect. The results demonstrate that silencing is maintained at ~80% for at least 4 months post-treatment. Because MECP2 is a widely expressed gene, this study was performed in wild-type animals. The same treatment paradigm was then applied in the major phenotypic survival study, where treatment was initiated at 6 weeks and behavioral changes were observed over time (Figure 4).

In general, when the siRNA target sequence is present, efficacy does not depend strongly on the mouse background. Thus, we expect similar silencing and durability in Tg3 animals as in wild-type. However, to be certain, we sought to confirm efficacy directly in the Tg3 model before committing to a large-scale behavioral study (Figure 3A). After reviving the colony, we first evaluated phenotype and survival. The animals were maintained at JAX, and by 30 weeks the colony began to decline. JAX contacted us to determine whether we wished to utilize the remaining animals. Following general guidance to maximize the impact of animal use, we decided to test siRNA activity in the Tg3 background using this cohort. Because the mice were older, already symptomatic, and limited in number, we terminated the study at one month. The only conclusion drawn from this experiment is independent confirmation that the compound batch

retained activity in the Tg3 background, thereby de-risking advancement to a larger behavioral and survival study.

Finally, the neonatal experiment (Figure 3C) was undertaken at the advice of collaborators to explore safety at very early ages, given that MDS is a pediatric condition. We have mentioned this in Line 344: Given that MDS is a pediatric condition where early intervention is presumably more beneficial, we also assessed whether safe levels of silencing could be achieved in the smaller brains of neonatal mice

We have revised the text to clarify why adult Tg3 mice at 30 weeks of age were used:

Line 330: We utilized an existing colony of Adult Tg3 mice (30 weeks of age) to assess the efficacy of the siRNAs.

7. Methodological details are scarce for in vivo experiments. For example, lighting conditions during behavioral tests is not reported, and no details are provided on how the nest-site score was evaluated, and the scoring criteria for the 5-point Likert scale are not explained. The authors state that animals were housed 3–5 per cage, yet also mention that males were individually housed due to increased aggression. More details should be provided regarding the extent of this phenomenon and the timing of isolation within the experimental timeline, as social isolation can significantly impact behavioral outcomes.
We thank the reviewer for this comment. We have included additional details to the methods section and in the supplementary data, detailing the animal housing conditions, recording of behavioral readouts and scoring systems.

8. Details on the statistical analyses should be included in the Methods section. Additionally, the statistical approach should be reviewed and post hoc comparisons re -run, since Dunnett's test is not appropriate for comparing a single group against multiple control groups.

We thank the reviewer for this comment. We have included additional details to the methods section and supplementary data, detailing the statistical analyses with appropriate tests as the reviewer suggested.

9. Please also consider to more carefully justify the decision to assess mortality. In addition to the significant ethical implications involved, this choice hampered the possibility to confirm the rescue of MECP2 overexpression in the brain of treated mice. This confounds the interpretation of results, especially the lack of effects exerted by the e1 isoform-selective silencing. Please also provide details on the humane endpoints used in the survival experiments.

We thank the reviewer for this comment. We have included additional details to the methods section.

Reviewer #4 (Remarks to the Author):

I co-reviewed this manuscript with one of the reviewers who provided the listed reports. This is part of the Nature Communications initiative to facilitate training in peer review and to provide appropriate recognition for Early Career Researchers who co-review manuscripts.

We thank the co-reviewer for their time and efforts in peer review.

1. Alterman, J.F., *et al.* A divalent siRNA chemical scaffold for potent and sustained modulation of gene expression throughout the central nervous system. *Nat Biotechnol* **37**, 884–894 (2019).
2. Ferguson, C.M., *et al.* Silencing Apoe with divalent-siRNAs improves amyloid burden and activates immune response pathways in Alzheimer's disease. *Alzheimers Dement* **20**, 2632–2652 (2024).
3. Collins, A.L., *et al.* Mild overexpression of MeCP2 causes a progressive neurological disorder in mice. *Hum Mol Genet* **13**, 2679–2689 (2004).
4. Itoh, M., *et al.* Methyl CpG-binding protein isoform MeCP2_e2 is dispensable for Rett syndrome phenotypes but essential for embryo viability and placenta development. *J Biol Chem* **287**, 13859–13867 (2012).
5. Yasui, D.H., *et al.* Mice with an isoform-ablating Mecp2 exon 1 mutation recapitulate the neurologic deficits of Rett syndrome. *Hum Mol Genet* **23**, 2447–2458 (2014).
6. Amir, R.E., *et al.* Mutations in exon 1 of MECP2 are a rare cause of Rett syndrome. *J Med Genet* **42**, e15 (2005).
7. Quenard, A., *et al.* Deleterious mutations in exon 1 of MECP2 in Rett syndrome. *Eur J Med Genet* **49**, 313–322 (2006).
8. Saunders, C.J., Minassian, B.E., Chow, E.W., Zhao, W. & Vincent, J.B. Novel exon 1 mutations in MECP2 implicate isoform MeCP2_e1 in classical Rett syndrome. *Am J Med Genet A* **149A**, 1019–1023 (2009).
9. Kerr, B., *et al.* Transgenic complementation of MeCP2 deficiency: phenotypic rescue of Mecp2-null mice by isoform-specific transgenes. *Eur J Hum Genet* **20**, 69–76 (2012).

10. Shao, Y., *et al.* Identification and characterization of conserved noncoding cis-regulatory elements that impact Mecp2 expression and neurological functions. *Genes Dev* **35**, 489–494 (2021).
11. Belgrad, J., *et al.* Blocking somatic repeat expansion and lowering huntingtin via RNA interference synergize to prevent Huntington's disease pathogenesis in mice. *bioRxiv* (2025).
12. Weiss, A., *et al.* RNAi-mediated silencing of SOD1 profoundly extends survival and functional outcomes in ALS mice. *Mol Ther* **33**, 3917–3938 (2025).
13. Zeev, B.B., *et al.* Rett syndrome: clinical manifestations in males with MECP2 mutations. *J Child Neurol* **17**, 20–24 (2002).
14. Chahil, G., Yelam, A. & Bollu, P.C. Rett Syndrome in Males: A Case Report and Review of Literature. *Cureus* **10**, e3414 (2018).
15. Shao, Y., *et al.* Antisense oligonucleotide therapy in a humanized mouse model of MECP2 duplication syndrome. *Sci Transl Med* **13**(2021).
16. Barker, S.J., *et al.* Targeting the transferrin receptor to transport antisense oligonucleotides across the mammalian blood-brain barrier. *Sci Transl Med* **16**, eadi2245 (2024).
17. Crawford, L., Rosch, J. & Putnam, D. Concepts, technologies, and practices for drug delivery past the blood-brain barrier to the central nervous system. *J Control Release* **240**, 251–266 (2016).
18. Geary, R.S., Norris, D., Yu, R. & Bennett, C.F. Pharmacokinetics, biodistribution and cell uptake of antisense oligonucleotides. *Adv Drug Deliv Rev* **87**, 46–51 (2015).
19. Nagata, T., *et al.* Cholesterol-functionalized DNA/RNA heteroduplexes cross the blood-brain barrier and knock down genes in the rodent CNS. *Nat Biotechnol* **39**, 1529–1536 (2021).
20. Delhaas, E.M. & Huygen, F. Complications associated with intrathecal drug delivery systems. *BJA Educ* **20**, 51–57 (2020).
21. Goel, V., *et al.* Adverse Events and Complications Associated With Intrathecal Drug Delivery Systems: Insights From the Manufacturer and User Facility Device Experience (MAUDE) Database. *Neuromodulation* **24**, 1181–1189 (2021).
22. Belov, V., *et al.* Large-Volume Intrathecal Administrations: Impact on CSF Pressure and Safety Implications. *Front Neurosci* **15**, 604197 (2021).
23. Fahoum, F. & Eyal, S. Intracerebroventricular administration for delivery of antiseizure therapeutics: Challenges and opportunities. *Epilepsia* **64**, 1750–1765 (2023).
24. Mazur, C., *et al.* Brain pharmacology of intrathecal antisense oligonucleotides revealed through multimodal imaging. *JCI Insight* **4**(2019).
25. Jafar-Nejad, P., *et al.* The atlas of RNase H antisense oligonucleotide distribution and activity in the CNS of rodents and non-human primates following central administration. *Nucleic Acids Res* **49**, 657–673 (2021).
26. Ferguson, C.M., *et al.* Comparative route of administration studies using therapeutic siRNAs show widespread gene modulation in Dorset sheep. *JCI Insight* **6**(2021).

P2 manuscript
REVIEWER COMMENTS

Reviewer #1 (Remarks to the Author):

The authors provided thoughtful responses to the reviews and made appropate text and figure changes that have approved the manuscript.

We thank the reviewer for their valuable time and feedback and are happy that we were able to address their comments satisfactorily.

Reviewer #2 (Remarks to the Author):

The authors have nicely incorporated the suggestions of the reviewers, I have no further comments

We thank the reviewer for their valuable time and feedback and are happy that we were able to address their comments satisfactorily.

Reviewer #3 (Remarks to the Author):

Although we still believe that the therapeutic strategy proposed by Dr. Hariharan and colleagues may have an important impact for the treatment of MDS, a severe and yet incurable disease, The Authors failed to convincingly address the multiple issues raised in our previous revision, thereby reducing confidence in the robustness of the results.

We thank the reviewer for their valuable time and feedback on this important manuscript. We believe that the manuscript findings are robust, supported by appropriately designed and sufficiently powered experiments to address specific questions. We address the reviewer's concerns below within the scope of this manuscript and look forward to their feedback.

1. Comparing the degree of treatment-induced MeCP2 silencing in Tg3 mice to basal MeCP2 levels in wt mice is detrimental for interpreting phenotypic rescue, and as such do not appear to fall outside the scope of the present manuscript. Indeed, we are not suggesting to run a dose-response experiment to find an optimal dosage for the proposed treatment.

We thank the reviewer for their feedback. In this manuscript, we show silencing of both human and mouse MECP2 at two different ages, using one of the lead siRNAs in Tg3 mice in Figure 3. Experiments in figure 3 were intended specifically to confirm silencing in the disease model using a lead compound. The results show that silencing is reproducible between WT mice and diseased mice. Even though only one siRNA (non-isoform selective 1764_S1) was tested, we observed similar efficacy of this siRNA in WT and Tg3 mice, supporting the translatable efficacy in this model.

Once we established activity of one of the lead siRNA in Tg3 mice, we went ahead to perform the phenotypic rescue and mortality study. We would like to emphasize that this study's design and focus was not to assess the effect of the siRNA on gene silencing, as this has been established in Figures 2 and Figure 3.

While gene silencing would have been a beneficial addition to the readouts, we prioritized behavior modification and time to death (which naturally precludes euthanasia and tissue collection at a pre-determined time point prior to natural mortality). We strongly believe the extension in mortality (strongly evident to up to 35 weeks post treatment) demonstrates for the first time that this disease is indeed controllable using the siRNA therapy developed in this work.

While we would love to correlate level of silencing to phenotypic rescue as the reviewers suggest, we work within the practical constraints of time and funding. At the moment, we are unable to perform additional experiments due to funding limitations. We believe for the scope of this manuscript, proof-of-concept has been established.

We have added text modifications that we detail in response to comment #2 below, acknowledging the limitations of the work.

2. The performed experiments appear insufficient to address MECP2 isoform-specific contribution to MDS pathology. Although we agree that testing MECP2-E1 specific siRNA might be a valuable strategy to gain insight into this issue, the Authors only tested the behavioural effects of treatment with the isoform-specific 112_S1 siRNA in the Tg3 mouse model of disease. The degree of silencing of the e1 isoform in the Tg3 model upon treatment should be provided, to allow its relative contribution to the disease phenotype to be fairly judged.

We thank the reviewer for their comments. As mentioned in response to the previous comment, the behavioral/survival focused experiment in figure 4 has no mRNA silencing collected as that was not the focus of the study. We achieved similar silencing levels for 1764 siRNA in both wild-type and disease mice (Figures 2 and 3). Assuming that the silencing levels will reproduce similarly for 112, we expect it to be less potent that 1764, leading to the potential lesser effect compared to 1764 siRNA. As stated in the response to the previous comment, the results from figure 3 show that silencing is reproducible between WT mice and diseased mice.

We understand that this is a limitation of the study, which we have addressed by modifying the concluding paragraph of the results section to acknowledge the these points raised by the reviewer in comments #1 and #2. The modified paragraph is reproduced below for convenience:

"In conclusion, total MECP2 modulation through a single treatment with 1764_S1 at six weeks of age led to some neurobehavioral phenotype recovery and rescued the premature mortality observed in both control and isoform-selective treatment groups. Selective modulation of MECP2 E1 improved phenotypes initially, however the effects diminished after four months without impacting overall survival. It is unclear whether the lower disease modifying efficacy of

112_S1 is due to the isoform specificity or due to differences in potency between 1764_S1 and 112_S1 in disease mice. Future studies would benefit from measuring the extent of gene silencing in the context of Tg3 mice to accurately correlate pharmacodynamic effect of siRNA with behavioral modification. While this study provides proof-of-concept for disease modification, the amount of target gene silencing required to provide disease modification remains an outstanding question. Future studies using a repeat dosing regimen with target gene silencing as an additional readout are needed to evaluate the extent of gene silencing required and if isoform E1 silencing alone could be sufficient to ameliorate MDS phenotypes and provide long term disease modification."

3. Comparisons with ION440 should be maintained to explicitly address the specific theoretical and practical advantages of pursuing the development of this siRNA-based strategy relative to nucleic acid-based therapeutics which is already in clinical trial, and underscore the innovation of the present work.

We thank the reviewer for their comment. We originally included comparisons to the ION440 program in the manuscript's initial submission, which we then proceeded to remove all of them per the request of the reviewer (due to the lack of data supporting the comparisons). To address both requests, we maintained the removal of all comparisons to ION440 throughout the manuscript but added a discussion on the theoretical and practical advantages of utilizing an siRNA therapeutic to that of an ASO in general. We have reproduced the newly added discussion below:

"The current clinical landscape for MDS is led by ION440, an antisense oligonucleotide (ASO) therapeutic in Phase 1-2 trials (Ionis Pharmaceuticals, NCT06014541). While experimental differences preclude a direct comparison between ION440 and the lead siRNA candidates presented here, these modalities offer complementary advantages. The catalytic nature of the RNA-induced silencing complex utilized by siRNAs allows for robust potency, a characteristic highlighted in previous comparative studies of oligonucleotide therapeutics[1]. ASOs, meanwhile, benefit from a longer history of clinical investigation, a substantial safety database, and dual-compartment activity in both the nucleus and cytoplasm[2]."

4. Although the Authors claim to have followed guidance to maximize the impact of animal use, they in fact appear to have employed an excessive number of animals and unnecessarily severe procedures, while failing to perform key analyses on the same animals that could have yielded a much stronger proof of concept. For example, safety controls at early ages appear premature at this stage, as an effective dose has not yet been established. Also, the Authors did not justify, as requested at point 9 of our previous comments, their decision to assess mortality. As this hampered the possibility to evaluate gene silencing in disease models while increasing animal suffering, the relevance of such measurements should be carefully justified.

We thank the reviewer for their feedback. While we do see the reviewer's point of view regarding the merits of assessing gene silencing in the disease model, we strongly believe that

choosing mortality is a stronger “black or white” evidence for proof-of-concept. Additionally, as mentioned in the previous response, we did confirm gene silencing efficacy of the siRNAs used in both wild type and diseased mice (Figures 2 and Figure 3). Regardless of the amount of gene silencing obtained, the extension of lifespan in mice expressing ~8x MECP2 in the brain is—in our opinion— the strongest proof-of-concept possible for the disease-modifying impact of the siRNA treatment.

With respect to point 9 of the reviewers’ previous comments on our decision to assess mortality, we would like to provide additional explanation. This project was funded by Rett syndrome foundation, which is a patient centric foundation funded by the generous donations of patients themselves. Input from the foundation emphasized the importance of running these studies at a CRO (JAX) to have external validation and robustness of the data on Mecp2 siRNA and its disease-modifying potential in an unbiased manner. Following discussion with JAX, and other clinicians involved in the work, in addition to limitations in terms of funding and animal availability, it was decided to go for the survival/mortality readout as a clearer measure and in accordance with the foundation’s interest.

We do not believe there was an ‘excessive’ number of animals used. The study was powered appropriately for a behavioral and mortality study, and it was conducted in accordance with institutional animal ethics protocols and carried out by trained and professional staff at JAX insuring no excessive suffering was present.

With respect to dose, we went with a dose that based on our previous experience with divalent siRNAs, would most likely have an impact on behavior and mortality.

5. The Authors affirm that they isolated animals at age 12 weeks, which lies in the middle of behavioral testing (11 weeks: nest building, 12 weeks: open field, 13 weeks: rotarod). As isolation is a stressful procedure that requires behavoral adaptation, additional details on the distance from behavioral testing is required for proper interpretation of the results.
Also, further explanation on the scoring rules adopted for the evaluation of “nestlet manipulation” and “nestlet site” are needed, as according to the description in the supplemental section they appear to overlap, whereas the final scores in fig 4D are slightly different. Please also provide a reference for the cited Nature 2006 article as the link do not appear to work.

Because the nest building assay requires isolation overnight, the male mice in the study were not regrouped, as we were concerned it would lead to aggression and further stress. The mice were separated for the nest building assay at least 7 days prior to the open field test. This has been clarified in the methods (reproduced below):

Animals were housed 3–5 per cage up until the first nest building assay at 11 weeks of age; at that age, all males were housed singly to prevent aggression, observed in previous studies following re-grouping mice. At least 7 days elapsed before the next behavioral test. Behavioral tests were all performed during the light cycle.
Mice were humanely euthanized at the end of the testing period for tissue analyses, or earlier if they reached a humane endpoint. Humane endpoint was met upon review by a Veterinarian if an untreatable condition developed. Conditions observed in this study were paraphimosis,

dehydration not responding to liquid gel supplementation in the cage, extreme agitation, sustained body weight loss, lameness or labored breathing. Age at humane endpoint was used for the survival analyses.

The nestlet manipulation and nestlet site are both part of the same scoring system. Because there is, by definition, no nest site to evaluate for scores of 0-1, it is classified as "manipulation of nestlet". Scores of 2-5 are referred to as "nest site" scoring, as they have a centralized nest. This has been clarified by creating Supplemental Table S2, reproduced below:

Supplementary Table S2. Nest building rating scale.

<table>
<tr><td colspan="2">Score</td><td>Definition</td></tr>
<tr><td rowspan="2">Nestlet Manipulation Score</td><td>0</td><td>Nestlet is untouched.</td></tr>
<tr><td>1</td><td>It is not clear where the nest is located in the cage (i.e., the nestlet material is spread throughout the cage).</td></tr>
<tr><td rowspan="4">Nest Site Score</td><td>2</td><td>The nest is flat with no shallow walls, centralized in one spot.</td></tr>
<tr><td>3</td><td>A slightly cupped shape where the walls of the nest are less than ½ the height of a dome that would cover a mouse.</td></tr>
<tr><td>4</td><td>Walls that are ½ the height of a dome that would cover a mouse.</td></tr>
<tr><td>5</td><td>Walls that are more than ½ the height of a dome that would cover a mouse, which may or may not fully enclose the nest.</td></tr>
</table>

Because the nest score is part of the manipulation score, and the manipulation score has a broader range (0-5), we agree with the suggestion of the reviewer and, for clarity and to prevent redundancy in the data, removed the nest site score from Figure 4.D (middle panel).

We have also added additional reference details for the Nature 2006 article. For clarity, the reference is as follows:
Deacon, R. Assessing nest building in mice. Nat Protoc 1, 1117–1119 (2006).
https://doi.org/10.1038/nprot.2006.170

6. It is still odd that the phenotype of Tg3 mice appear so dampened despite the huge increase in MECP2 overexpression (8 fold, instead of 3-5 fold) relative to existing literature on the same model. The Authors should at least comment on this.

We agree with the reviewer that this is an unexpected finding and have added a comment in the results section on this finding. We have added the reviewer's observation of 'huge increase in MECP2' to this paragraph. The relevant text commenting on this has been reproduced below for the reviewer's convenience:

"It is noteworthy that, despite the early mortality observed in Tg3 PBS males, the survival probability of this genotype was significantly higher notwithstanding higher MECP2 transgene expression than in the original study describing these mice where premature death was seen as

early as 2-4 weeks of age[3]. This suggests that the MDS phenotype in Tg3 mice has been significantly modified since the transgenic line was first studied."

7. Sentences such as the one at page 13 line 341-42 ("These levels across all four brain regions were close to ~50% of NTC controls, which we consider a tolerable yet disease-modifying level of efficacy") are inappropriate and should be revised, especially considering that Tg3 mice were found to display a ~8.4-fold increase in MeCP2 expression compared to wt controls.

We thank the reviewer for their comment and have removed the inappropriate speculation. The modified sentence is reproduced below:

"These levels across all four brain regions were close to ~50% of NTC controls (Figure 3B)."

8. The Authors should also explain how they choose to the specific tested dose.

The selected dose was based on historical data generated by our group and others using divalent siRNA for ICV injections. Multiple published studies (e.g., Belgrad et al. 2024 DOI: 10.1093/nar/gkae368; Weiss et al. 2025 DOI: 10.1016/j.ymthe.2025.05.010; Ferguson et al. 2024 DOI: 10.1002/alz.13703; Alterman, et al. 2019 DOI: 10.1038/s41587-019-0205-0) demonstrate that a total ICV dose of 20–40 nmol yields a duration of effect lasting six months, while a dose as low as 5 nmol provides potent silencing for at least one month post-injection.

This efficacy has been validated across multiple targets using the same divalent siRNA scaffold, supporting the versatility of the chemical scaffold and its translatability to the *mcep2*-targeting siRNA sequence. The text has been modified to include the dose selection rationale, reproduced below:

"Adult six-week-old male Tg3 mice were administered 12.5 nmol (~315 µg) of either 1764_S1, 112_S1, or phosphate-buffered saline (PBS) as a vehicle control via intracerebroventricular (ICV) injection. This dose was chosen based on previously published data on divalent siRNAs with similar potency[4-7]."

9. Why didn't the Authors perform comparisons with wt mice for survival and body weight measurements?

In the weight measurement, the WT-PBS group is not used as a statistical comparator because the purpose of the study was to measure the impact of siRNA treatment on the disease. The WT mice were used as an external control to ensure that the experimental methodology has no errors.

The statistical test was chosen specifically to answer the question of whether treatment was disease modifying, not whether the treatment brought the weight to WT 'non-disease' level.

With respect to survival, the reviewer is probably aware that ANOVA and multiple comparisons cannot be run on a Kaplan-Meier survival curve, which is a probabilistic analysis that provides

median survival as the output. The Kaplan-Meier survival curve shown in Fig. 4C can be used to compare survival across all groups without the need for any additional statistical analysis.

10. The Authors should further revise some statements throughout the text to more precisely reflect results. Some examples are:
a. “total MECP2 modulation through a single treatment with 1764_S1 at six weeks of age led to significant neurobehavioral phenotype recovery and completely reversed the premature mortality” - neurological measures were not performed on disase model mice treated at 6 weeks of age.

We apologize for the sentence not being clear which may have led to confusion. What is meant here is that a single treatment given at six weeks of age (the age at which mice were injected) led to neurobehavioral recovery (that was measured later). We have mentioned the ages at which neurobehavioral measures were performed in the relevant section. For the purposes of clarity, we have adjusted this text to the following:

“In conclusion, total MECP2 modulation through a single treatment with 1764_ led to some neurobehavioral phenotype recovery and rescued the premature mortality observed in both control and isoform-selective treatment groups.”

b. “Selective modulation of MECP2 E1 improved phenotypes initially” - the only phenotype significantly improved in the first 4 months is body weight, as other behavioural measures did not significantly differ from untreated Tg3 mice.

We have replaced ‘phenotypes’ with ‘body weight’ to be more specific. The edited paragraph is reproduced below:

“Selective modulation of MECP2 E1 improved body weight initially, however the effects diminished after four months without impacting overall survival.”

c. The Authors refer to a “1 year study period” while they evaluate treatment effect at 40 weeks maximum.
We agree with the reviewer. We have edited one year to forty weeks in the text. We have changed the title of the manuscript to remove the reference to one year as this is strictly not supported by the data.

d. “isoform-selective silencing using 112_S1 provided initial gains in weight and behavioral trends in Tg3 but lost efficacy after 19 weeks, despite multi-month durability in wildtype mice.” – the latest effects in wt mice were assessed at 22 weeks.

We thank the reviewer for this comment. We meant to say that the weight (measured more continuously) specifically was not improved after 19 weeks. We have modified the section to be more accurate. The edited text is reproduced below:

“E1 isoform-selective silencing using 112_S1 provided initial gains in weight only until 19 weeks, despite multi-month durability in wildtype mice.”

11. In general, the manuscript should be revised with more attention. For instance, since the experiments on Tg1 mice have been removed, all corresponding statements should likewise be deleted. Few examples are below:
a. One Sentence Summary: “Therapeutic siRNAs provide safe and durable modulation of MECP2 for the treatment of mild and severe MECP2 Duplication Syndrome”

This has been modified as below:

“Therapeutic siRNAs provide safe and durable modulation of MECP2 for the treatment of severe MECP2 Duplication Syndrome.”

b. Abstract: “Overall, this study introduces a series of preclinical candidates with the capacity to address the varying levels of MECP2 duplication encountered in clinical settings”

This has been modified as below:

“Overall, this study introduces a series of preclinical candidates for the treatment of MECP2 duplication. Furthermore, it establishes a target selection strategy that may be applied to other dosage-sensitive gene imbalances.”

c. Results: Page 17, line 416-417 “we turned to a more severe MDS model, the Tg3 mice”

The word ‘more’ has been removed. The sentence now reads, “we turned to a severe MDS model…”

Reviewer #4 (Remarks to the Author):

I co-reviewed this manuscript with one of the reviewers who provided the listed reports. This is part of the Nature Communications initiative to facilitate training in peer review and to provide appropriate recognition for Early Career Researchers who co-review manuscripts.

1. Weiss, A., *et al.* RNAi-mediated silencing of SOD1 profoundly extends survival and functional outcomes in ALS mice. *Mol Ther* **33**, 3917–3938 (2025).
2. Khvorova, A. & Watts, J.K. The chemical evolution of oligonucleotide therapies of clinical utility. *Nat Biotechnol* **35**, 238–248 (2017).
3. Collins, A.L., *et al.* Mild overexpression of MeCP2 causes a progressive neurological disorder in mice. *Hum Mol Genet* **13**, 2679–2689 (2004).

4. Belgrad, J., *et al.* A programmable dual-targeting siRNA scaffold supports potent two-gene modulation in the central nervous system. *Nucleic Acids Res* **52**, 6099–6113 (2024).
5. Weiss, A., *et al.* RNAi-mediated silencing of SOD1 profoundly extends survival and functional outcomes in ALS mice. *Mol Ther* **33**, 3917–3938 (2025).
6. Ferguson, C.M., *et al.* Silencing Apoe with divalent-siRNAs improves amyloid burden and activates immune response pathways in Alzheimer's disease. *Alzheimers Dement* **20**, 2632–2652 (2024).
7. Alterman, J.F., *et al.* A divalent siRNA chemical scaffold for potent and sustained modulation of gene expression throughout the central nervous system. *Nat Biotechnol* **37**, 884–894 (2019).

Response to reviewer #3

We thank the reviewer for their comments which have certainly improved the quality of this manuscript. We have removed the phrase “completely modifies” as suggested.
